# Drivers of Population Genetic Diversity Across Ecologically Distinct Species in a Cape Fynbos Grass Clade

**DOI:** 10.1111/mec.70342

**Published:** 2026-04-12

**Authors:** Maryam Burger, George Anthony Verboom, Lara Misty Wootton, Justin James van Blerk, Seth Daniel Musker

**Affiliations:** ^1^ Centre for Statistics in Ecology, Environment and Conservation, Department of Statistical Sciences University of Cape Town Rondebosch South Africa; ^2^ Department of Biological Sciences University of Cape Town Rondebosch South Africa; ^3^ Department of Biology and Environmental Science University of Gothenburg Gothenburg Sweden; ^4^ Gothenburg Botanical Garden Västra Gotalandsregionen Gothenburg Sweden; ^5^ Bolus Herbarium University of Cape Town Rondebosch South Africa; ^6^ LECA, CNRS, University Grenoble Alpes, University Savoie Mont Blanc Grenoble France

**Keywords:** Cape Floristic Region, climate variation, habitat preference, population genetic diversity, reproductive traits

## Abstract

Population genetic diversity (PGD) underpins the adaptive capacity of species, shaping resilience to environmental change and long‐term persistence. Yet the drivers of PGD variation are complex and difficult to disentangle because they act across multiple scales and reflect both ecological context and evolutionary history. Here, we investigate PGD in the *Ehrharta setacea* Clade (Poaceae), an ecologically and morphologically diverse lineage endemic to the Cape Floristic Region of South Africa. Using genotyping‐by‐sequencing data, we test the relative influence of species traits, regional climate gradients, habitat hydrology and metapopulation connectivity on spatial patterns of PGD. Our analyses reveal a strong species effect, indicating that evolutionary history and niche‐defining traits, particularly habitat preference and reproductive investment, are fundamental determinants of PGD. Wet‐habitat species consistently maintain higher PGD than dry‐habitat species, while spikelet length emerges as an important predictor of genetic diversity across both habitat types. Regional climate variation also plays a role, with PGD associating positively with mean annual precipitation and negatively with interannual precipitation variability. Population isolation is linked to reduced PGD, with outlier populations exhibiting especially low diversity. Together, these findings highlight how species biology, regional climate, and metapopulation processes interact to structure PGD and emphasise the southwestern CFR as a critical hotspot for its conservation.

## 1 Introduction

**FIGURE 1:**
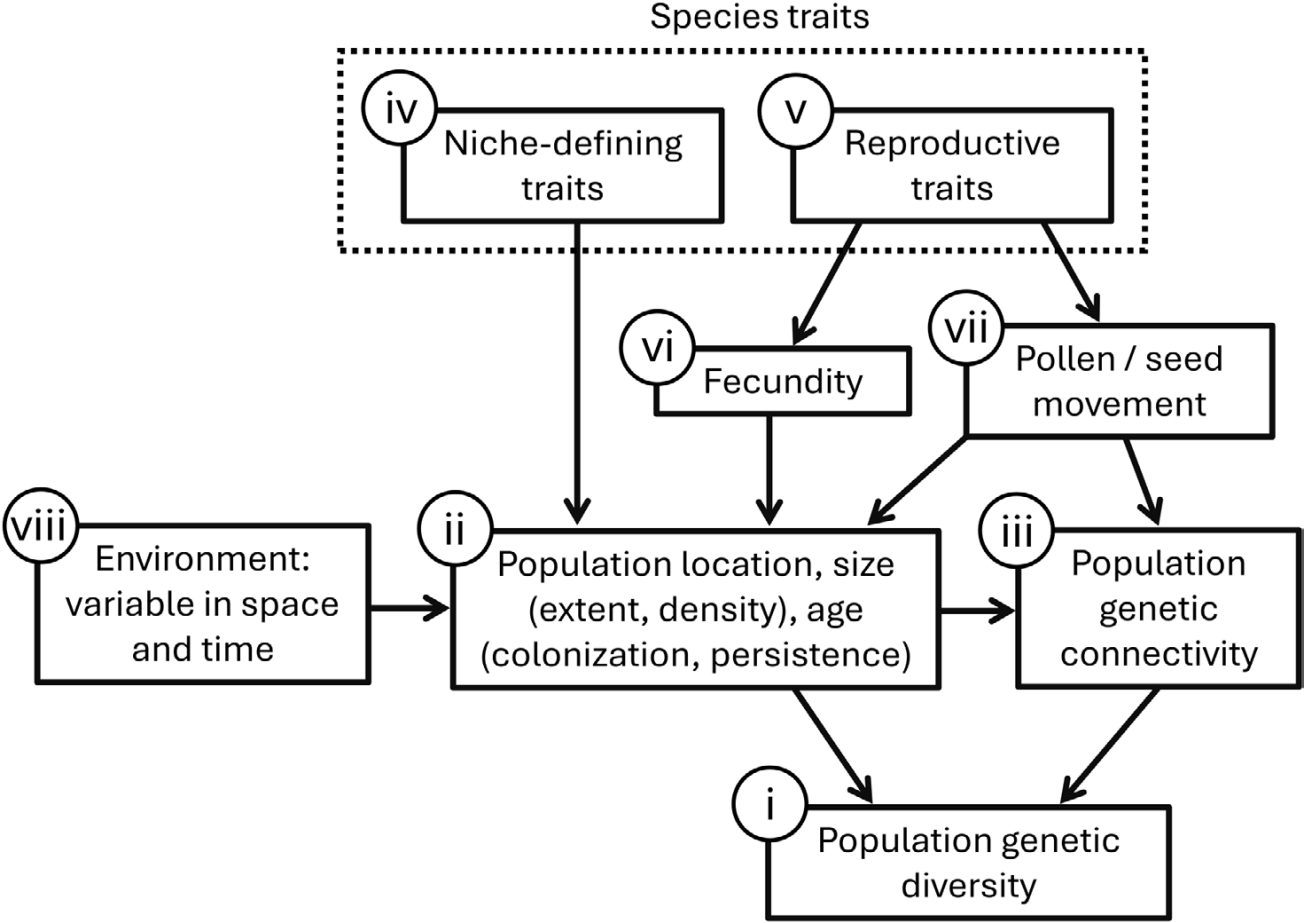
Conceptual framework of determinants of population genetic diversity. Factors investigated in this study are numbered (i) to (viii) and correspond to references in the text.

The genetic diversity of populations is evolutionarily important, determining the ability of populations to adapt to a changing environment (Booy et al. 2000; Reed and Frankham 2003; Frankham 2005; Ellegren and Galtier 2016) and so influencing the ability of species to persist in the face of such change (Amos and Balmford 2001; Reed and Frankham 2003; Spielman et al. 2004; Kardos et al. 2021). Understanding the determinants of population genetic diversity (PGD) in wild plant species is challenging, however, with local PGD (Figure 1: i) being a product of both population history and demography (Figure 1: ii) and the movement of genes within and between populations (Figure 1: iii). The location, size, and age of populations is a product of the spatio‐temporal distribution of vital resources that characterise the species' niche (Hutchinson 1991; Abbott et al. 2000; Pulliam 2000; Martínez‐Meyer et al. 2013). Consequently, local PGD may be expected to correlate strongly with niche‐defining traits (Figure 1: iv). Reproductive traits (Figure 1: v) are also important, through their effect on fecundity (Herrera 1991, 1993; Figure 1: vi) and, consequently, population size and persistence (Conlisk et al. 2012; Cayuela et al. 2016; Figure 1: ii). Beyond their demographic impacts, reproductive traits, particularly mating system and dispersal‐related traits, also influence PGD both by enabling the establishment of new populations (seed dispersal; Figure 1: ii) and by facilitating seed‐ and pollen‐mediated gene exchange within and between existing populations (Levin 1981; Sork and Smouse 2006; Wright et al. 2008; Goodwillie et al. 2010; Barrett 2015; Browne et al. 2018; Figure 1: iii). This gene flow has the effect of connecting local populations into higher‐level entities, commonly termed metapopulations (Levins 1969; Levin 1981; Hanski 1991; Abbott et al. 2000; Sork and Smouse 2006), whose cohesiveness has consequences for local PGD (Figure 1: i). All these processes, of course, play out in an environment which is variable in both space and time (Figure 1: viii).

Since the traits that determine niche attributes, fecundity, and seed/pollen movement vary between species, PGD should and does vary between species. For example, the broad ecological niches of generalist species may render them more abundant, more widely distributed, and more resilient to environment change, whereas the narrower niches of specialist species may be associated with range‐restriction and increased susceptibility to environmental fluctuations (McKinney 1997; Colles et al. 2009; Sexton et al. 2009). This has consequences for PGD, with generalists showing generally greater PGD than specialists (Li et al. 2014; Pasinelli 2022). Analogously, increased investment in sexual reproduction and the possession of traits that promote outcrossing and gene flow enhance genetic diversity, with sexual populations of many plant species having greater PGD than asexual populations (Booy et al. 2000; Pollux et al. 2007), and outcrossing species having generally greater PGD than selfing species (Wright et al. 2008; Huang et al. 2021). At least in biotically pollinated species, floral display, determined as the product of flower number and flower size, may serve as an effective indicator of outcrossing rate (Goodwillie et al. 2010), while increased investment in seed or propagule production may be expected to enhance dispersal ability (Westoby et al. 1996; Soons et al. 2004; Thomson et al. 2011).

Beyond traits, PGD is also influenced by environment, through the effect of the latter on critical resource availability and thus population size (Wright 1931). Since most environments are dynamic, population size is likewise dynamic, with periods of low or localised resource availability usually marked by population isolation, contraction (Jones et al. 2023) and even, potentially, extinction. In woody species, for example, drought events induce widespread mortality (Condit et al. 1995; Anderegg et al. 2019), with consequences for local population size and persistence. Where demographic fluctuations are severe, they leave long‐lasting genetic signatures, typically in the form of reduced PGD, this effect being exacerbated where local population extinction is followed by recolonisation, owing to the founder effect (Pironon et al. 2015). Since the magnitude of environmental dynamism is spatially variable, PGD varies considerably, being high in demographically stable refugial sites and lower in non‐refugial areas (Hewitt 2004). The peripheral refugia of the European Alps constitute a classic example, providing conditions that buffered plant populations against the extreme cold of the Pleistocene glaciations, and so preserving their genetic diversity (Schönswetter et al. 2005). Not all refugia are temperature refugia, however, with hydrological buffering being an important feature of refugia in many semi‐arid or mediterranean‐type ecosystems (McLaughlin et al. 2017). Here, refugia can range in scale from regional (10^4^–10^6^ m) to microsite (10^1^–10^3^ m), depending on whether they owe their existence to regional climate or to local factors (e.g., microtopography).

Given the influence of multiple factors operating at different scales and interacting in complex ways (Figure 1), disentangling the determinants of PGD is challenging. Focusing on a small clade of grasses native to the fynbos vegetation of the Cape Floristic Region (CFR) of South Africa, we assess the drivers of PGD variation in a manner that considers both the unique attributes of species, including their microhabitat preferences and reproductive traits, and the influence of broad gradients of climate and climate stability. The CFR presents an excellent system for exploring the determinants of PGD, owing to the presence of a well‐documented longitudinal climate stability gradient which may account for a corresponding longitudinal plant species richness gradient (Cowling and Lombard 2002; Cowling et al. 2017). Although the entire CFR has enjoyed relative climate stability through the Pleistocene (Dynesius and Jansson 2000; Cowling et al. 2004; Bradshaw et al. 2014), the winter‐rainfall west appears to have been more stable than the aseasonal east (Chase and Meadows 2007), potentially accounting for its greater total and locally endemic species richness. That the richness in the west is concentrated on specific mountain ranges (e.g., the Kogelberg and Hottentots‐Holland Mountains; Linder 2019), however, suggests that it is not explicable with reference to a broad longitudinal climate stability gradient alone. An alternative is that the topography, geology, and saturating winter precipitation of the southwestern mountains together produce a unique, groundwater‐based hydrology that is to some extent decoupled from regional climate fluctuations (Bradshaw et al. 2014) and so functions as a hydrological refugium. Consistent with this idea, recent work identifies these mountains as a centre of distribution for species that associate with groundwater‐fed habitats (Van Blerk, Cramer, et al. 2025) and as a centre of palaeoendemism (Van Blerk, Verboom, et al. 2025).

Towards understanding the drivers of PGD variation, we examined the spatial distribution of PGD and its correlates in the CFR‐endemic, rice‐related *Ehrharta setacea* Clade (Poaceae, Oryzoideae). Historically considered to comprise just two species, *E. rupestris* Nees and *E. setacea* Nees, genomic data have recently revealed the presence of 11 species (Wootton et al. 2023). While species in this clade are generally moisture‐loving, they vary in microhabitat, comprising seven species that associate obligately with swampy, groundwater habitats and four that associate with drier situations (Wootton et al. 2023). Despite all being mat‐forming, clonal perennials, these species vary considerably in the extent to which they invest in sexual reproduction, in the height at which the inflorescences are borne (15–75 cm), in inflorescence size (1–14 spikelets per inflorescence), and in spikelet size (4–7.5 mm long) and number (1–16).

Here we use genotyping‐by‐sequencing (GBS; Elshire et al. 2011) data to examine the relative importance of (i) species' niche‐defining traits, (ii) species' reproductive traits, (iii) regional variation in climate stability and precipitation, and (iv) genetic connectivity and colonisation history in shaping the distribution of PGD in the *E. setacea* Clade. Consistent with Figure 1, we predict that PGD is greatest in areas of high climate stability, reflecting longer‐term demographic persistence and earlier colonisation, and that it is also influenced positively by association with drought‐buffered surface‐groundwater habitats, by greater investment in outcrossing and dispersal, and by proximity to conspecific populations and/or the clade's geographic centre of origin.

## 2 Materials and Methods

### 2.1 Population Sampling

Two hundred and fifty‐seven (257) individual plants were sampled from 43 populations (i.e., localities), with four to six individuals sampled per population and a single accession per population kept as a voucher. Following Wootton et al. (2023), who used an integrative taxonomic approach involving multiple sources of genetic data, plus morphological, ecological and geographic data, to redraw species boundaries in the *E. setacea* Clade, these populations fall into 11 well‐supported species. Our sampling captures the full distribution range of the *E. setacea* Clade and includes all species, the number and distribution of populations sampled per species broadly reflecting the relative abundance of each species across its range (Wootton et al. 2023). To avoid sampling clones, individuals at each population locality were sampled to be at least 30 m apart. The sampled populations range from 35 to 1686 m above sea level, and span both longitudinal climate stability (Herrando‐Moraira et al. 2022) and precipitation seasonality gradients (Bradshaw et al. 2014). All species inhabit sandstone‐derived soils.

### 2.2 DNA Extraction, Sequencing, and Genotyping

The genotype‐by‐sequencing (GBS) data generated by Wootton et al. (2023; *n* = 92 samples) were supplemented with sequences generated from 165 additional, silica‐dried leaf samples collected by those authors from the same populations. DNA was extracted using a modified CTAB protocol (Doyle and Doyle 1987). Library preparation and DNA sequencing were performed by Novogene Genome Sequencing Company Ltd. (Beijing, China). Library preparation was done as per Wootton et al. (2023), with sequencing done in a paired‐end manner on an Illumina NovaSeq 6000 sequencer, with a read length of 150 bp. Raw demultiplexed reads were filtered using fastp version 0.23.4 (Chen 2023) to remove adapters and low‐quality reads. Reads were mapped to a draft genome of *E. rupestris* subsp. *rupestris* using bwa‐mem2 version 2.2.1 (Vasimuddin et al. 2019) with default parameters.

#### 2.2.1 Genetic Diversity

Genetic diversity was quantified as individual‐level genome‐wide heterozygosity (IGH) using ANGSD v. 0.940‐dirty (Korneliussen et al. 2014), which is equivalent to nucleotide diversity at the level of a single diploid individual (Nei and Li 1979). For this purpose, default parameters were used to estimate individual site allele frequency tables, from which the site frequency spectrum was estimated using realSFS (Nielsen et al. 2012), again with default parameters and heterozygosity calculated as the proportion of segregating sites out of the total number of genotyped sites.

#### 2.2.2 Genotyping

Genotype data were used for phylogenetic analysis and estimation of genetic divergence. For phylogenetic inference, an individual of *E. rehmannii* for which GBS data were available (Wootton et al. 2023) was selected as an outgroup, the choice of outgroup (within *Ehrharta*) being arbitrary since the *E. setacea* Clade is sister to the rest of the genus (Wootton et al. 2023; Figure S1). Genotype calling and filtering was done using BCFtools v. 1.1 (Danecek et al. 2021). Individual genotypes with depth < 4 were recoded as missing and sites with > 30% missing data and total depth < 200, as well as indels, were removed. Thereafter, the genotype call data were converted to FASTA format using vcf2phylip v. 2.9 (Ortiz 2019). Heterozygous sites were encoded using IUPAC ambiguity codes. Only biallelic SNPs with minor allele frequency ≥ 0.05 were used for estimation of genetic divergence.

### 2.3 Phylogenetic Tree Inference

The optimal substitution model was estimated using ModelFinder (Kalyaanamoorthy et al. 2017) and the maximum likelihood phylogeny was estimated using IQ‐TREE2 v. 2.2.2.6 (Minh et al. 2020). Branch support values were estimated using ultrafast bootstrap (UFBoot; Hoang et al. 2018) with 1000 replicates. To generate an ultrametric tree with relative branch lengths, we used wLogDate v1.0.2 (Mai and Mirarab 2021) with 10 independent replicates and the alignment length set to the total number of genotyped sites (*n* = 4,016,198). For phylogenetic analysis, accessions representing each population were collapsed to a single terminal once population monophyly had been confirmed.

### 2.4 Species Attributes

#### 2.4.1 Habitat Type

The habitat descriptions provided by Wootton et al. (2023) (see Table S1) were used to score each species as associating either with swampy, groundwater‐fed habitats (wet) or with habitats that are seasonally dry (dry). Scoring of this variable was independent of the population locality characteristics, including site hydrology (below).

#### 2.4.2 Sexual Investment and Gene Flow Traits

Three phenotypic traits, plant height (including inflorescence; PLH), spikelet number (SPN), and spikelet length (SPL), were scored to quantify species' sexual investment and capacity for between‐population gene flow. Where plant height is expected to affect gene flow through its influence on both pollen (Sork and Smouse 2006; Goodwillie et al. 2010) and seed movement (Soons et al. 2004; Thomson et al. 2011), spikelet size and number, as proxy measures of seed size and number, describe investment in sexual reproduction and dispersal (Venable 1992; Westoby et al. 1996; Pollux et al. 2007), the two traits being positively correlated in the *E. setacea* Clade (*r* = 0.51, *p* < 0.001). Data for the four traits were obtained from Wootton et al. (2023), who scored two to four flowering individuals per population.

### 2.5 Site Characteristics

#### 2.5.1 Long‐Term Climate Variability

The long‐term climate variability of each population locality over the past 3.3 Myr was quantified using the climate stability index of Herrando‐Moraira et al. (2022), here referred to as a climate instability index (CII) because large values correspond to conditions of low stability. CII is the sum of the standard deviations of each of 14 bioclimatic variables relating to precipitation and temperature, over 12 time periods spanning the last 3.3 Myr with a resolution of approximately 5 km. It is derived using data from the WorldClim (Fick and Hijmans 2017) and PaleoClim (Brown et al. 2018) climate models, the former based on weather station and satellite data and the latter on CHELSA data (Karger et al. 2017). Site‐specific CII values were extracted using population coordinates and the extract function in the terra package version 1.7.71 (Hijmans 2020). Although previous work has found patterns of population genetic differentiation to be related to the long‐term stability of the precipitation seasonality gradient in the CFR (Prunier et al. 2017), CII correlates strongly with both longitude (*r* = 0.91, *p* < 0.001) and precipitation seasonality (*r* = 0.74, *p* < 0.001) in our data. Therefore, we only included CII as a covariate in our analyses.

#### 2.5.2 Contemporary Precipitation Regime

Mean annual precipitation (MAP) for the 2006–2020 period was determined for each population locality using precipitation data from the Multi‐Source Weighted‐Ensemble Precipitation (MSWEP) v. 2.8 0.1° global monthly rainfall data set (Beck et al. 2019). In addition, year‐on‐year contemporary precipitation variability was determined as the coefficient of variation of annual precipitation (CVAP) at each site for the period January 2006 to December 2020. This period is of particular interest because it captures an intense and sustained drought event (2016–2017; Wolski 2018). Data from before 2006 were excluded because visual inspection of the monthly rainfall maps for that time revealed large patches of uniform rainfall which we assumed to be artefactual.

#### 2.5.3 Site Hydrology

As a proxy measure of soil hydrology, we determined the height (elevation) of each population locality above the nearest drainage line (HAND; Nobre et al. 2011), reasoning that localities with high HAND values should, on average, have less saturated soils. HAND was determined using the MERIT Hydro global hydrography map v. 1.0.1 (Yamazaki et al. 2019), which has a 3‐arc sec resolution (ca. 77 m at 34° S).

### 2.6 Geographic Isolation

#### 2.6.1 Geographic Isolation

The geographic distance of each population from the nearest sampled conspecific population (ISO_C), was quantified as a measure of its potential genetic connectivity. For this purpose, populations were determined to be conspecific using the species conceptualisations of Wootton et al. (2023), and the ISO_C of each population determined as the great circle distance separating it from the nearest conspecific population. Great circle distances were calculated using the R package sf v. 1.0‐21 (Pebesma 2018), with ISO_C only being determined for species sampled from three or more populations. The use of geographical distance as a measure of genetic connectivity is justified by positive isolation by distance (IBD; Wright 1943) trends in most species (Table S2), although these were typically non‐significant owing to sample size limitation, cf., Jenkins et al. (2010). Of the species examined, only Setacea, sampled from 11 sites, showed a significant IBD pattern (Table S2: *p* = 0.002), the remainder being sampled from three to five sites each. Isolation by distance tests used Jost's D (Jost 2008), calculated using the R package vcfR v. 1.15.0 (Knaus and Grünwald 2017) as a measure of genetic distance and log‐transformed great circle distance. Significance was assessed using Mantel tests (Mantel 1967) as implemented in the R package vegan v. 2.7‐1 (Oksanen et al. 2007).

#### 2.6.2 Distance From Origin

As an estimate of the amount of historical migration experienced by populations, we determined the geographical distance of each population from their inferred centre of origin of the *E. setacea* Clade (ISO_O). For this purpose, the centre of origin was inferred as the phylogenetic centroid of the clade, using the R package phytools v. 2.5.2 (Revell 2024). The ISO_O of each population was determined as the great circle distance separating it from this phylogenetic centroid.

### 2.7 Regression Analyses

All regression analyses were carried out using Bayesian multilevel linear modelling with Stan v2.36 (Gelman et al. 2015), as implemented in the R package brms v. 2.22.0 (Bürkner 2017), with the joint posterior sampled using Hamiltonian Monte Carlo (Neal 2011). All models were checked for convergence by inspecting R‐hat (Vehtari et al. 2021) and effective sample size (ESS) for each parameter. Models were checked for goodness of fit using posterior predictive checks with the brms pp_check function. Unless otherwise specified, default priors were used, and models were fit using four chains of 2000 iterations each (1000 burn‐in, 1000 sampling), with hyperparameters adapt_delta and max_treedepth adjusted upwards from the brms defaults as needed to achieve convergence. Parameters and derived quantities were summarised and visualised using the R package ggdist v. 3.3.2 (Kay 2023).

#### 2.7.1 Quantifying Among‐Species Variation

We aimed to assess the degree to which trait and environmental variation was associated with (i) species and (ii) habitat. For the species effect, we used regression‐based variance decomposition in which, for each variable, a model was fit with a global intercept and a random effect of species. To test whether wet‐ and dry‐habitat species differed in mean and among‐species standard deviation, we fit models with separate intercepts and random effects per habitat and calculated the posterior difference (wet minus dry) as a derived quantity of the full posterior distribution (Holsinger and Wallace 2004).

#### 2.7.2 Phylogenetic/Taxonomic Signal

To estimate the degree of phylogenetic and taxonomic (i.e., species‐associated) signal in genetic diversity, we used a Bayesian phylogenetic mixed model approach akin to classical ANOVA. With IGH as the response variable, we fitted a maximal model with an overall intercept, varying intercept terms (i.e., random effects) for species and population (to account for repeated measures), as well as a phylogenetically structured varying intercept term for population which incorporates a phylogenetic correlation matrix. The latter was produced using the R package ape v. 5.8 (Paradis and Schliep 2019) and the ultrametric phylogeny pruned to contain one tip per population. We used a Gaussian response distribution and specified standard half‐normal priors on the group‐level standard deviation terms and the global sigma. To compare the variance explained by taxonomic and phylogenetic effects, we fitted models excluding each in turn. The variance attributed to the phylogenetic effect provides an estimate of phylogenetic signal, termed heritability, which is similar to Pagel's lambda but differs in that it can only take values between zero and one (Freckleton et al. 2002; Housworth et al. 2004).

#### 2.7.3 Single‐Predictor Regression Analysis

To estimate the unconditional effect of each predictor variable on heterozygosity we fitted separate models in which IGH was specified as a function of each predictor variable (Table 1), with separate intercepts and slopes for wet‐ and dry‐habitat species. We accounted for repeated measures by adding a population‐level random effect, and for phylogenetic autocorrelation by adding a phylogenetically structured population‐level random effect (as above). Since morphological traits were available for only a subset of individuals (*n* = 164), missing values (*n* = 93) were imputed during model fitting using the brms mi functionality. Imputation was informed by a phylogenetically structured individual‐level random effect, with the correlation matrix derived from the individual‐level ultrametric phylogeny, as well as an unstructured population‐level random effect and species identity as a fixed effect. Under this approach, imputation is done during model fitting, so that uncertainty is naturally incorporated into the model. Missing values for all traits were imputed using Gaussian response distributions, and residual correlation was also modelled to correct for regression dilution due to measurement error. Models with a morphological variable predictor had the form “bf(H ~ 1 + Habitat*mi(X) + (1|Population) + (1|gr(Population, cov = Cp))) + bf(X | mi() ~ 1 + Species + (1|Population) + (1|gr(Individual, cov = Ci))) + set_rescor(TRUE)”, where H is heterozygosity, X is the continuous predictor variable, and Cp and Ci are the population‐ and individual‐level phylogenetic correlation matrices, respectively.

**TABLE 1:** Description of predictor variables used in regression analyses.

Variable	Description
IGH	Individual‐level genome‐wide heterozygosity
Habitat	Categorical (wet/dry)—Species level
MAP	Mean annual precipitation 2006–2020; MSWEP 0.1° (10 km by 10 km)
HAND	Elevation above nearest drainage line (77 m resolution)
CVAP	Coefficient of variation of annual precipitation 2006–2020; MSWEP 0.1° (10 km by 10 km)
CII	Summed standard deviation of climate variables, 5 km resolution
SPL	Spikelet length: Length from the base of spikelet to the tip of longest lemma (mm)
SPN	Spikelet number: Count of spikelets in a representative inflorescence
PLH	Plant height: Length from rhizome to inflorescence tip (mm)
ISO_C	Distance from nearest conspecific population (km)
ISO_O	Distance from phylogenetic centroid

#### 2.7.4 Multi‐Predictor Regression Analysis

To estimate the conditional effects of each predictor variable, we formulated two multi‐predictor linear regression models. Model 1 had only main effects and no interactions, i.e., all terms were added individually, while Model 2 included all main effects as well as an interaction term between each continuous variable and habitat type, with separate intercepts and slopes per habitat type. We formulated two versions of each model, one specifying population phylogenetic relationships among populations as a random effect (henceforth, “Phylogenetic linear mixed‐effect (LME)” models) and the other specifying species identity as a random effect (henceforth, “species LME” models). All models included a non‐phylogenetic random effect of population identity to account for non‐independence of measurements. Model 1 had the form “H ~ 1 + Habitat + X + (1|Population) + (1|gr(Population, cov = C))”, while Model 2 had the form “H ~ 0 + Habitat + Habitat:(X) + (1|gr(Population, by = Habitat)) + (1|gr(Population, cov = C, by = Habitat)), sigma ~ 0 + Habitat”, where H is heterozygosity, sigma is the residual standard deviation, X represents the set of continuous predictor variables, and C is the population‐level phylogenetic correlation matrix. Missing values for the three morphological traits were imputed in the same manner as was done for the univariate regressions. Additionally, missing ISO_C values, which were not determined for species with fewer than three populations (*n* = 6), were imputed using an intercept‐only Gaussian model. We placed standard normal priors on model coefficients, standard half‐normal priors on standard deviation and sigma terms, and an lkj_corr_cholesky(2) prior on the residual correlation matrix. The latter moderately penalises extreme correlation coefficients.

All continuous predictor variables were mean‐centred to zero and variance‐scaled to one, except for ISO_C and ISO_O, which were first log‐transformed before centering and scaling. Doing so provided two advantages for model interpretation. Firstly, intercepts correspond to the expected heterozygosity of a population when predictors are at their mean. Secondly, scaling allows the effect of all predictors to be interpreted on the same scale, with slopes corresponding to the effect of the predictor increasing by one unit of standard deviation. To estimate the conditional linear effect (i.e., slope) of each predictor variable on heterozygosity we used the avg_slopes function in the R package marginaleffects v. 0.21.0 (Arel‐Bundock et al. 2024). For Model 2, the effect of each predictor was estimated separately for each habitat type by specifying “by = ‘habitat’” within the call to avg_slopes. The posterior_draws function was used to derive posterior draws of slopes. A walk‐through of the multi‐predictor analyses can be found in Data S1.

## 3 Results

### 3.1 Taxonomic and Phylogenetic Signal

**FIGURE 2:**
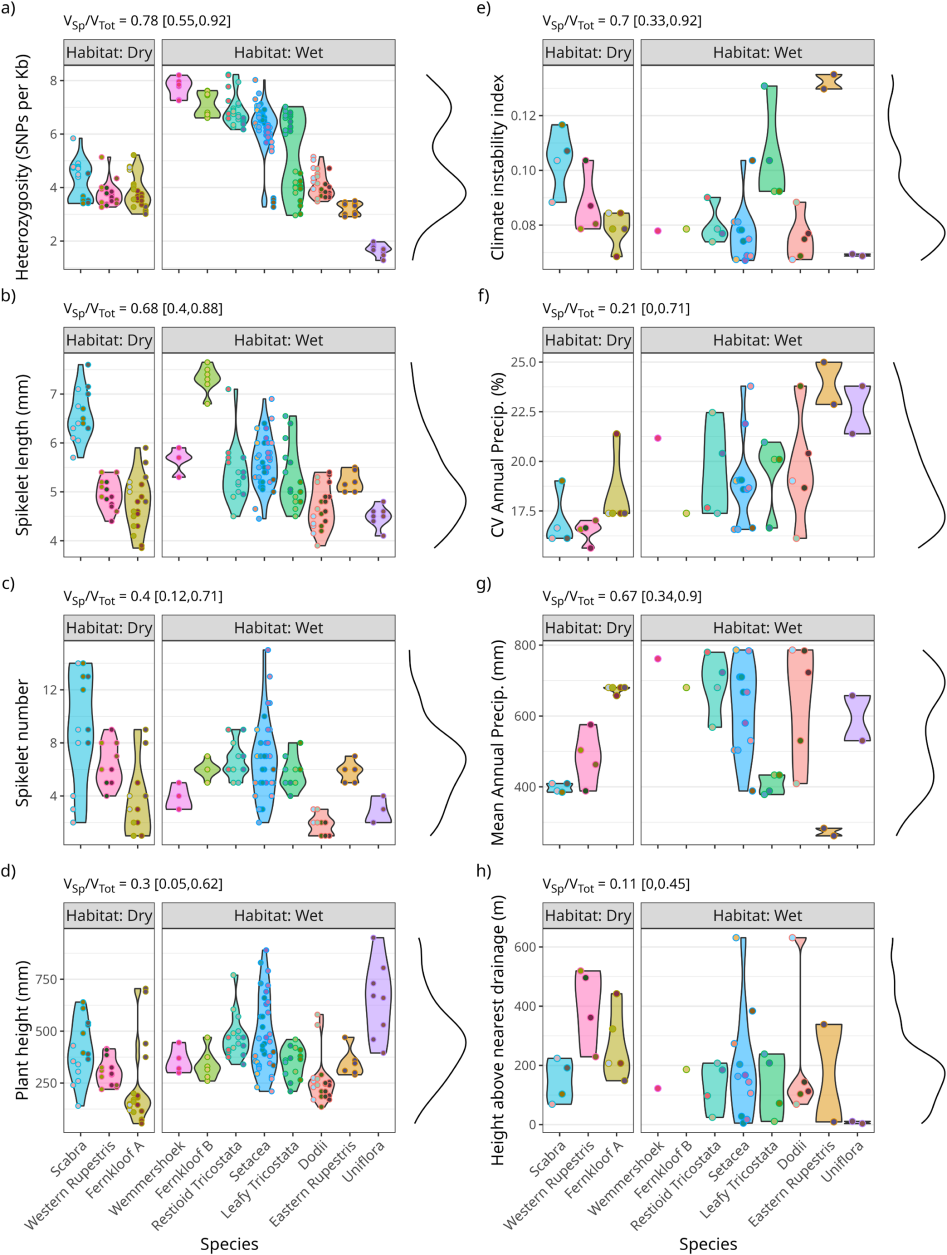
Variation among individuals, populations, and species in genetic diversity (a) and morphological trait variables (c,d), and variation among species and populations in environmental variables (e–h). Point colours within each violin plot are population specific. The proportion of total variance attributable to species is indicated above each plot as the mean and 95% credible interval. V_Sp_ = variance among species, V_Tot_ = total variance.

**FIGURE 3:**
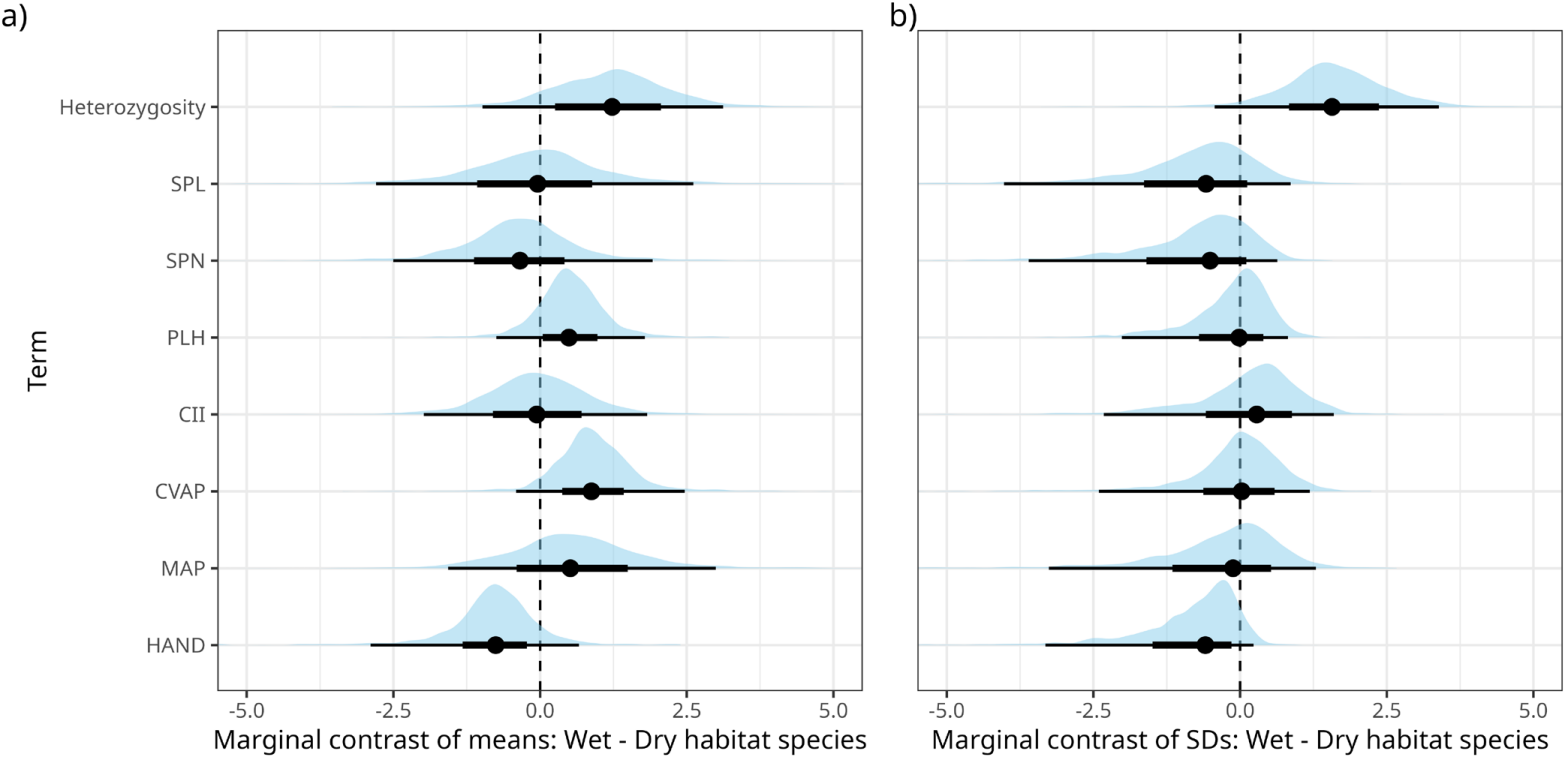
Posterior distributions of pairwise comparisons between wet and dry habitat species for each variable considered in our analyses, in terms of (a) difference in mean and (b) difference in standard deviation among species. The direction of the effect is determined by Wet—Dry, i.e., positive differences occur when the wet habitat species have a higher mean or among‐species standard deviation than dry habitat species.

Species identity explains a substantial proportion of the total variation observed in both organismal traits and environmental/niche variables (Figure 2). Despite varying fourfold (< 2 to > 8 SNPs per Kb) across the *E. setacea* Clade (Figure 2a), for example, IGH is relatively constant within species, with species identity accounting for 70% of total IGH variation. Leafy Tricostata and Setacea are unusually variable with respect to IGH, with high IGH being the norm in Setacea and low IGH in both species being a feature of populations in geographically marginal or isolated sites (Figure 6: LT1, LT2 and SE11). Beyond IGH, species accounts, respectively, for 69%, 40% and 30% of total variation in SPL, SPN and PLH, and for 70%, 66% and 21% of total variation in CII, CVAP and MAP (Figure 2e–g). On the evidence of SPL and SPN, Fernkloof A, Dodii and Uniflora invest the least in sexual reproduction, and Scabra and Setacea the most (Figure 2b,c). Wet‐ and dry‐habitat species differ marginally in IGH (mean and SD higher in wet‐habitat species), CVAP (mean higher in wet‐habitat species), and HAND (mean and SD lower in wet‐habitat species; Figure 3). Taken together, these data confirm the biologically differentiated nature of species in the *E. setacea* Clade, both with respect to organismal traits and environmental niche attributes.

Except for Fernkloof A, which is paraphyletic, GBS data resolve the species defined by Wootton et al. (2023) in the *E. setacea* Clade to be monophyletic with strong support (Figure S2). Similarly, with the sole exception of the Fernkloof B populations, which are inter‐nested and so treated as a single population in subsequent analyses, populations are also monophyletic. Given these patterns and the general importance of species as a predictor of variation in many of the traits/variables studied, the manifestation of phylogenetic signal in many of these variables (Figure S3) is unsurprising. The correspondence between species effect strength and phylogenetic signal is imperfect, however, since the latter additionally considers covariance due to between‐species relationships. Where IGH, CII, MAP, SPL, and SPN all show strong phylogenetic signal, as evidenced by lambda close to 1, PLH shows moderate signal. For all these variables, however, the confidence intervals on lambda exclude zero, indicating the presence of signal. By contrast, CVAP, HAND, and ISO_C show little evidence of phylogenetic signal, their lambda confidence intervals including zero. The presence of a strong species effect and phylogenetic signal in several variables, including the response variable IGH, confirms the need to account for the species effect or phylogeny when examining relationships among these variables.

### 3.2 Predictors of Population Genetic Diversity

A model assessing the relative contributions of phylogeny vs. species effect as determinants of population mean IGH, hereafter termed population genetic diversity (PGD), identifies species as the most important predictor of PGD (ca. 65% of PGD variance; Figure S4). With the species effect excluded, however, phylogeny explains a greater proportion of PGD variance than does species with phylogeny excluded (85% compared to 75%). Unsurprisingly, given the correspondence between species effect strength and phylogenetic signal, the PGLS and species LME models yield highly similar results for both single‐ and multi‐predictor analyses (Figures S5 and S6) and only the results of PGLS regressions are therefore reported below.

Phylogenetic LME regression identifies habitat type as a strong predictor of PGD, with wet‐habitat species displaying generally higher PGD than dry‐habitat species (Figure 3). In addition, wet‐habitat species display greater among‐species variation in PGD than dry‐habitat species (Figure 3b). Across all samples PGD displays a bimodal distribution (Figure 2a) with a high‐PGD mode formed exclusively by wet‐habitat species (*n* = 5) and a low‐PGD mode formed by the three dry‐habitat species and the three remaining wet‐habitat species (i.e., Dodii, Eastern Rupestris, and Uniflora). The range‐restricted pond‐specialist Uniflora has the lowest PGD of all species, followed by Eastern Rupestris which inhabits localised damp sites in the arid fynbos of the Swartberg. Besides PLH, HAND and CVAP are also influenced by habitat type, wet‐habitat species being on average taller and occupying sites having lower HAND and higher CVAP (Figure 3a). Where the first relationship supports our use of HAND as an indicator of site hydrology, the second suggests a role for wet microsites in buffering wet‐habitat species against precipitation fluctuations. Importantly, the significant effect of habitat‐type on PGD necessitates that habitat type is accounted for when evaluating the influence of other variables and traits on PGD.

**FIGURE 4:**
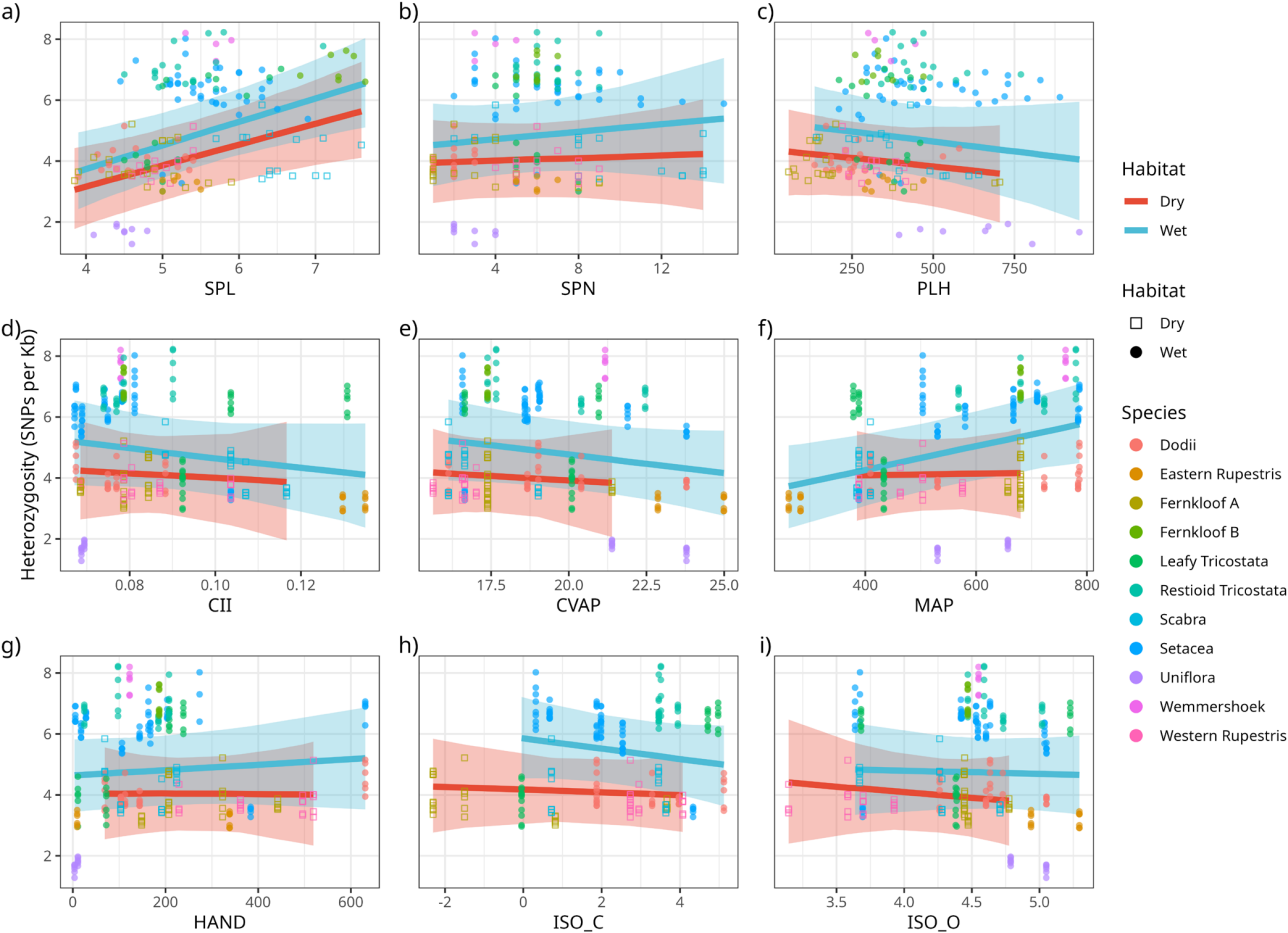
Relationship between genetic diversity and each covariate (a–i), for dry (red, squares) and wet (blue, circles) habitat species using phylogenetic LME regression models. Point colour is species‐specific.

Single‐predictor phylogenetic LME regressions, applied separately to dry‐ and wet‐habitat species, generally reveal weak or no relationships of PGD to individual predictor variables, except for MAP and SPL, which exhibit relatively strong positive relationships with PGD (Figure 4a,f). The effect of MAP, however, is only apparent in wet‐habitat species. While the slopes of regression models relating PGD to CII, CVAP, ISO_C, and ISO_O match prior expectations, the associated confidence intervals are too wide to render them meaningful (Figure 4d,e,h,i). The weak negative relationship of PGD to PLH (Figure 4c) is unexpected.

**FIGURE 5:**
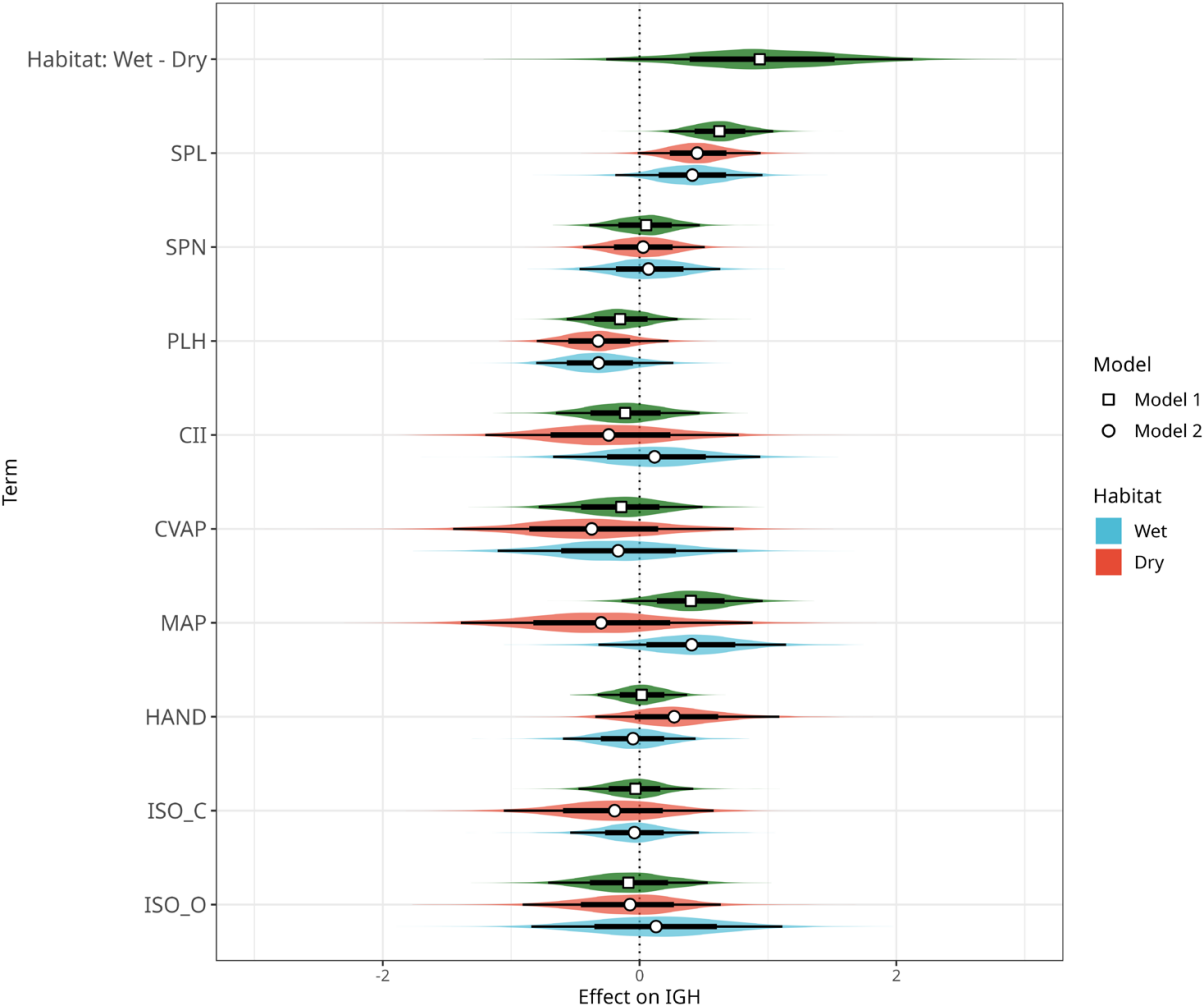
Posterior density distributions of slope estimates for multiple regression models 1 (green, squares) and 2 (blue and red, circles). With Dry as the reference category for habitat in Model 1, the first coefficient Habitat:Wet is the difference between wet and dry‐habitat populations. Model 2 included habitat as an interaction term, thus, all slopes for model 2 are by habitat (blue = wet, red = dry). Points represent the mode, and thicker and thinner line ranges represent the 66% and 95% highest density intervals, respectively. Estimates correspond to the effect of a one standard deviation increase in the predictor variable on IGH (in SNPs per Kb).

Multi‐predictor phylogenetic LME regression analyses identify habitat type, MAP, CVAP, and SPL as important predictors of PGD (Figure 5). Habitat type emerges as the strongest single predictor of PGD, with wet‐habitat species having generally greater PGD than dry‐habitat species (Figure 5; model 1). Moreover, corroborating the single‐predictor regressions, multi‐predictor regression reveals strong effects of MAP, CVAP, and SPL on PGD. Inclusion of habitat as an interaction term reveals that the positive effect of MAP and negative effect of CVAP is exclusive to wet habitat species, while the positive effect of SPL on PGD is observed in both wet‐ and dry‐habitat species (Figure 5; model 2). Since SPN and SPL are positively associated (*r* = 0.51, *p* < 0.001), removal of either term does not substantially alter the estimated effect of the other on PGD (results not shown). Contrary to expectation, PLH has a negative effect on PGD, although this is weak, being associated with large uncertainty. Similarly, while the estimated effects of HAND, ISO_C, and ISO_O are close to zero, they are all associated with and have large uncertainty. The slight negative relationships observed in the single‐predictor analyses are not supported by the multi‐predictor analyses.

### 3.3 Geographic Distribution of PGD

**FIGURE 6:**
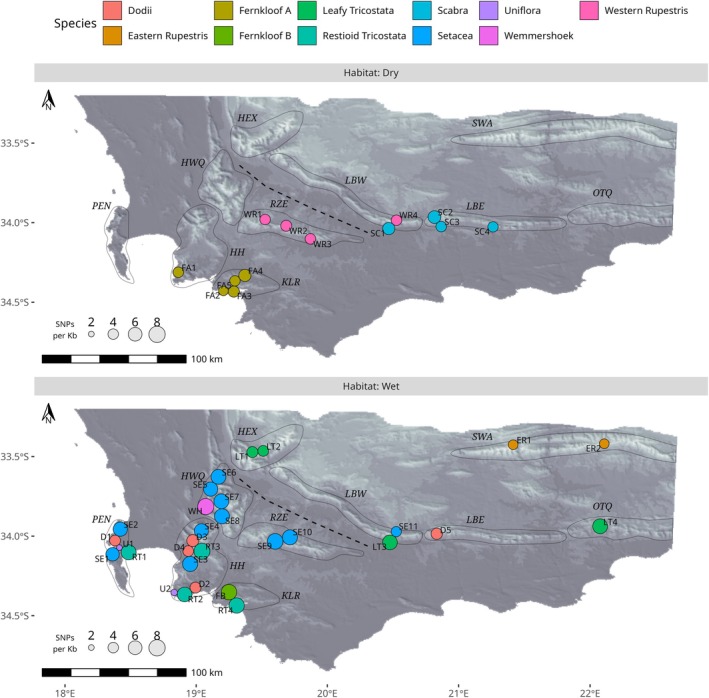
Distribution of population genetic diversity, by species (colour), separated by habitat preference (top and bottom panels). Circle area is proportional to mean heterozygosity. Circle locations have been minimally adjusted to avoid overlaps. Coloured labels indicate accession/population ID. Labels indicate accession/population ID. Mountain regions are outlined (HEX: Hex River; HH: Hottentots Holland; HWQ: Hawequas; KLR: Klein Rivier; LBE: Langeberg East; LBW: Langeberg West; OTQ: Outeniqua; PEN: Cape Peninsula; RZE: Riviersonderend; SWA: Swartberg.)

PGD varies with geography (Figure 6), being greatest on the mountains of the southwestern CFR, specifically the Peninsula, Hottentots‐Holland, Kleinrivier and Hawequas Mountains, and lower towards the east and interior. This pattern is conditional on habitat‐type, being apparent in wet‐habitat species but not in dry‐habitat species, which have generally lower PGD. Among the wet‐habitat species, Dodii, Fernkloof A and Uniflora are exceptional in having low PGD despite being largely restricted to the southwestern mountains. Geographical variation in PGD is also observed within the two widespread wet‐habitat species, Leafy Tricostata and Setacea. In both these species, PGD variation is bimodally distributed (Figure 2a) with low PGD being a feature of outlying populations which are geographically isolated from the rest of the species. In the case of Leafy Tricostata, which occurs primarily along the Langeberg, low PGD is associated with an outlying population, represented by two closely adjacent localities (LT1, LT2) on the Hex River Mountains. In the case of Setacea, which occurs primarily on the southwestern mountains, low PGD is a feature of site SE11 which, besides being geographically distant, is also isolated from the rest of the species by a prominent low‐elevation dispersal barrier, the Breede River Valley (Figure 6).

## 4 Discussion

Understanding the amount and geographic distribution of PGD in the *E. setacea* Clade is challenging owing to the influence of multiple interacting factors that describe both the evolutionary history and ecological context of species and populations making up this lineage. Our data reveal a strong dependence of PGD on the unique species attributes that are an outcome of evolutionary history, as captured by phylogeny and species identity. Specifically, we find PGD to be highly species‐specific, likely due to species‐level differences in habitat preference (Figure 1: iv) and sexual investment (Figure 1: v), both of which directly affect population size, persistence, and genetic connectivity (Figure 1: ii, iii). Regional climate variation also appears important; however, PGD is influenced by both annual precipitation amount (MAP) and year‐on‐year precipitation variability (CVAP; Figure 1: viii). Although we find no evidence of an effect of colonisation history (ISO_O), PGD is sensitive to geographic isolation of populations (Figure 1: iii), with outlier populations in widespread species showing unusually low PGD. The multifaceted and multiscale character of the processes that shape the amount and distribution of PGD in the *E. setacea* Clade is effectively represented by Figure 1.

### 4.1 Niche‐Defining Traits

Traits that define a species' niche affect PGD in a fundamental manner, by controlling both the location, extent, and density of local populations and consequently population continuity in space and time (Figure 1; Pulliam 2000; Sexton et al. 2009; Pasinelli 2022; Jones et al. 2023). This is apparent in the *E. setacea* Clade, in which habitat preference (wet vs. dry) emerges as an important predictor of PGD, modulating the effect of both regional climate and reproductive traits on PGD (below). The general trend of higher PGD in wet‐habitat species compared to those exposed to seasonal dryness suggests greater population demographic stability and persistence in wet habitats (Reed and Frankham 2003), which is consistent with the latter being more stable hydrologically (McLaughlin et al. 2017). Furthermore, given that population abundance and continuity is expected to be greater in areas of high habitat suitability (Martínez‐Meyer et al. 2013), the concentration of high‐PGD populations in the southwestern mountains of the CFR matches expectation given the prevalence of moist, groundwater‐fed habitats there (Van Blerk, Cramer, et al. 2025).

Our crude categorisation of habitat type as wet vs. dry, however, belies the subtle but significant habitat variation that distinguishes different wet‐ or dry‐habitat species in the *E. setacea* Clade (Wootton 2020; Wootton et al. 2023) but which nonetheless has implications for population size and connectivity (McKinney 1997; Colles et al. 2009). Specialisation to extreme habitats, for example, is generally associated with small and isolated populations, and correspondingly low PGD (Young et al. 1996; McKinney 1997; Booy et al. 2000; Colles et al. 2009; Schierenbeck 2017; Pasinelli 2022). Within the *E. setacea* Clade, extreme specialisation is exemplified by Uniflora, a species which associates with permanently inundated sites in which the plants root underwater and clamber up the stems of *Psoralea* shrubs for support and access to light (Wootton 2020; Wootton et al. 2023). Together with the extreme adaptive challenges that such a lifestyle presents and the reproductive trade‐offs it may necessitate (Silvertown et al. 2015), the small and isolated nature of pond habitats imposes constraints on population size and extent, with consequences for PGD. Population isolation may similarly help to explain the generally low PGD of dry‐adapted species, such as Fernkloof B and Western Rupestris, which associate with high‐elevation ridges along the south‐facing mountain ranges of the CFR (i.e., Hottentots‐Holland, Kleinrivier, Riviersonderend, and Langeberg Mountains). By virtue of the linear structure of these mountain ranges and the presence of low elevation features that function as dispersal barriers during dry periods, populations of these species are prone to episodic isolation (Britton et al. 2014; Verboom et al. 2015).

### 4.2 Reproductive Traits

Beyond niche‐defining traits, traits that determine fecundity and the potential for gene exchange also contribute to PGD through their effects on population demography and population genetic connectivity (Figure 1; Booy et al. 2000; Soons et al. 2004; Goodwillie et al. 2010; Ellegren and Galtier 2016; Schierenbeck 2017). In this study, we quantify investment in sexual reproduction and outcrossing potential using three traits: plant height (PLH), spikelet length (SPL), and spikelet number (SPN). Where all three traits are expected to influence seed‐mediated gene flow, SPL, as a proxy for seed size, is expected to correlate with an ability to maintain larger, more extensive, and more demographically stable populations (Booy et al. 2000; Pollux et al. 2007; Cochrane et al. 2015). This is because large seeds show greater germination success (Moles and Westoby 2004; Souza and Fagundes 2014) and confer enhanced seedling resilience (Gross 1984; Leishman and Westoby 1994; Westoby et al. 1996; Leishman 2001).

Across all analyses, and in both wet‐ and dry‐habitat species, SPL is found to exert a strong positive effect on PGD and, while SPN is unrelated to PGD, a positive association between SPL and SPN means that the first effectively represents both variables, thus serving as a joint measure of both seed number and size. While a positive correlation between SPL and SPN is unexpected given the well‐established trade‐off between seed size and number (Venable 1992; Leishman 2001; Lazaro and Larrinaga 2018), the relationship of these traits is modulated by other factors, such as life history and ecological context (Westoby et al. 1996; Moles and Westoby 2004; Qiu et al. 2022). Regardless, the dependence of PGD on SPL (and SPN) implies that, despite the generally strong reliance of *E. setacea* Clade species on clonal persistence (Verboom et al. 2003; Wootton et al. 2023), maternal investment in sexual reproduction enhances fecundity and gene exchange, with consequences for PGD. Among the wet‐habitat species, for example, Setacea and Scabra, both of which have high SPL and high SPN, display generally high PGD, while Uniflora and Dodii, with their diminutive, few‐flowered inflorescences (low SPL and SPN), display consistently low PGD.

While we expected inflorescence height (PLH) to influence metapopulation connectivity positively, through its effect on the spatial movement of both seeds and pollen (Burd and Allen 1988; Okubo and Levin 1989), we find little evidence of such an effect. This is likely because the mean height difference between the tallest and shortest species in this study (ca. 50 cm) is insufficient to counteract the effect of other factors that are more influential. For example, our detection of a weak but slightly negative relationship between PGD and PLH appears to be influenced strongly by the highly‐localised hydrophyte, Uniflora, whose inflorescence height is attributable to a very unusual clambering habit. The low PGD in Uniflora is probably associated with its highly localised pond habitat.

### 4.3 Regional Climate

While organismal traits clearly play an important role in shaping PGD, landscape‐scale variation in climate and climate stability are also expected to shape PGD through their effects on the long‐ and short‐term demographic stability of populations (Figure 1; Cowling and Lombard 2002; Hewitt 2004; Harrison and Noss 2017). Accordingly, we find that although wet‐habitat species in the *E. setacea* Clade show generally higher PGD than dry‐habitat species, PGD is positively associated with MAP and negatively associated with CVAP among wet‐habitat species. This implies that the hydrological stability of wet habitats, and the demographic stability of the species they support, is sensitive to regional climate gradients, with reliable annual rainfall or at least an absence of severe drought events being required to ensure that groundwater recharge is sufficient to produce water discharge at the soil surface (McLaughlin et al. 2017; Nenweli et al. 2024). In the CFR, these conditions appear to hold most strongly in the southwestern mountains which, besides being a centre of PGD for members of the *E. setacea* Clade, is a centre of diversity for wet habitat‐adapted fynbos plants (Van Blerk, Cramer, et al. 2025). The extent to which the precipitation‐sensitivity of PGD in wet‐habitat *E. setacea* Clade species is a general feature of swamp‐adapted fynbos plant species remains uncertain, given the trait‐dependence of hydrological stability (McLaughlin et al. 2017). For example, where the shallow rooting systems of grasses render them dependent on the surface discharge of groundwater, this may not be true for deeper‐rooted species (Groom 2004; Garssen et al. 2014).

The relative climate‐insensitivity of dry‐habitat *E. setacea* species on precipitation regime probably reflects a reduced reliance of these species on surface water for leaf cooling. Owing to their shallow root systems, the occupation of dry, open habitats presents leaf thermoregulatory challenges for grasses, with the consequence that broad‐leaved grasses tend to associate with shady habitats (Gallaher et al. 2019). In Cape *Ehrharta*, which have an ancestral association with damp habitats (Verboom et al. 2003, 2004; Kellogg 2009) and commonly possess flat, expanded leaves (Russell 1987), this challenge appears to be variously solved by growing in shady settings (e.g., *E. erecta* , *E. rehmannii*, *E. stipoides* ), by summer leaf loss (Verboom et al. 2003), by inhabiting damp sites which support transpirational leaf cooling (wet‐habitat *E. setacea* Clade species), and by inhabiting windy, high‐elevation sites which support convectional air cooling (dry‐habitat *E. setacea* Clade species). Importantly, because air cooling is not moisture dependent, the latter species are comparatively insensitive to precipitation regime.

While initially surprising, our failure to detect an impact of long‐term climate instability (CII) on PGD may be an outcome of several factors. One possibility is that the spatial resolution of the CII data is too coarse (5 km) to capture the fine spatial scale of the habitats with which many *E. setacea* species typically associate (Schwalm et al. 2016). Another is that the long timeframe (3.3. Myr) of the CII data set is inappropriate, with the genetic signature of deeper historical events being overwritten and determined by more recent (e.g., contemporary) climatic events (Schierenbeck 2017). The latter explanation is supported by the stronger association of PGD with MAP and CVAP.

### 4.4 Genetic Connectivity

Although slight, there is a tendency for widespread species in the *E. setacea* Clade to display high PGD (e.g., Setacea and Leafy Tricostata), perhaps reflecting the contribution of metapopulation structure and between‐population gene exchange as a determinant of local PGD (Vandewoestijne et al. 2008; Vrijenhoek 2010; Snead et al. 2024). This suggestion gains further support from the observation that within both Setacea and Tricostata, geographically and topographically isolated populations (SE11 in the case of Setacea; LT1 and LT2 in the case of Tricostata) show reduced PGD relative to the remaining populations. Not all widespread species show high PGD, however, possibly indicating a lack of between‐population gene exchange. For example, despite being fairly widespread, Dodii displays consistently low PGD, perhaps because the highly localised nature of its damp cliff habitats selects against investment in sexual reproduction and effective dispersal (Barrett 2015). There is, however, no evidence of a global linear effect of geographic isolation on PGD.

Metapopulation genetic theory predicts that the reduction of genetic diversity due to geographic isolation (reduced gene flow or founder effects) may only manifest once effective connectivity or gene flow drops below a critical level, and genetic drift overwhelms migration (Wright 1931; Hanski 1998). This is supported by empirical evidence showing that PGD is often resilient to moderate fragmentation but declines sharply under extreme or long‐term isolation (Lowe et al. 2005; Keyghobadi 2007). On the other hand, there are many sources of uncertainty in quantifying geographic isolation. For example, our methods of quantifying ISO_C and ISO_O assume comprehensive sampling, which cannot be guaranteed, although all known populations in the clade were sampled. Additionally, even comprehensive sampling of extant populations does not account for the possibility that connectivity has been maintained until recently by now‐extinct geographically intermediate populations.

## 5 Conclusion

In a manner consistent with Figure 1, our analyses reveal an influence of niche‐defining traits, reproductive traits, regional climate gradients, and population isolation on PGD in the *E. setacea* Clade. A strong signal of evolutionary history (species effect and phylogeny), however, introduces significant analytical complexity, making it difficult to disentangle the effects of the other factors. Since species, even closely related species like those studied in this paper, possess contrasting functional attributes and ecological behaviours, we suspect that this situation is general and recommend that PGD variation be studied, wherever possible, in the context of single species, preferably species lacking significant population structure. Unfortunately, this presents a challenge for studies of PGD in the CFR, since widespread species are the exception there (Cowling and Holmes 1992), with even traditionally recognised widespread species often being revealed by genomic data to contain substantial population genetic structure corresponding to locally adapted forms (Lexer et al. 2014; Prunier et al. 2017; Galuszynski and Potts 2020) or even species (Shaik et al. 2023; Wootton et al. 2023). In this context, students of PGD variation in the CFR may have little choice but to rise to the challenge and account for this historical signal as we have attempted to do here.

The emergence of the southwestern CFR as a hotspot of PGD in the *E. setacea* Clade, together with its identification as a centre of wet‐habitat fynbos species richness (Van Blerk, Cramer, et al. 2025), narrow‐range endemism (Cowling and Lombard 2002), phylogenetic diversity (Forest et al. 2007; Colville et al. 2020), palaeoendemism (Van Blerk, Verboom, et al. 2025), and plant species richness (Oliver et al. 1983; Cowling et al. 2017), suggests that it has functioned historically as a refugium, especially for moisture‐sensitive fynbos species. Further supporting this perspective is its identification as a key source area for colonisation of Restionaceae into the rest of the CFR (Wüest et al. 2019). Since areas which have functioned as refugia in the past may continue to function as biodiversity safehouses under contemporary climate change (Keppel et al. 2012; Harrison and Noss 2017), they are priorities for biodiversity conservation (Keppel et al. 2012; Tolley et al. 2019). In that context, increasing efforts to abstract water from the southwestern mountains of the CFR to supply a thirsty Cape Town (Slingsby et al. 2018; Pascale et al. 2020) is a cause for serious concern.

## Author Contributions

M.B., S.D.M., G.A.V. and J.J.B. conceptualized the paper; M.B., S.D.M. and L.M.W. collected the data; M.B. and S.D.M. ran the analyses; M.B., S.D.M. and G.A.V. wrote the paper; J.J.B. and L.M.W. provided comments on the paper.

## Funding

This work is based on research supported by the National Research Foundation of South Africa (Grant Number: 137970).

## Conflicts of Interest

The authors declare no conflicts of interest.

## Supporting information


**Table S1:** Habitat descriptions of each species within the *Ehrharta setacea* species complex, by Wootton et al. (2023). All species are generally wet‐loving, but some associate with seasonally dry habitats (“Dry”).
**Table S2:** Results of the Mantel tests for isolation by distance between populations within species. The number of permutations (no. perm) and number of population pairs (no. pairs) involved in the tests are indicated.
**Figure S1:** Phylogenetic tree of the Ehrharta rupestris/setacea Clade, including all populations sequenced in this study and the outgroup E. rehmannii. Branch lengths are unitless and represent relative time. All branches had bootstrap (UFBoot) > 95%. All sampled populations were monophyletic. Outgroup is shown in the top right section, labelled REH LW34.
**Figure S2:** Phylogenetic tree of the Ehrharta rupestris/setacea Clade, including all populations sequenced in this study. Heterozygosity (heterozygous sites per megabase) is indicated by the size and fill of the circles at the tips. Branch lengths are unitless and represent relative time. All branches had bootstrap (UFBoot) > 95%. All sampled populations were monophyletic.
**Figure S3:** Posterior distributions for phylogenetic signal for each predictor variable. All variables were scaled and centred prior to analysis. ISO here refers to the geographic distance to the nearest conspecific population (ISO_C in the main text).
**Figure S4:** Plots showing the posterior distribution of explained variance in IGH partitioned by population (unstructured), phylogeny (phylogenetically structured population effect), species, and residual variation, for three models including alternative combinations of these terms.
**Figure S5:** Relationship between genetic diversity and each covariate (a–h), for dry (red, squares) and wet (blue, circles) habitat species using univariate regression with species as a random effect (species LME models). Point colour is species‐specific.
**Figure S6:** Posterior density distributions of slope estimates for multiple regression for the Species LME models 1 (green, squares) and 2 (blue and red, circles). With Dry as the reference category for habitat in Model 1, the first coefficient Habitat:Wet is the difference between wet and dry‐habitat populations. Model 2 included habitat as an interaction term, thus, all slopes for model 2 are by habitat (blue = wet, red = dry). Points represent the mode, and thicker and thinner line ranges represent the 66% and 95% highest density intervals, respectively. Estimates correspond to the effect of a one standard deviation increase in the predictor variable on IGH (in SNPs per Kb).

**Data S1:** Model‐heterozygosity

## Data Availability

The sequence data for this study have been deposited in the European Nucleotide Archive (ENA) at EMBL‐EBI under accession number PRJEB98992. The data that support the findings of this study are openly available in ZivaHub at http://doi.org/10.25375/uct.30316216. Model code can be found in Data S1.
