# Supplementary material for: Drivers of Population Genetic Diversity Across Ecologically Distinct Species in a Cape Fynbos Grass Clade
Source: Mol Ecol. 2026 Apr 12;35(8):e70342. doi: 10.1111/mec.70342 (PMC13071368; doi:10.1111/mec.70342)
Supplement: Supplementary file 1 — Table S1: Habitat descriptions of each species within the Ehrharta setacea species complex, by Wootton et al. (2023). All species are generally wet‐loving, but some associate with seasonally dry habitats (“Dry”). Table S2: Results of the Mantel tests for isolation by distance between populations within species. The number of permutations (no. perm) and number of population pairs (no. pairs) involved in the tests are indicated. Figure S1: Phylogenetic tree of the Ehrharta rupestris/setacea Clade, including all populations sequenced in this study and the outgroup E. rehmannii. Branch lengths are unitless and represent relative time. All branches had bootstrap (UFBoot) > 95%. All sampled populations were monophyletic. Outgroup is shown in the top right section, labelled REH LW34. Figure S2: Phylogenetic tree of the Ehrharta rupestris/setacea Clade, including all populations sequenced in this study. Heterozygosity (heterozygous sites per megabase) is indicated by the size and fill of the circles at the tips. Branch lengths are unitless and represent relative time. All branches had bootstrap (UFBoot) > 95%. All sampled populations were monophyletic. Figure S3: Posterior distributions for phylogenetic signal for each predictor variable. All variables were scaled and centred prior to analysis. ISO here refers to the geographic distance to the nearest conspecific population (ISO_C in the main text). Figure S4: Plots showing the posterior distribution of explained variance in IGH partitioned by population (unstructured), phylogeny (phylogenetically structured population effect), species, and residual variation, for three models including alternative combinations of these terms. Figure S5: Relationship between genetic diversity and each covariate (a–h), for dry (red, squares) and wet (blue, circles) habitat species using univariate regression with species as a random effect (species LME models). Point colour is species‐specific. Figure S6: Posterior density distributions of s [file MEC-35-e70342-s001.pdf]

SUPPLEMENTARY MATERIALS 1

Table S1. Habitat descriptions of each species within the Ehrharta setacea species complex, by Wootton et al (2023). All species are generally wet-loving, but some associate with seasonally dry habitats ("Dry").

Species	Habitat type	Detailed description
Scabra	Dry	Dry slopes and disturbed areas
Western Rupestris	Dry	Rocky summit ridges
Fernkloof A	Dry	Seasonally dry slopes
Wemmershoek	Wet	Damp slopes
Fernkloof B	Wet	Moist, boggy areas
Restioid Tricostata	Wet	Seeps
Setacea	Wet	Moist, boggy areas
Leafy Tricostata	Wet	Seeps or stream sides
Dodii	Wet	Base of cliffs and rocky overhangs
Eastern Rupestris	Wet	Moist depressions on north-facing slopes
Uniflora	Wet	Damp, boggy areas in the understory of dense Psoralea stands

Table S2. Results of the Mantel tests for isolation by distance between populations within species. The number of permutations (no. perm) and number of population pairs (no. pairs) involved in the tests are indicated.

Species	Correlation (r)	p-value	no. perm	no. pairs
Dodii	0.642	0.108	119	10
Fernkloof A	0.586	0.083	119	10
Leafy Tricostata	0.846	0.083	23	6
Restioid Tricostata	-0.547	0.833	23	6
Scabra	-0.324	0.625	23	6
Setacea	0.492	0.002	999	55
Western Rupestris	0.886	0.208	23	6

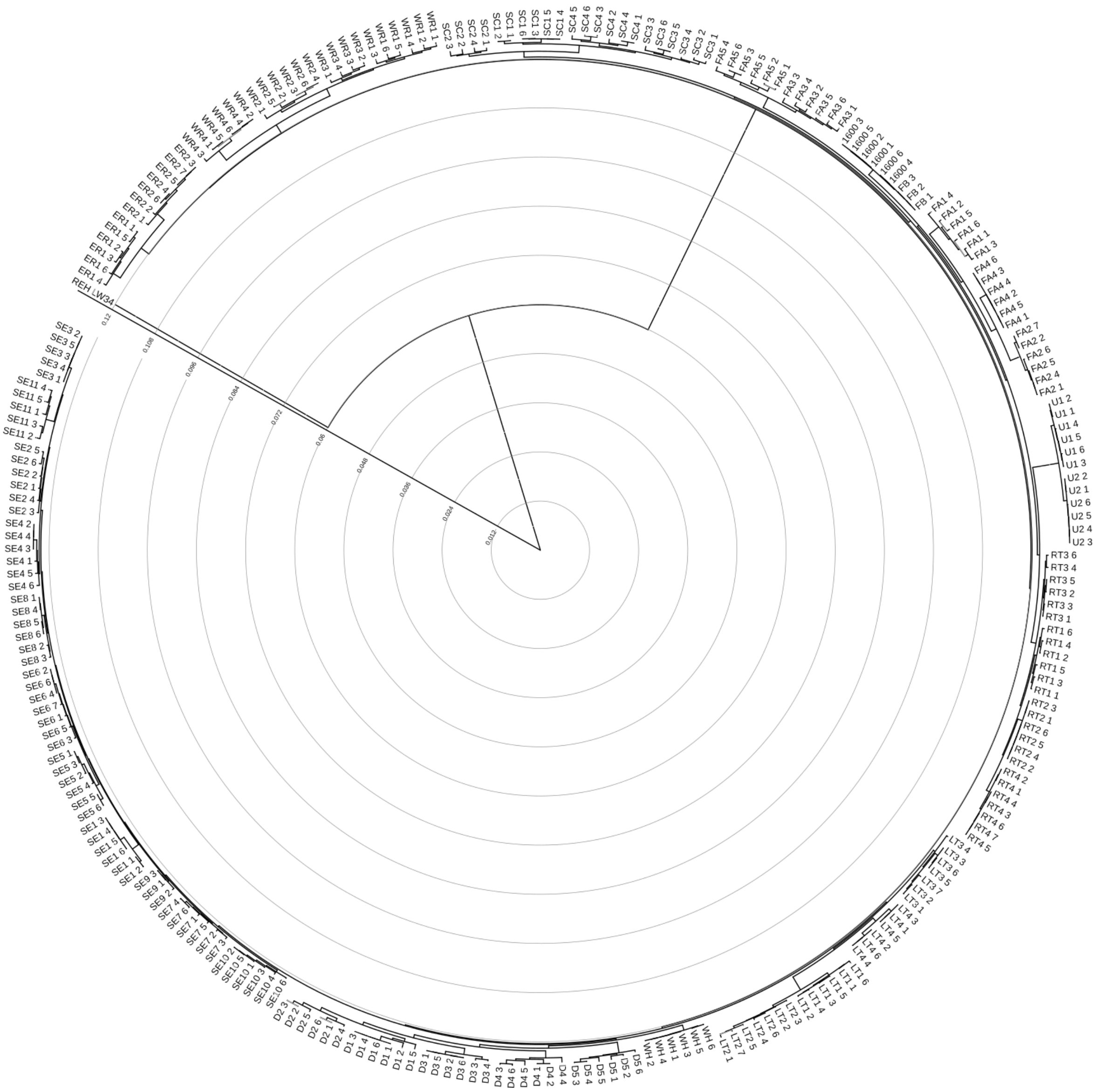

Figure S1. Phylogenetic tree of the *Ehrharta rupestris*/*setacea* Clade, including all populations sequenced in this study and the outgroup *E. rehmannii*. Branch lengths are unitless and represent relative time. All branches had bootstrap (UFBoot) > 95%. All sampled populations were monophyletic. Outgroup is shown in the top right section, labelled REH LW34.

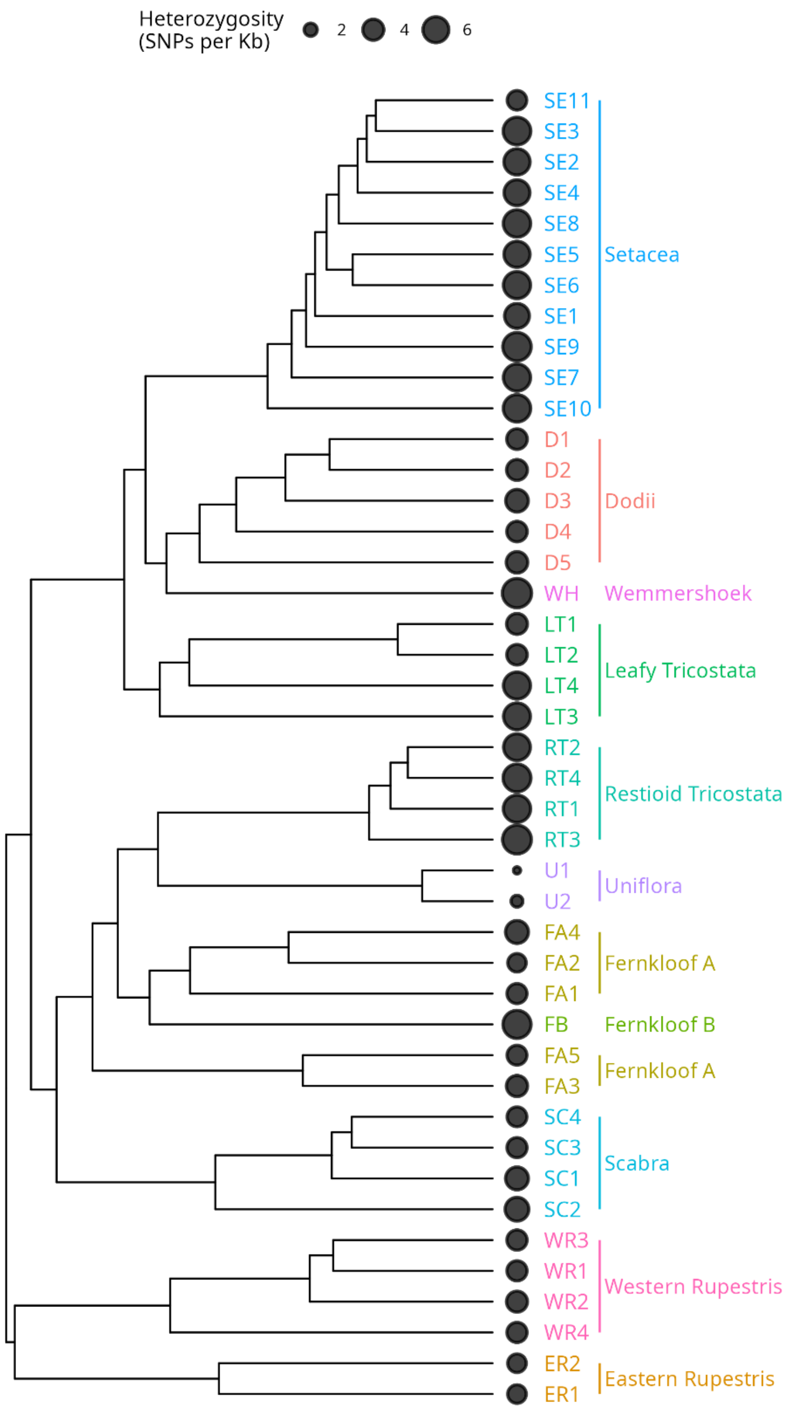

Figure S2. Phylogenetic tree of the *Ehrharta rupestris/setacea* Clade, including all populations sequenced in this study. Heterozygosity (heterozygous sites per megabase) is indicated by the size and fill of the circles at the tips. Branch lengths are unitless and represent relative time. All branches had bootstrap (UFBoot) > 95%. All sampled populations were monophyletic.

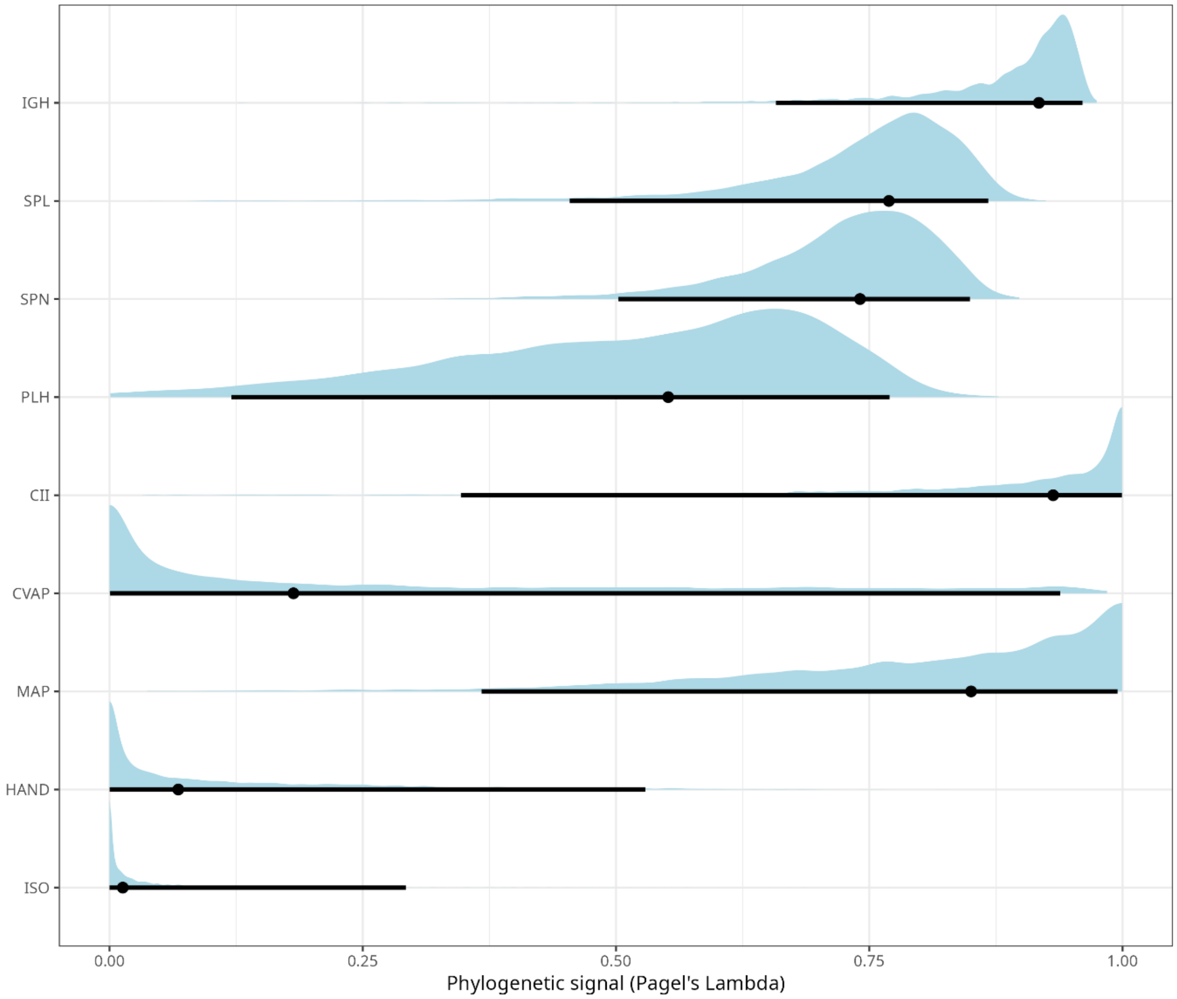

Figure S3. Posterior distributions for phylogenetic signal for each predictor variable. All variables were scaled and centred prior to analysis. ISO here refers to the geographic distance to the nearest conspecific population (ISO_C in the main text).

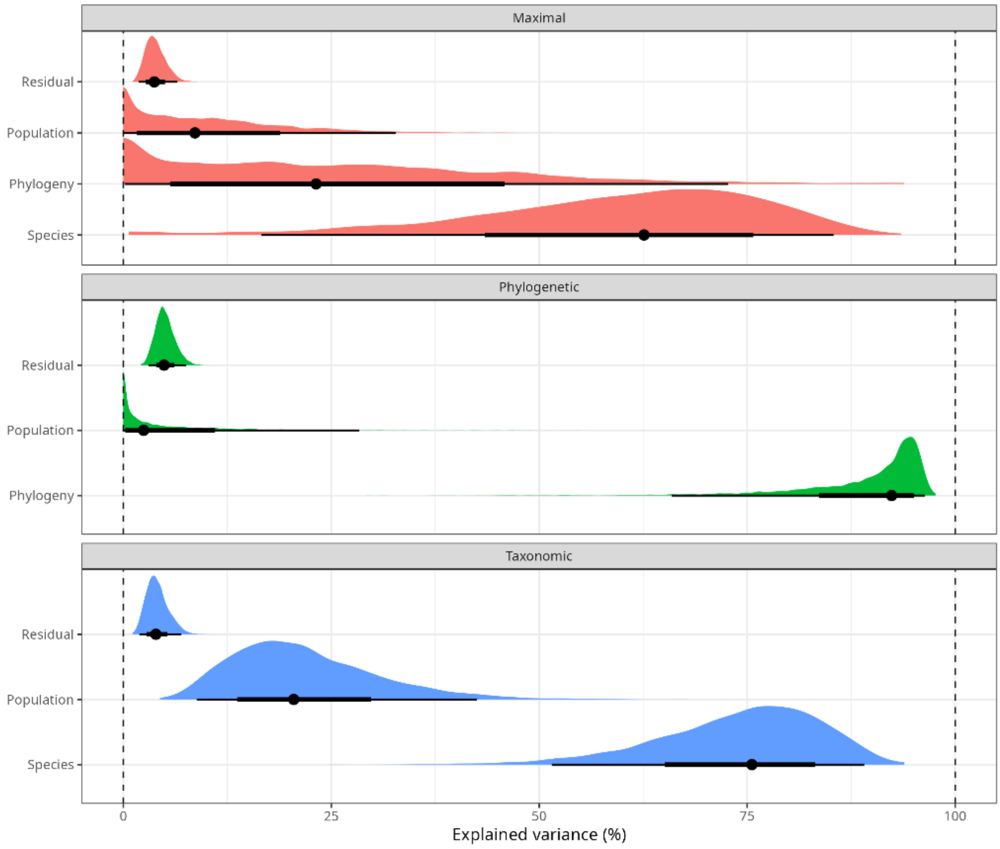

Figure S4. Plots showing the posterior distribution of explained variance in IGH partitioned by population (unstructured), phylogeny (phylogenetically structured population effect), species, and residual variation, for three models including alternative combinations of these terms.

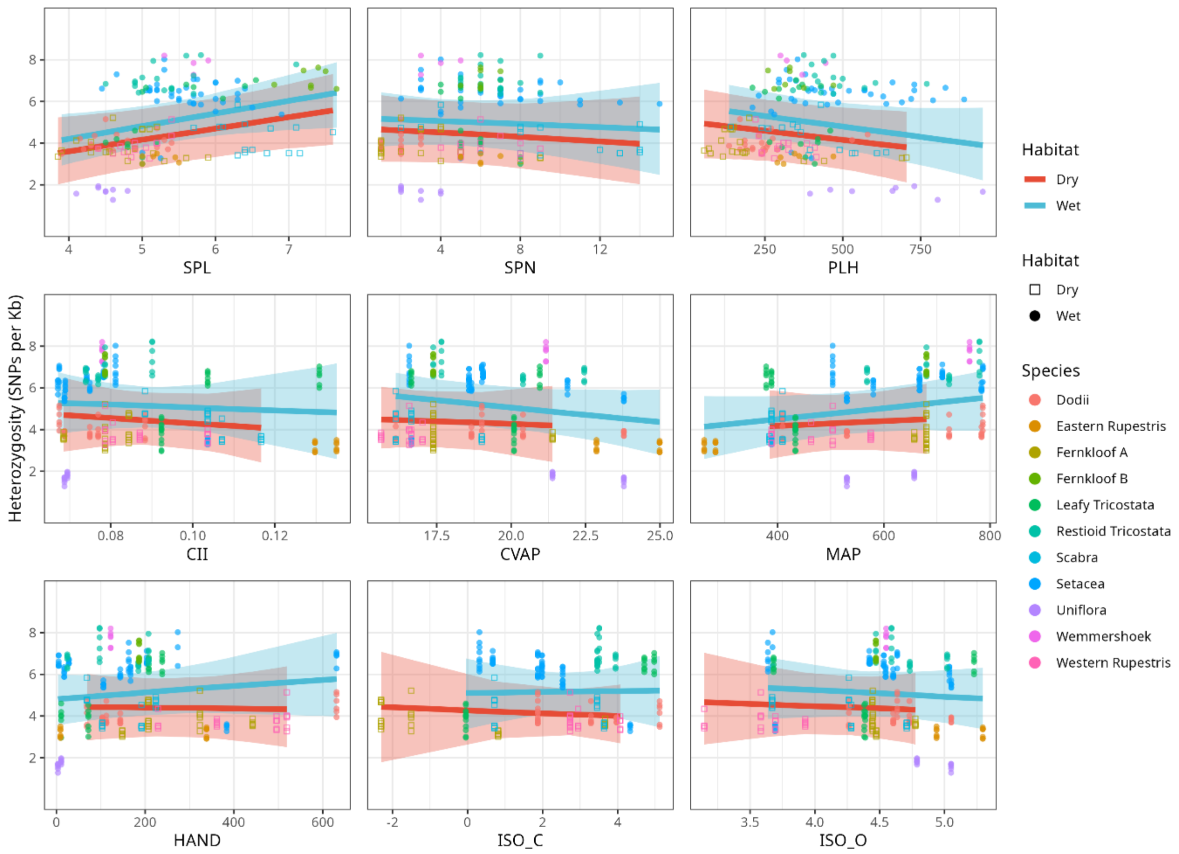

Figure S5. Relationship between genetic diversity and each covariate (a to h), for dry (red, squares) and wet (blue, circles) habitat species using univariate regression with species as a random effect (species LME models). Point colour is species-specific.

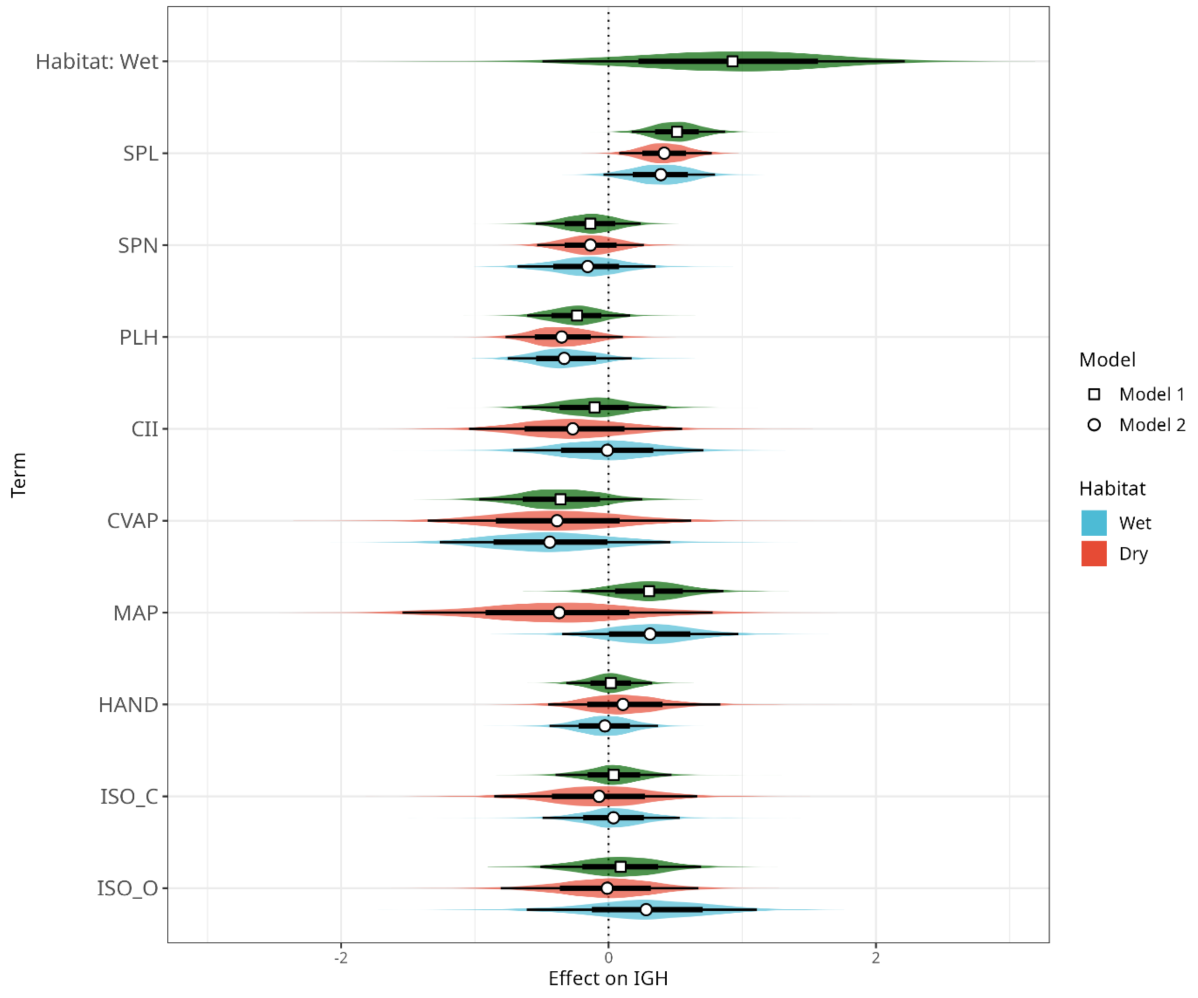

Figure S6. Posterior density distributions of slope estimates for multiple regression for the Species LME models 1 (green, squares) and 2 (blue and red, circles). With Dry as the reference category for habitat in Model 1, the first coefficient Habitat:Wet is the difference between wet and dry-habitat populations. Model 2 included habitat as an interaction term, thus, all slopes for model 2 are by habitat (blue = wet, red = dry). Points represent the mode, and thicker and thinner line ranges represent the 66% and 95% highest density intervals, respectively. Estimates correspond to the effect of a one standard deviation increase in the predictor variable on IGH (in SNPs per Kb).
