# Supplementary material for: Drivers of Population Genetic Diversity Across Ecologically Distinct Species in a Cape Fynbos Grass Clade
Source: Mol Ecol. 2026 Apr 12;35(8):e70342. doi: 10.1111/mec.70342 (PMC13071368; doi:10.1111/mec.70342)
Supplement: Supplementary file 2 — Data S1: Model‐heterozygosity [file MEC-35-e70342-s002.pdf]

model-heterozygosity

seth musker

Setup

```
pacman::p_load(
  tidyverse,
  ape,
  brms,
  tidybayes,
  modelr,
  patchwork,
  marginaleffects
)
dir.create(".brms",showWarnings = FALSE)

palette_wetdry<-c(Dry="#e64b35",Wet="#4dbbd5")
```

Load data and trees

```
# data ----
dat_indwise<-read.csv("data/all_data_combined_indwise_raw.csv")
dat_indwise$phylo<-dat_indwise$Pop

# Add isolation data from estimate-neighbour-isolation.qmd
isolation_data<-read_csv("data/Geographic_isolation_from_closest_relative.csv")
```

```
Rows: 43 Columns: 6
-- Column specification --------------------------------------------------------
Delimiter: ","
chr (2): Pop, Closest_relative
```

```
dbl (4): Node_distance, Patristic_distance, Distance, Distance_km

i Use `spec()` to retrieve the full column specification for this data.
i Specify the column types or set `show_col_types = FALSE` to quiet this message.
```

```r
isolation_data_conspecific<-read_csv("data/Geographic_isolation_from_nearest_conspecific.csv"
```

```
Rows: 37 Columns: 6
-- Column specification --------------------------------------------------------
Delimiter: ","
chr (3): Species, Pop, Nearest_conspecific_neighbour
dbl (3): Conspecific_neighbour_distance_km, Conspecific_distance_km_mean, Co...

i Use `spec()` to retrieve the full column specification for this data.
i Specify the column types or set `show_col_types = FALSE` to quiet this message.
```

```r
isolation_data_point_of_origin <- read_csv("data/Geographic_isolation_from_point_of_origin.cs
```

```
Rows: 43 Columns: 4
-- Column specification --------------------------------------------------------
Delimiter: ","
chr (2): Pop, geometry
dbl (2): distance_from_origin, distance_from_origin_km

i Use `spec()` to retrieve the full column specification for this data.
i Specify the column types or set `show_col_types = FALSE` to quiet this message.
```

```r
dat_indwise<-dat_indwise %>%
  left_join(isolation_data %>% select(-Distance)) %>%
  left_join(isolation_data_conspecific %>% select(-Species)) %>%
  left_join(isolation_data_point_of_origin %>% select(-geometry)) %>%
  as.data.frame()
```

```
Joining with `by = join_by(Pop)`
Joining with `by = join_by(Pop)`
Joining with `by = join_by(Pop)`
```

```
# trees ----
indtree<-read.tree("data/RUPSET_iqtree_allsamples.wLogDate.ingroup.cleaned.tre")
Ci <- vcv.phylo(indtree, corr = T)
tree<-read.tree("data/RUPSET_iqtree_allsamples.wLogDate.ingroup.poplevel.rescaled.cleaned.tre
Cp <- vcv.phylo(tree, corr = T)

# Reorder (not strictly necessary)
rownames(dat_indwise)<-dat_indwise$IndOG
dat_indwise<-dat_indwise[indtree$tip.label,]

indtree_PopLabels<-indtree
indtree_PopLabels$tip.label <- dat_indwise$phylo
```

Modeling details

CLIMATE

1. Long term overall climate variability

   CSI (climate stability index)

2. Short term climate stability (RAINFALL)

   Total annual rainfall CV 2006-2020 (MSWEP)

3. Water input

   Mean Annual Precipitation 2006-2020 (MSWEP)

Local conditions

1. Height above nearest drainage.

TRAITS

1. Pollen dispersal

   Plant height

2. Number of offspring

   Spikelet number

3. Investment in offspring

   Spikelet length

Species-level properties

1. Habitat (Wet vs. Dry)

Isolation

1. Geographic distance from most centre of origin
2. Geographic distance from nearest conspecific population

Prepare data

Re-express heterozygosity as number of variable sites per Kb (1,000 base pairs).

```
X_vars <- dat_indwise %>%
  select(
    Climate_variability = CSI,
    Rainfall_CV = MSWEP_cvAP,
    Rainfall_MAP = MSWEP_MAP,
    Height_above_drainage = hand,
    Plant_height,
    Spikelet_no,
    Spikelet_length,
    Dispersal_distance = Distance_km,
    Conspecific_isolation = Conspecific_neighbour_distance_km,
    Origin_isolation = distance_from_origin_km
  ) %>%
  mutate(
    Conspecific_isolation = case_when(
      Conspecific_isolation == 0 ~ Conspecific_isolation + 0.1, # add 100m
      .default = Conspecific_isolation
    )
  ) %>%
  mutate(Conspecific_isolation_log = log(Conspecific_isolation),
         Origin_isolation_log = log(Origin_isolation))

# Remove unwanted X variables
```

```
X_vars <- X_vars %>%
    select(-Dispersal_distance,-Conspecific_isolation,-Origin_isolation)

# Scale and center traits, env, and distance variables
X_vars_scaled <- as.data.frame(scale(X_vars))

X_vars_scaled_sds<-apply(X_vars_scaled,2,sd,na.rm=T)

cor.test(X_vars$Spikelet_no,X_vars$Spikelet_length)
```

```

    Pearson's product-moment correlation

data:  X_vars$Spikelet_no and X_vars$Spikelet_length
t = 7.5822, df = 162, p-value = 2.491e-12
alternative hypothesis: true correlation is not equal to 0
95 percent confidence interval:
 0.3890487 0.6166690
sample estimates:
      cor
0.5117851
```

```
cor.test(X_vars_scaled$Spikelet_no,X_vars_scaled$Spikelet_length)
```

```

    Pearson's product-moment correlation

data:  X_vars_scaled$Spikelet_no and X_vars_scaled$Spikelet_length
t = 7.5822, df = 162, p-value = 2.491e-12
alternative hypothesis: true correlation is not equal to 0
95 percent confidence interval:
 0.3890487 0.6166690
sample estimates:
      cor
0.5117851
```

```
Cat_vars<-dat_indwise %>%
  select(Habitat) %>%
  mutate(Habitat=as.factor(Habitat))
```

```r
Y_var<-dat_indwise %>%
  mutate(Heterozygosity.pKb=Heterozygosity*1e3) %>%
  select(Heterozygosity.pKb)
sd(Y_var$Heterozygosity.pKb)
```

```
[1] 1.645874
```

```r
Grp_vars<-dat_indwise %>%
  select(Ind=IndOG,
         Pop,
         phylo,
         Species = New.Treatment)

dat_modeling<-bind_cols(
  Y_var,
  X_vars_scaled,
  Cat_vars,
  Grp_vars
) %>%
  as_tibble() %>%
  mutate(miss=is.na(Spikelet_length))
dat_modeling
```

```
# A tibble: 257 x 16
   Heterozygosity.pKb Climate_variability Rainfall_CV Rainfall_MAP
                <dbl>               <dbl>       <dbl>        <dbl>
 1               8.02              -0.257     -1.01         -0.455
 2               7.30              -0.257     -1.01         -0.455
 3               6.40              -0.257     -1.01         -0.455
 4               5.95              -0.611     -0.175         1.39
 5               5.85              -0.611     -0.175         1.39
 6               5.90              -0.611     -0.175         1.39
 7               6.02              -0.611     -0.175         1.39
 8               5.91              -0.611     -0.175         1.39
 9               6.24              -0.611     -0.175         1.39
10               6.99              -1.03      -0.0341        1.40
# i 247 more rows
# i 12 more variables: Height_above_drainage <dbl>, Plant_height <dbl>,
#   Spikelet_no <dbl>, Spikelet_length <dbl>, Conspecific_isolation_log <dbl>,
#   Origin_isolation_log <dbl>, Habitat <fct>, Ind <chr>, Pop <chr>,
#   phylo <chr>, Species <chr>, miss <lgl>
```

```
dat_modeling %>%
  group_by(Pop) %>%
  slice_head(n=1) %>%
  ungroup() %>%
  count(is.na(Conspecific_isolation_log))
```

```
# A tibble: 2 x 2
  `is.na(Conspecific_isolation_log)`     n
  <lgl>                              <int>
1 FALSE                                 37
2 TRUE                                   6
```

```
dat_modeling %>%
  count(is.na(Spikelet_length))
```

```
# A tibble: 2 x 2
  `is.na(Spikelet_length)`     n
  <lgl>                    <int>
1 FALSE                      164
2 TRUE                        93
```

Modeling: Phylogenetic LME

Model 1 = All vars

UPDATE: August 27, 2025 Dispersal distance is being removed.

```
#|echo: false
model_1_brmsformula <- bf(
  Heterozygosity.pKb ~
    Origin_isolation_log +
    Habitat +
    Climate_variability +
    Rainfall_CV +
    Rainfall_MAP +
    # Dispersal_distance +
    mi(Conspecific_isolation_log) +
    Height_above_drainage +
    mi(Plant_height) +
```

```
    mi(Spikelet_no) +
    mi(Spikelet_length) +
    (1 | gr(phylo, cov = Cp)) +
    (1 | Pop)
) +
  bf(
    Plant_height | mi() ~ 1 + Species + (1 | Pop) + (1 | gr(Ind, cov = Ci))
  ) +
  bf(
    Spikelet_no | mi() ~ 1 + Species + (1 | Pop) + (1 | gr(Ind, cov = Ci))
  ) +
  bf(
    Spikelet_length | mi() ~ 1 + Species + (1 | Pop) + (1 | gr(Ind, cov = Ci))
  ) +
  bf(Conspecific_isolation_log | mi() ~ 1) +
  set_rescor(TRUE)

model_1 <- brm(
  model_1_brmsformula,
  prior=c(
    # SD priors
    prior(std_normal(),class=sd,group=phylo,resp=HeterozygositypKb,lb=0),
    prior(std_normal(),class=sd,group=Pop,resp=HeterozygositypKb,lb=0),
    prior(std_normal(),class=sd,group=Pop,resp=Plantheight,lb=0),
    prior(std_normal(),class=sd,group=Pop,resp=Spikeletno,lb=0),
    prior(std_normal(),class=sd,group=Pop,resp=Spikeletlength,lb=0),
    prior(std_normal(),class=sd,group=Ind,resp=Plantheight,lb=0),
    prior(std_normal(),class=sd,group=Ind,resp=Spikeletno,lb=0),
    prior(std_normal(),class=sd,group=Ind,resp=Spikeletlength,lb=0),
    # beta priors
    prior(std_normal(),class=b,resp=HeterozygositypKb),
    # residual corr priors
    prior(lkj_corr_cholesky(2),class=Lrescor)
  ),
  data = dat_modeling,
  family = gaussian(),
  data2 = list(Cp = Cp, Ci = Ci),
  backend = "cmdstanr",
  save_pars = save_pars(all = TRUE),
  iter = 2000,
  cores = 4,
  chains = 4,
```

```r
  refresh = 250,
  control = list(adapt_delta = 0.995, max_treedepth = 12),
  save_model = ".brms/model_1_rescor_COO.stan",
  file = ".brms/model_1_rescor_COO",
  seed = 42
)
model_1
```

```
Loading required namespace: rstan

 Family: MV(gaussian, gaussian, gaussian, gaussian, gaussian)
  Links: mu = identity
         mu = identity
         mu = identity
         mu = identity
         mu = identity
Formula: Heterozygosity.pKb ~ Origin_isolation_log + Habitat + Climate_variability + Rainfal
         Plant_height | mi() ~ 1 + Species + (1 | Pop) + (1 | gr(Ind, cov = Ci))
         Spikelet_no | mi() ~ 1 + Species + (1 | Pop) + (1 | gr(Ind, cov = Ci))
         Spikelet_length | mi() ~ 1 + Species + (1 | Pop) + (1 | gr(Ind, cov = Ci))
         Conspecific_isolation_log | mi() ~ 1
   Data: dat_modeling (Number of observations: 257)
  Draws: 4 chains, each with iter = 2000; warmup = 1000; thin = 1;
         total post-warmup draws = 4000

Multilevel Hyperparameters:
~phylo (Number of levels: 43)
                                Estimate Est.Error l-95% CI u-95% CI Rhat
sd(HeterozygositypKb_Intercept)     1.32      0.23     0.84     1.75 1.01
                                Bulk_ESS Tail_ESS
sd(HeterozygositypKb_Intercept)      677      568

~Pop (Number of levels: 43)
                                Estimate Est.Error l-95% CI u-95% CI Rhat
sd(HeterozygositypKb_Intercept)     0.35      0.25     0.01     0.92 1.01
sd(Plantheight_Intercept)           0.62      0.15     0.28     0.89 1.01
sd(Spikeletno_Intercept)            0.55      0.17     0.17     0.84 1.02
sd(Spikeletlength_Intercept)        0.24      0.13     0.02     0.50 1.02
                                Bulk_ESS Tail_ESS
sd(HeterozygositypKb_Intercept)      356      571
sd(Plantheight_Intercept)            393      427
```

```
sd(Spikeletno_Intercept)            314      333
sd(Spikeletlength_Intercept)        232      498

~Ind (Number of levels: 257)
                             Estimate Est.Error l-95% CI u-95% CI Rhat Bulk_ESS
sd(Plantheight_Intercept)        0.49      0.29     0.02     1.05 1.01      195
sd(Spikeletno_Intercept)         0.45      0.23     0.03     0.88 1.04      228
sd(Spikeletlength_Intercept)     0.64      0.18     0.08     0.92 1.04      138
                             Tail_ESS
sd(Plantheight_Intercept)         365
sd(Spikeletno_Intercept)          462
sd(Spikeletlength_Intercept)       67

Regression Coefficients:
                                            Estimate Est.Error l-95% CI
HeterozygositypKb_Intercept                     4.17      0.59     3.01
Plantheight_Intercept                          -0.73      0.52    -1.77
Spikeletno_Intercept                           -1.25      0.45    -2.14
Spikeletlength_Intercept                       -0.85      0.53    -1.90
Conspecificisolationlog_Intercept               0.00      0.07    -0.14
HeterozygositypKb_Origin_isolation_log         -0.09      0.32    -0.71
HeterozygositypKb_HabitatWet                    0.94      0.61    -0.26
HeterozygositypKb_Climate_variability          -0.11      0.29    -0.65
HeterozygositypKb_Rainfall_CV                  -0.15      0.32    -0.79
HeterozygositypKb_Rainfall_MAP                  0.40      0.28    -0.14
HeterozygositypKb_Height_above_drainage         0.02      0.18    -0.33
Plantheight_SpeciesEasternRupestris             0.62      0.86    -1.12
Plantheight_SpeciesFernkloofA                  -0.11      0.77    -1.65
Plantheight_SpeciesFernkloofB                   0.45      0.99    -1.50
Plantheight_SpeciesLeafyTricostata              0.53      0.62    -0.73
Plantheight_SpeciesRestioidTricostata           1.23      0.79    -0.36
Plantheight_SpeciesScabra                       0.86      0.76    -0.67
Plantheight_SpeciesSetacea                      1.21      0.60    -0.02
Plantheight_SpeciesUniflora                     2.25      0.88     0.48
Plantheight_SpeciesWemmershoek                  0.61      0.93    -1.22
Plantheight_SpeciesWesternRupestris             0.31      0.75    -1.19
Spikeletno_SpeciesEasternRupestris              1.34      0.77    -0.26
Spikeletno_SpeciesFernkloofA                    0.63      0.62    -0.58
Spikeletno_SpeciesFernkloofB                    1.37      0.86    -0.35
Spikeletno_SpeciesLeafyTricostata               1.30      0.56     0.18
Spikeletno_SpeciesRestioidTricostata            1.52      0.69     0.21
Spikeletno_SpeciesScabra                        2.29      0.69     0.90
Spikeletno_SpeciesSetacea                       1.49      0.53     0.41
```

Spikeletno_SpeciesUniflora	0.33	0.78	-1.23
Spikeletno_SpeciesWemmershoek	0.67	0.84	-0.95
Spikeletno_SpeciesWesternRupestris	1.46	0.67	0.12
Spikeletlength_SpeciesEasternRupestris	0.59	0.82	-1.03
Spikeletlength_SpeciesFernkloofA	0.12	0.68	-1.22
Spikeletlength_SpeciesFernkloofB	3.15	0.86	1.44
Spikeletlength_SpeciesLeafyTricostata	0.83	0.55	-0.25
Spikeletlength_SpeciesRestioidTricostata	0.67	0.79	-0.93
Spikeletlength_SpeciesScabra	2.14	0.74	0.67
Spikeletlength_SpeciesSetacea	1.04	0.56	-0.10
Spikeletlength_SpeciesUniflora	-0.28	0.82	-2.00
Spikeletlength_SpeciesWemmershoek	1.19	0.72	-0.20
Spikeletlength_SpeciesWesternRupestris	0.30	0.75	-1.19
HeterozygositypKb_miConspecific_isolation_log	-0.04	0.23	-0.48
HeterozygositypKb_miPlant_height	-0.14	0.22	-0.57
HeterozygositypKb_miSpikelet_no	0.05	0.22	-0.39
HeterozygositypKb_miSpikelet_length	0.63	0.21	0.23

	u-95% CI	Rhat	Bulk_ESS	Tail_ESS
HeterozygositypKb_Intercept	5.35	1.00	1457	1955
Plantheight_Intercept	0.33	1.00	716	854
Spikeletno_Intercept	-0.36	1.00	919	1154
Spikeletlength_Intercept	0.20	1.00	848	933
Conspecificisolationlog_Intercept	0.14	1.00	5266	2021
HeterozygositypKb_Origin_isolation_log	0.53	1.00	1194	2131
HeterozygositypKb_HabitatWet	2.13	1.00	1330	2061
HeterozygositypKb_Climate_variability	0.47	1.00	1216	2187
HeterozygositypKb_Rainfall_CV	0.49	1.00	1069	1674
HeterozygositypKb_Rainfall_MAP	0.96	1.00	1263	1912
HeterozygositypKb_Height_above_drainage	0.37	1.00	1399	2204
Plantheight_SpeciesEasternRupestris	2.37	1.00	1288	1448
Plantheight_SpeciesFernkloofA	1.35	1.01	594	1101
Plantheight_SpeciesFernkloofB	2.37	1.00	1323	1549
Plantheight_SpeciesLeafyTricostata	1.72	1.00	1238	1787
Plantheight_SpeciesRestioidTricostata	2.81	1.00	1060	1131
Plantheight_SpeciesScabra	2.32	1.00	925	1152
Plantheight_SpeciesSetacea	2.40	1.00	954	1109
Plantheight_SpeciesUniflora	3.94	1.00	1016	1287
Plantheight_SpeciesWemmershoek	2.43	1.00	1904	2163
Plantheight_SpeciesWesternRupestris	1.79	1.00	1050	1155
Spikeletno_SpeciesEasternRupestris	2.86	1.00	1274	1585
Spikeletno_SpeciesFernkloofA	1.85	1.00	1014	1193
Spikeletno_SpeciesFernkloofB	3.14	1.00	1603	2243
Spikeletno_SpeciesLeafyTricostata	2.40	1.00	1229	1483

```
Spikeletno_SpeciesRestioidTricostata              2.93 1.00     1171     1346
Spikeletno_SpeciesScabra                          3.64 1.00     1199     1460
Spikeletno_SpeciesSetacea                         2.49 1.00     1146     1478
Spikeletno_SpeciesUniflora                        1.88 1.00     1288     1467
Spikeletno_SpeciesWemmershoek                     2.35 1.00     2222     2522
Spikeletno_SpeciesWesternRupestris                2.76 1.00     1340     1978
Spikeletlength_SpeciesEasternRupestris            2.24 1.00     1347     1247
Spikeletlength_SpeciesFernkloofA                  1.44 1.00      910     1208
Spikeletlength_SpeciesFernkloofB                  4.84 1.00     1502     2056
Spikeletlength_SpeciesLeafyTricostata             1.91 1.00     1417     2206
Spikeletlength_SpeciesRestioidTricostata          2.24 1.00     1104     1379
Spikeletlength_SpeciesScabra                      3.63 1.00     1063     1557
Spikeletlength_SpeciesSetacea                     2.10 1.00     1339     2086
Spikeletlength_SpeciesUniflora                    1.28 1.00     1228     1806
Spikeletlength_SpeciesWemmershoek                 2.59 1.00     1629     2596
Spikeletlength_SpeciesWesternRupestris            1.79 1.01     1024     1332
HeterozygositypKb_miConspecific_isolation_log     0.42 1.00     1076     1530
HeterozygositypKb_miPlant_height                  0.30 1.01      352      860
HeterozygositypKb_miSpikelet_no                   0.47 1.00      648     1132
HeterozygositypKb_miSpikelet_length               1.04 1.00      531     1461

Further Distributional Parameters:
                              Estimate Est.Error l-95% CI u-95% CI Rhat
sigma_HeterozygositypKb           0.46      0.08     0.35     0.67 1.00
sigma_Plantheight                 0.54      0.05     0.45     0.66 1.00
sigma_Spikeletno                  0.45      0.04     0.38     0.53 1.01
sigma_Spikeletlength              0.40      0.04     0.33     0.47 1.01
sigma_Conspecificisolationlog     1.01      0.05     0.92     1.11 1.00
                              Bulk_ESS Tail_ESS
sigma_HeterozygositypKb           1121     1530
sigma_Plantheight                  852     1478
sigma_Spikeletno                   790     1906
sigma_Spikeletlength               433      693
sigma_Conspecificisolationlog     4681     2988

Residual Correlations:
                                                  Estimate Est.Error l-95% CI
rescor(HeterozygositypKb,Plantheight)                 0.02      0.28    -0.52
rescor(HeterozygositypKb,Spikeletno)                 -0.02      0.24    -0.49
rescor(Plantheight,Spikeletno)                        0.19      0.12    -0.05
rescor(HeterozygositypKb,Spikeletlength)             -0.40      0.21    -0.74
rescor(Plantheight,Spikeletlength)                    0.16      0.12    -0.08
rescor(Spikeletno,Spikeletlength)                     0.06      0.11    -0.16
```

```
rescor(HeterozygositypKb,Conspecificisolationlog)    -0.00      0.37    -0.69
rescor(Plantheight,Conspecificisolationlog)           0.12      0.39    -0.61
rescor(Spikeletno,Conspecificisolationlog)            0.14      0.20    -0.26
rescor(Spikeletlength,Conspecificisolationlog)        0.13      0.19    -0.25
                                                  u-95% CI Rhat Bulk_ESS
rescor(HeterozygositypKb,Plantheight)                 0.56 1.01      456
rescor(HeterozygositypKb,Spikeletno)                  0.43 1.00      688
rescor(Plantheight,Spikeletno)                        0.41 1.01     1127
rescor(HeterozygositypKb,Spikeletlength)              0.06 1.00      525
rescor(Plantheight,Spikeletlength)                    0.38 1.00     1196
rescor(Spikeletno,Spikeletlength)                     0.28 1.00     1820
rescor(HeterozygositypKb,Conspecificisolationlog)     0.68 1.00     1192
rescor(Plantheight,Conspecificisolationlog)           0.72 1.03      203
rescor(Spikeletno,Conspecificisolationlog)            0.50 1.02      492
rescor(Spikeletlength,Conspecificisolationlog)        0.47 1.00     1567
                                                  Tail_ESS
rescor(HeterozygositypKb,Plantheight)                  930
rescor(HeterozygositypKb,Spikeletno)                  1341
rescor(Plantheight,Spikeletno)                        2336
rescor(HeterozygositypKb,Spikeletlength)              1449
rescor(Plantheight,Spikeletlength)                    2470
rescor(Spikeletno,Spikeletlength)                     2105
rescor(HeterozygositypKb,Conspecificisolationlog)     1539
rescor(Plantheight,Conspecificisolationlog)            510
rescor(Spikeletno,Conspecificisolationlog)            1685
rescor(Spikeletlength,Conspecificisolationlog)        2600

Draws were sampled using sample(hmc). For each parameter, Bulk_ESS
and Tail_ESS are effective sample size measures, and Rhat is the potential
scale reduction factor on split chains (at convergence, Rhat = 1).
```

```r
sink("results/model_1_rescor_C00_brmssummary.txt")
print(model_1,digits=3)
sink()

mcmc_plot(model_1)
```

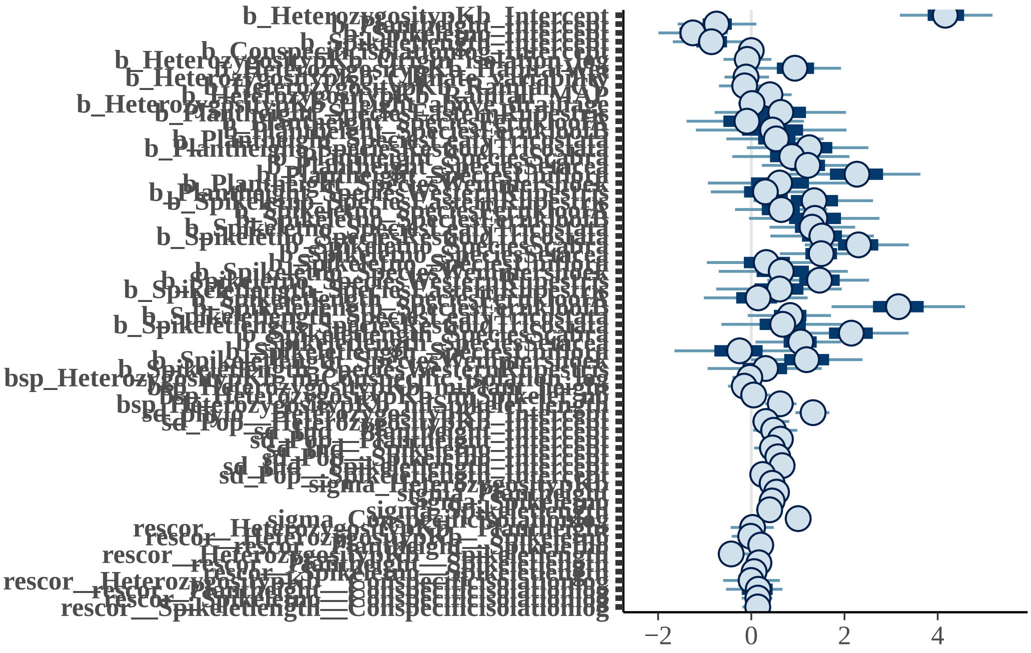


```
# View(model_1$prior)
```

pp checks

```
pp_check(model_1, resp = "HeterozygositypKb", ndraws = 100)
pp_check(model_1, resp = "Plantheight", ndraws = 100)
pp_check(model_1, resp = "Spikeletlength", ndraws = 100)
pp_check(model_1, resp = "Spikeletno", ndraws = 100)
pp_check(model_1, resp = "Conspecificisolationlog", ndraws = 100)
pp_check(model_1, resp = "Conspecificisolationlog", ndraws = 100,
         type = "ecdf_overlay")
pp_check(model_1, resp = "Conspecificisolationlog", ndraws = 100,
         type = "error_scatter_avg")
pp_check(model_1, resp = "Conspecificisolationlog", ndraws = 100,
         type = "scatter_avg")

pp_check(model_1, resp = "Spikeletlength", ndraws = 100,
         type = "error_scatter_avg")
pp_check(model_1, resp = "Spikeletlength", ndraws = 100,
         type = "scatter_avg")
```

Note to self: don't use subsetting or indexing. As is, it already uses the observed values. See the stancode.

Post-process model 1

```
my_vars<-c(
    "Habitat",
    "Rainfall_MAP",
    "Rainfall_CV",
    "Climate_variability",
    "Spikelet_length",
    "Spikelet_no",
    "Plant_height",
    "Height_above_drainage",
    # "Dispersal_distance",
    "Conspecific_isolation_log",
    "Origin_isolation_log"
    )
# model_1_conditional_effects <- conditional_effects(
#   model_1,
#   effects = my_vars,
#   plot = FALSE,
#   resp="HeterozygositypKb")
# # # View(model_1_conditional_effects)
# plot(model_1_conditional_effects,
#      ask=F)

# Note we use avg_slopes (not just slopes()) because we aren't using "by=[variable]" (like be
model_1_slopes<-avg_slopes(
  model_1,
  variables = my_vars,
  re_formula=NA,
  resp="HeterozygositypKb"
)
model_1_slopes_draws <- model_1_slopes |>
  posterior_draws()
gc()
```

```
           used   (Mb) gc trigger   (Mb)  max used   (Mb)
Ncells  4084563 218.2    6103774 326.0   6103774 326.0
Vcells 17745759 135.4   75342956 574.9 117723367 898.2
```

```
## Estimate lambda!
model_1 %>% as_tibble() %>%
  dplyr::select(sigma_b = sd_phylo__HeterozygositypKb_Intercept,
```

```
                 sigma_p = sd_Pop__HeterozygositypKb_Intercept,
                 sigma_e = sigma_HeterozygositypKb) %>%
  mutate(h2 = sigma_b^2/(sigma_b^2 + sigma_p^2 + sigma_e^2),
         hpop = sigma_p^2/(sigma_b^2 + sigma_p^2 + sigma_e^2)) %>%
  select(c("h2","hpop")) %>%
  map(.,\(x)print(median_qi(x,.width=c(.89,.95))))
```

```
          y      ymin      ymax .width .point .interval
1 0.8432707 0.5504176 0.9309083   0.89 median        qi
2 0.8432707 0.4320557 0.9420143   0.95 median        qi
          y         ymin      ymax .width .point .interval
1 0.0443039 3.868794e-04 0.3219276   0.89 median        qi
2 0.0443039 9.578358e-05 0.4362062   0.95 median        qi


$h2
          y      ymin      ymax .width .point .interval
1 0.8432707 0.5504176 0.9309083   0.89 median        qi
2 0.8432707 0.4320557 0.9420143   0.95 median        qi

$hpop
          y         ymin      ymax .width .point .interval
1 0.0443039 3.868794e-04 0.3219276   0.89 median        qi
2 0.0443039 9.578358e-05 0.4362062   0.95 median        qi
```

```
marginal_R2_model_1<-bayes_R2(model_1,
                               resp="HeterozygositypKb",
                               re_formula=NA)
marginal_R2_model_1
```

```
                    Estimate  Est.Error      Q2.5     Q97.5
R2HeterozygositypKb 0.373956 0.09424285 0.1863401 0.5462016
```

Check that conspecific isolation has been appropriately imputed.

```
pp_check(model_1,resp="Conspecificisolationlog",ndraws = 100)
```

```
Warning: NA responses are not shown in 'pp_check'.
```

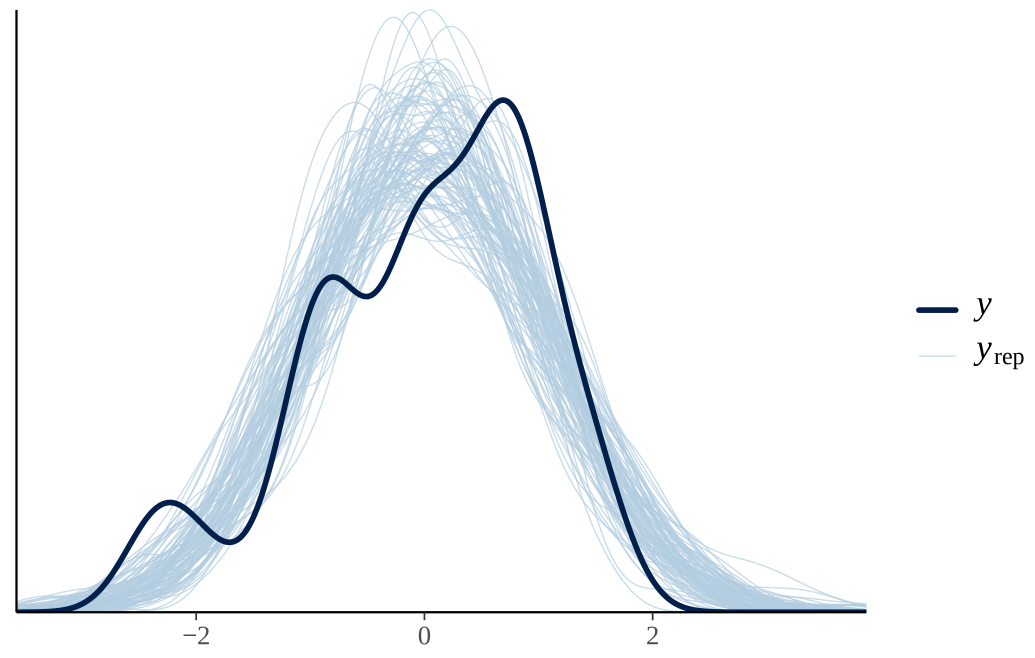


```
## plot model 1 slopes
### Plot slopes ----
to_levs<-c(
    "Habitat: Wet - Dry",

    "Rainfall_MAP",

    "Height_above_drainage",

    "Rainfall_CV",

    "Climate_variability",

    "Spikelet_length",
    "Spikelet_no",
    "Plant_height",

    # "Dispersal_distance",
    "Conspecific_isolation_log",
    "Origin_isolation_log"

    )
```

```r
base<-model_1_slopes_draws %>%
  mutate(term=gsub("Habitat","Habitat: Wet - Dry",term)) %>%
  mutate(term=fct_relevel(term,rev(to_levs))) %>%
  ggplot(aes(x=draw,y=term))+
  labs(x="Effect on IGH",y="Term")+
  theme_bw()+
  guides(fill=guide_legend(override.aes = list(size=NA)))

model_1_slopes_plot<-base+
  stat_eye(slab_alpha=.5,
           shape=21,
           fill="darkgreen",
           normalize="xy",
           # position = "dodgejust",
           height=.75,
           point_interval = mode_hdi,
           .width = c(0.66, 0.95)
  )+
  geom_vline(xintercept = 0,linetype=3)+
  scale_x_continuous(breaks = scales::extended_breaks(10),
                     limits=c(-3.2,3.2))

expected_slope_directions <- data.frame(
  term = c(
    "Habitat: Wet - Dry",

    "Rainfall_MAP",

    "Height_above_drainage",

    "Rainfall_CV",

    "Climate_variability",

    "Spikelet_length",
    "Spikelet_no",
    "Plant_height",

    # "Dispersal_distance",
    "Conspecific_isolation_log",
    "Origin_isolation_log"
    ),
```

```
  direction = c(1, 1, -1, -1, -1, 1, 1, 1, -1, -1)
)
model_1_slopes_plot_arrows<-
  model_1_slopes_plot+
  annotate('segment',
           y=expected_slope_directions$term,
           x=0,
           xend=expected_slope_directions$direction,
           arrow = arrow(length = unit(2,"mm"),type = "closed"),
           colour="red",
           alpha=.5)

model_1_slopes_plot_arrows
```

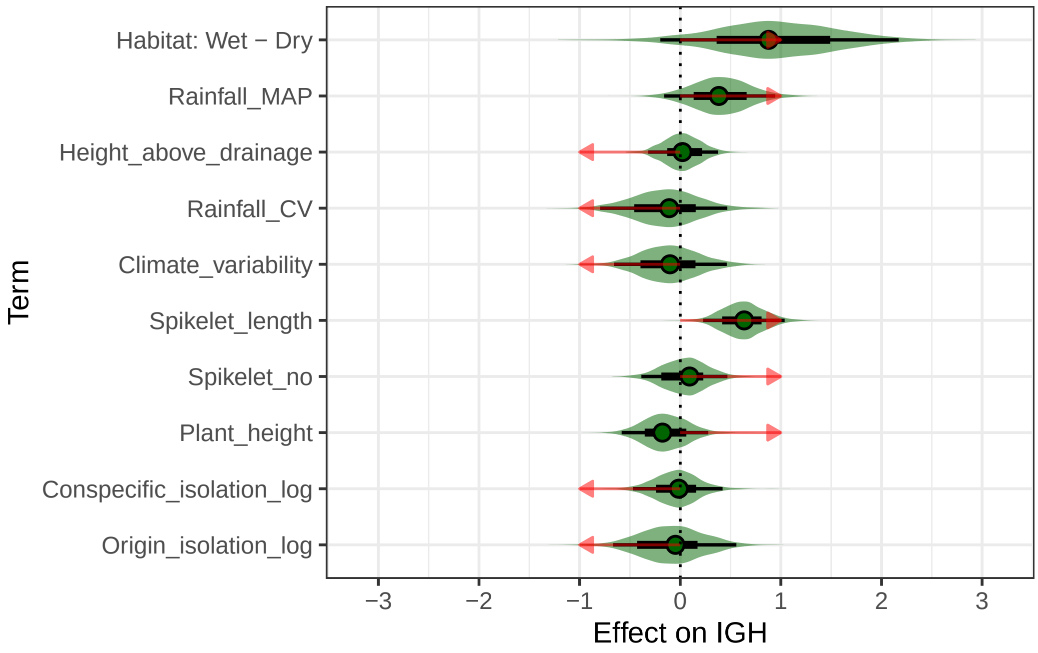


```
ggsave("plots/MarginalEffects_slabs_model_1_rescor_COO.pdf",
       plot=model_1_slopes_plot_arrows,
       width=24,height=20,units="cm")
ggsave("plots/MarginalEffects_slabs_model_1_rescor_COO.png",
       plot=model_1_slopes_plot_arrows,
       width=24,height=20,units="cm")
# system2("open","plots/MarginalEffects_slabs_model_1_rescor_COO.pdf")
```

Model 2: Including interactions with Habitat

We also allow separate intercepts, random effect SDs, and sigmas for each habitat.

```
#|echo: false
model_2_brmsformula<-bf(
  Heterozygosity.pKb ~ 0 +
    Habitat +
    (Climate_variability +
       Rainfall_CV +
       Rainfall_MAP +
       Origin_isolation_log +
       # Dispersal_distance +
       mi(Conspecific_isolation_log) +
       Height_above_drainage +
       mi(Plant_height) +
       mi(Spikelet_no) +
       mi(Spikelet_length)):Habitat +
    (1 | gr(phylo, cov = Cp, by = Habitat)) +
    (1 | gr(Pop, by = Habitat)),
  sigma ~ 0 + Habitat
) +
  bf(
    Plant_height | mi() ~ 1 + Species + (1|Pop) + (1 | gr(Ind, cov = Ci))
  ) +
  bf(
    Spikelet_no | mi() ~ 1 + Species + (1|Pop) + (1 | gr(Ind, cov = Ci))
    ) +
  bf(
    Spikelet_length | mi() ~ 1 + Species + (1|Pop) + (1 | gr(Ind, cov = Ci))
  ) +
  bf(Conspecific_isolation_log | mi() ~ 1) +
  set_rescor(TRUE)

get_prior(model_2_brmsformula,
          data=dat_modeling,data2=list(Cp=Cp,Ci=Ci))
```

```
                prior     class                                  coef group
               lkj(1)    rescor
 student_t(3, 0, 2.5) Intercept
 student_t(3, 0, 2.5)     sigma
               (flat)         b
```

(flat)	b	HabitatDry	
(flat)	b	HabitatDry:Climate_variability	
(flat)	b	HabitatDry:Height_above_drainage	
(flat)	b	HabitatDry:miConspecific_isolation_log	
(flat)	b	HabitatDry:miPlant_height	
(flat)	b	HabitatDry:miSpikelet_length	
(flat)	b	HabitatDry:miSpikelet_no	
(flat)	b	HabitatDry:Origin_isolation_log	
(flat)	b	HabitatDry:Rainfall_CV	
(flat)	b	HabitatDry:Rainfall_MAP	
(flat)	b	HabitatWet	
(flat)	b	HabitatWet:Climate_variability	
(flat)	b	HabitatWet:Height_above_drainage	
(flat)	b	HabitatWet:miConspecific_isolation_log	
(flat)	b	HabitatWet:miPlant_height	
(flat)	b	HabitatWet:miSpikelet_length	
(flat)	b	HabitatWet:miSpikelet_no	
(flat)	b	HabitatWet:Origin_isolation_log	
(flat)	b	HabitatWet:Rainfall_CV	
(flat)	b	HabitatWet:Rainfall_MAP	
student_t(3, 0, 2.5)	sd		
student_t(3, 0, 2.5)	sd		phylo
student_t(3, 0, 2.5)	sd	Intercept	phylo
student_t(3, 0, 2.5)	sd		Pop
student_t(3, 0, 2.5)	sd	Intercept	Pop
(flat)	b		
(flat)	b	HabitatDry	
(flat)	b	HabitatWet	
(flat)	b		
(flat)	b	SpeciesEasternRupestris	
(flat)	b	SpeciesFernkloofA	
(flat)	b	SpeciesFernkloofB	
(flat)	b	SpeciesLeafyTricostata	
(flat)	b	SpeciesRestioidTricostata	
(flat)	b	SpeciesScabra	
(flat)	b	SpeciesSetacea	
(flat)	b	SpeciesUniflora	
(flat)	b	SpeciesWemmershoek	
(flat)	b	SpeciesWesternRupestris	
student_t(3, 0, 2.5)	Intercept		
student_t(3, 0, 2.5)	sd		
student_t(3, 0, 2.5)	sd		Ind
student_t(3, 0, 2.5)	sd	Intercept	Ind

```
student_t(3, 0, 2.5)        sd                                          Pop
student_t(3, 0, 2.5)        sd                               Intercept   Pop
student_t(3, 0, 2.5)     sigma
              (flat)         b
              (flat)         b                 SpeciesEasternRupestris
              (flat)         b                       SpeciesFernkloofA
              (flat)         b                       SpeciesFernkloofB
              (flat)         b                 SpeciesLeafyTricostata
              (flat)         b               SpeciesRestioidTricostata
              (flat)         b                           SpeciesScabra
              (flat)         b                          SpeciesSetacea
              (flat)         b                         SpeciesUniflora
              (flat)         b                      SpeciesWemmershoek
              (flat)         b                SpeciesWesternRupestris
student_t(3, 0, 2.5) Intercept
student_t(3, 0, 2.5)        sd
student_t(3, 0, 2.5)        sd                                          Ind
student_t(3, 0, 2.5)        sd                               Intercept   Ind
student_t(3, 0, 2.5)        sd                                          Pop
student_t(3, 0, 2.5)        sd                               Intercept   Pop
student_t(3, 0, 2.5)     sigma
              (flat)         b
              (flat)         b                 SpeciesEasternRupestris
              (flat)         b                       SpeciesFernkloofA
              (flat)         b                       SpeciesFernkloofB
              (flat)         b                 SpeciesLeafyTricostata
              (flat)         b               SpeciesRestioidTricostata
              (flat)         b                           SpeciesScabra
              (flat)         b                          SpeciesSetacea
              (flat)         b                         SpeciesUniflora
              (flat)         b                      SpeciesWemmershoek
              (flat)         b                SpeciesWesternRupestris
student_t(3, 0, 2.5) Intercept
student_t(3, 0, 2.5)        sd
student_t(3, 0, 2.5)        sd                                          Ind
student_t(3, 0, 2.5)        sd                               Intercept   Ind
student_t(3, 0, 2.5)        sd                                          Pop
student_t(3, 0, 2.5)        sd                               Intercept   Pop
student_t(3, 0, 2.5)     sigma
                   resp  dpar nlpar lb ub tag       source
                                                   default
Conspecificisolationlog                            default
Conspecificisolationlog              0             default
```

HeterozygositypKb			default
HeterozygositypKb			(vectorized)
HeterozygositypKb			(vectorized)
HeterozygositypKb			(vectorized)
HeterozygositypKb			(vectorized)
HeterozygositypKb			(vectorized)
HeterozygositypKb			(vectorized)
HeterozygositypKb			(vectorized)
HeterozygositypKb			(vectorized)
HeterozygositypKb			(vectorized)
HeterozygositypKb			(vectorized)
HeterozygositypKb			(vectorized)
HeterozygositypKb			(vectorized)
HeterozygositypKb			(vectorized)
HeterozygositypKb			(vectorized)
HeterozygositypKb			(vectorized)
HeterozygositypKb			(vectorized)
HeterozygositypKb			(vectorized)
HeterozygositypKb			(vectorized)
HeterozygositypKb			(vectorized)
HeterozygositypKb			(vectorized)
HeterozygositypKb		0	default
HeterozygositypKb		0	(vectorized)
HeterozygositypKb		0	(vectorized)
HeterozygositypKb		0	(vectorized)
HeterozygositypKb		0	(vectorized)
HeterozygositypKb	sigma		default
HeterozygositypKb	sigma		(vectorized)
HeterozygositypKb	sigma		(vectorized)
Plantheight			default
Plantheight			(vectorized)
Plantheight			(vectorized)
Plantheight			(vectorized)
Plantheight			(vectorized)
Plantheight			(vectorized)
Plantheight			(vectorized)
Plantheight			(vectorized)
Plantheight			(vectorized)
Plantheight			(vectorized)
Plantheight			(vectorized)
Plantheight			default
Plantheight		0	default
Plantheight		0	(vectorized)

Plantheight	0	(vectorized)
Plantheight	0	(vectorized)
Plantheight	0	(vectorized)
Plantheight	0	default
Spikeletlength		default
Spikeletlength		(vectorized)
Spikeletlength		(vectorized)
Spikeletlength		(vectorized)
Spikeletlength		(vectorized)
Spikeletlength		(vectorized)
Spikeletlength		(vectorized)
Spikeletlength		(vectorized)
Spikeletlength		(vectorized)
Spikeletlength		(vectorized)
Spikeletlength		(vectorized)
Spikeletlength		default
Spikeletlength	0	default
Spikeletlength	0	(vectorized)
Spikeletlength	0	(vectorized)
Spikeletlength	0	(vectorized)
Spikeletlength	0	(vectorized)
Spikeletlength	0	default
Spikeletno		default
Spikeletno		(vectorized)
Spikeletno		(vectorized)
Spikeletno		(vectorized)
Spikeletno		(vectorized)
Spikeletno		(vectorized)
Spikeletno		(vectorized)
Spikeletno		(vectorized)
Spikeletno		(vectorized)
Spikeletno		(vectorized)
Spikeletno		(vectorized)
Spikeletno		default
Spikeletno	0	default
Spikeletno	0	(vectorized)
Spikeletno	0	(vectorized)
Spikeletno	0	(vectorized)
Spikeletno	0	(vectorized)
Spikeletno	0	default

```r
# sets the priors for the coefficients to N(0,2)
# p_manual<-c(prior(normal(0, 2), class = b))

### Run -------------------------------------------------------------

model_2<-brm(
  model_2_brmsformula,
  prior = c(
    # SD priors
    prior(std_normal(),class=sd,group=phylo,resp=HeterozygositypKb,lb=0),
    prior(std_normal(),class=sd,group=Pop,resp=HeterozygositypKb,lb=0),
    prior(std_normal(),class=sd,group=Pop,resp=Plantheight,lb=0),
    prior(std_normal(),class=sd,group=Pop,resp=Spikeletno,lb=0),
    prior(std_normal(),class=sd,group=Pop,resp=Spikeletlength,lb=0),
    prior(std_normal(),class=sd,group=Ind,resp=Plantheight,lb=0),
    prior(std_normal(),class=sd,group=Ind,resp=Spikeletno,lb=0),
    prior(std_normal(),class=sd,group=Ind,resp=Spikeletlength,lb=0),
    # beta priors
    prior(std_normal(),class=b,resp=HeterozygositypKb),
    # residual corr priors
    prior(lkj_corr_cholesky(2),class=Lrescor),
    # non-default sigma prior because we place identity link on sigma
    prior(student_t(3,0,2),resp=HeterozygositypKb,dpar=sigma,lb=0)
  ),
  data = dat_modeling ,
  family = brmsfamily("gaussian",link_sigma="identity"),
  data2 = list(Cp=Cp,Ci=Ci),
  backend = "cmdstanr",
  save_pars = save_pars(all=TRUE),
  iter = 2000,
  refresh = 250,
  cores = 4,
  chains = 4,
  control = list(adapt_delta = 0.995, max_treedepth = 14),
  save_model = ".brms/model_2_rescor_C00.stan",
  file = ".brms/model_2_rescor_C00",
  seed = 42
  # stan_model_args = list(force_recompile=TRUE),
  # threads = threading(2)
  )
print(summary(model_2))
```

```
 Family: MV(gaussian, gaussian, gaussian, gaussian, gaussian)
  Links: mu = identity; sigma = identity
         mu = identity
         mu = identity
         mu = identity
         mu = identity
Formula: Heterozygosity.pKb ~ 0 + Habitat + (Climate_variability + Rainfall_CV + Rainfall_MA[illegible]
         sigma ~ 0 + Habitat
         Plant_height | mi() ~ 1 + Species + (1 | Pop) + (1 | gr(Ind, cov = Ci))
         Spikelet_no | mi() ~ 1 + Species + (1 | Pop) + (1 | gr(Ind, cov = Ci))
         Spikelet_length | mi() ~ 1 + Species + (1 | Pop) + (1 | gr(Ind, cov = Ci))
         Conspecific_isolation_log | mi() ~ 1
   Data: dat_modeling (Number of observations: 257)
  Draws: 4 chains, each with iter = 2000; warmup = 1000; thin = 1;
         total post-warmup draws = 4000

Multilevel Hyperparameters:
~phylo (Number of levels: 43)
                                         Estimate Est.Error l-95% CI u-95% CI
sd(HeterozygositypKb_Intercept:HabitatDry)     0.76      0.43     0.06     1.72
sd(HeterozygositypKb_Intercept:HabitatWet)     1.87      0.33     1.27     2.58
                                         Rhat Bulk_ESS Tail_ESS
sd(HeterozygositypKb_Intercept:HabitatDry) 1.00      879     1606
sd(HeterozygositypKb_Intercept:HabitatWet) 1.00     1448     1745

~Pop (Number of levels: 43)
                                         Estimate Est.Error l-95% CI u-95% CI
sd(HeterozygositypKb_Intercept:HabitatDry)     0.48      0.31     0.03     1.22
sd(HeterozygositypKb_Intercept:HabitatWet)     0.39      0.30     0.02     1.09
sd(Plantheight_Intercept)                      0.59      0.17     0.19     0.89
sd(Spikeletno_Intercept)                       0.54      0.17     0.14     0.84
sd(Spikeletlength_Intercept)                   0.26      0.13     0.02     0.51
                                         Rhat Bulk_ESS Tail_ESS
sd(HeterozygositypKb_Intercept:HabitatDry) 1.00     1166     1533
sd(HeterozygositypKb_Intercept:HabitatWet) 1.00      599      999
sd(Plantheight_Intercept)                  1.01      300      420
sd(Spikeletno_Intercept)                   1.01      226      373
sd(Spikeletlength_Intercept)               1.02      277     1342

~Ind (Number of levels: 257)
                            Estimate Est.Error l-95% CI u-95% CI Rhat Bulk_ESS
sd(Plantheight_Intercept)       0.57      0.30     0.04     1.10 1.01      183
sd(Spikeletno_Intercept)        0.46      0.23     0.03     0.89 1.02      152
```

```
sd(Spikeletlength_Intercept)     0.62      0.17      0.23     0.91 1.03      153
                            Tail_ESS
sd(Plantheight_Intercept)        558
sd(Spikeletno_Intercept)         698
sd(Spikeletlength_Intercept)     441

Regression Coefficients:
                                                     Estimate Est.Error
Plantheight_Intercept                                   -0.69      0.59
Spikeletno_Intercept                                    -1.25      0.48
Spikeletlength_Intercept                                -0.88      0.52
Conspecificisolationlog_Intercept                        0.00      0.07
HeterozygositypKb_HabitatDry                             2.61      0.68
HeterozygositypKb_HabitatWet                             3.01      0.76
HeterozygositypKb_HabitatDry:Climate_variability        -0.23      0.50
HeterozygositypKb_HabitatWet:Climate_variability         0.12      0.41
HeterozygositypKb_HabitatDry:Rainfall_CV                -0.37      0.55
HeterozygositypKb_HabitatWet:Rainfall_CV                -0.17      0.48
HeterozygositypKb_HabitatDry:Rainfall_MAP               -0.29      0.57
HeterozygositypKb_HabitatWet:Rainfall_MAP                0.40      0.37
HeterozygositypKb_HabitatDry:Origin_isolation_log       -0.09      0.39
HeterozygositypKb_HabitatWet:Origin_isolation_log        0.12      0.50
HeterozygositypKb_HabitatDry:Height_above_drainage       0.29      0.35
HeterozygositypKb_HabitatWet:Height_above_drainage      -0.06      0.26
sigma_HeterozygositypKb_HabitatDry                       0.46      0.10
sigma_HeterozygositypKb_HabitatWet                       0.48      0.10
Plantheight_SpeciesEasternRupestris                      0.56      0.92
Plantheight_SpeciesFernkloofA                           -0.20      0.84
Plantheight_SpeciesFernkloofB                            0.39      1.09
Plantheight_SpeciesLeafyTricostata                       0.48      0.68
Plantheight_SpeciesRestioidTricostata                    1.25      0.87
Plantheight_SpeciesScabra                                0.82      0.86
Plantheight_SpeciesSetacea                               1.13      0.66
Plantheight_SpeciesUniflora                              2.24      0.92
Plantheight_SpeciesWemmershoek                           0.55      0.96
Plantheight_SpeciesWesternRupestris                      0.30      0.84
Spikeletno_SpeciesEasternRupestris                       1.35      0.77
Spikeletno_SpeciesFernkloofA                             0.62      0.66
Spikeletno_SpeciesFernkloofB                             1.36      0.92
Spikeletno_SpeciesLeafyTricostata                        1.32      0.58
Spikeletno_SpeciesRestioidTricostata                     1.52      0.72
Spikeletno_SpeciesScabra                                 2.28      0.70
Spikeletno_SpeciesSetacea                                1.50      0.54
```

```
Spikeletno_SpeciesUniflora                                   0.32      0.78
Spikeletno_SpeciesWemmershoek                                0.66      0.82
Spikeletno_SpeciesWesternRupestris                           1.46      0.71
Spikeletlength_SpeciesEasternRupestris                       0.64      0.79
Spikeletlength_SpeciesFernkloofA                             0.18      0.66
Spikeletlength_SpeciesFernkloofB                             3.18      0.87
Spikeletlength_SpeciesLeafyTricostata                        0.84      0.56
Spikeletlength_SpeciesRestioidTricostata                     0.73      0.76
Spikeletlength_SpeciesScabra                                 2.16      0.72
Spikeletlength_SpeciesSetacea                                1.07      0.55
Spikeletlength_SpeciesUniflora                              -0.22      0.81
Spikeletlength_SpeciesWemmershoek                            1.18      0.72
Spikeletlength_SpeciesWesternRupestris                       0.33      0.74
HeterozygositypKb_HabitatDry:miConspecific_isolation_log    -0.21      0.41
HeterozygositypKb_HabitatWet:miConspecific_isolation_log    -0.04      0.26
HeterozygositypKb_HabitatDry:miPlant_height                 -0.31      0.26
HeterozygositypKb_HabitatWet:miPlant_height                 -0.31      0.27
HeterozygositypKb_HabitatDry:miSpikelet_no                   0.03      0.24
HeterozygositypKb_HabitatWet:miSpikelet_no                   0.08      0.28
HeterozygositypKb_HabitatDry:miSpikelet_length               0.45      0.24
HeterozygositypKb_HabitatWet:miSpikelet_length               0.41      0.29
                                                         l-95% CI u-95% CI Rhat
Plantheight_Intercept                                       -1.85     0.52 1.00
Spikeletno_Intercept                                        -2.20    -0.26 1.01
Spikeletlength_Intercept                                    -1.93     0.14 1.00
Conspecificisolationlog_Intercept                           -0.13     0.13 1.00
HeterozygositypKb_HabitatDry                                 1.02     3.69 1.00
HeterozygositypKb_HabitatWet                                 1.41     4.36 1.00
HeterozygositypKb_HabitatDry:Climate_variability            -1.20     0.77 1.00
HeterozygositypKb_HabitatWet:Climate_variability            -0.67     0.94 1.01
HeterozygositypKb_HabitatDry:Rainfall_CV                    -1.45     0.73 1.00
HeterozygositypKb_HabitatWet:Rainfall_CV                    -1.11     0.76 1.00
HeterozygositypKb_HabitatDry:Rainfall_MAP                   -1.39     0.88 1.01
HeterozygositypKb_HabitatWet:Rainfall_MAP                   -0.32     1.14 1.00
HeterozygositypKb_HabitatDry:Origin_isolation_log           -0.91     0.63 1.00
HeterozygositypKb_HabitatWet:Origin_isolation_log           -0.84     1.11 1.00
HeterozygositypKb_HabitatDry:Height_above_drainage          -0.35     1.09 1.00
HeterozygositypKb_HabitatWet:Height_above_drainage          -0.60     0.44 1.00
sigma_HeterozygositypKb_HabitatDry                           0.33     0.69 1.00
sigma_HeterozygositypKb_HabitatWet                           0.36     0.74 1.00
Plantheight_SpeciesEasternRupestris                         -1.21     2.43 1.00
Plantheight_SpeciesFernkloofA                               -1.92     1.45 1.01
Plantheight_SpeciesFernkloofB                               -1.80     2.57 1.00
```

Plantheight_SpeciesLeafyTricostata	-0.86	1.85	1.00
Plantheight_SpeciesRestioidTricostata	-0.45	3.05	1.00
Plantheight_SpeciesScabra	-0.95	2.58	1.00
Plantheight_SpeciesSetacea	-0.20	2.45	1.00
Plantheight_SpeciesUniflora	0.36	4.15	1.00
Plantheight_SpeciesWemmershoek	-1.35	2.45	1.00
Plantheight_SpeciesWesternRupestris	-1.38	2.00	1.00
Spikeletno_SpeciesEasternRupestris	-0.12	2.92	1.00
Spikeletno_SpeciesFernkloofA	-0.70	1.95	1.01
Spikeletno_SpeciesFernkloofB	-0.46	3.15	1.00
Spikeletno_SpeciesLeafyTricostata	0.17	2.48	1.00
Spikeletno_SpeciesRestioidTricostata	0.12	3.01	1.01
Spikeletno_SpeciesScabra	0.87	3.63	1.00
Spikeletno_SpeciesSetacea	0.43	2.56	1.00
Spikeletno_SpeciesUniflora	-1.20	1.86	1.01
Spikeletno_SpeciesWemmershoek	-0.96	2.31	1.00
Spikeletno_SpeciesWesternRupestris	0.06	2.85	1.00
Spikeletlength_SpeciesEasternRupestris	-0.95	2.22	1.00
Spikeletlength_SpeciesFernkloofA	-1.16	1.51	1.00
Spikeletlength_SpeciesFernkloofB	1.44	4.88	1.00
Spikeletlength_SpeciesLeafyTricostata	-0.27	1.95	1.00
Spikeletlength_SpeciesRestioidTricostata	-0.76	2.24	1.00
Spikeletlength_SpeciesScabra	0.73	3.57	1.01
Spikeletlength_SpeciesSetacea	-0.02	2.14	1.00
Spikeletlength_SpeciesUniflora	-1.90	1.37	1.00
Spikeletlength_SpeciesWemmershoek	-0.25	2.59	1.00
Spikeletlength_SpeciesWesternRupestris	-1.12	1.76	1.01
HeterozygositypKb_HabitatDry:miConspecific_isolation_log	-1.06	0.58	1.00
HeterozygositypKb_HabitatWet:miConspecific_isolation_log	-0.54	0.46	1.01
HeterozygositypKb_HabitatDry:miPlant_height	-0.80	0.23	1.01
HeterozygositypKb_HabitatWet:miPlant_height	-0.81	0.26	1.01
HeterozygositypKb_HabitatDry:miSpikelet_no	-0.44	0.51	1.01
HeterozygositypKb_HabitatWet:miSpikelet_no	-0.47	0.63	1.01
HeterozygositypKb_HabitatDry:miSpikelet_length	-0.01	0.94	1.03
HeterozygositypKb_HabitatWet:miSpikelet_length	-0.19	0.96	1.04

	Bulk_ESS	Tail_ESS
Plantheight_Intercept	689	1116
Spikeletno_Intercept	653	1247
Spikeletlength_Intercept	693	890
Conspecificisolationlog_Intercept	4805	3198
HeterozygositypKb_HabitatDry	1570	2390
HeterozygositypKb_HabitatWet	1617	2283
HeterozygositypKb_HabitatDry:Climate_variability	2163	2893

HeterozygositypKb_HabitatWet:Climate_variability	1576	2273
HeterozygositypKb_HabitatDry:Rainfall_CV	2337	2496
HeterozygositypKb_HabitatWet:Rainfall_CV	1459	2032
HeterozygositypKb_HabitatDry:Rainfall_MAP	1396	1892
HeterozygositypKb_HabitatWet:Rainfall_MAP	1408	1953
HeterozygositypKb_HabitatDry:Origin_isolation_log	1123	2306
HeterozygositypKb_HabitatWet:Origin_isolation_log	1440	2111
HeterozygositypKb_HabitatDry:Height_above_drainage	1684	2405
HeterozygositypKb_HabitatWet:Height_above_drainage	1063	1446
sigma_HeterozygositypKb_HabitatDry	1084	1270
sigma_HeterozygositypKb_HabitatWet	879	1304
Plantheight_SpeciesEasternRupestris	1131	1751
Plantheight_SpeciesFernkloofA	527	1001
Plantheight_SpeciesFernkloofB	1448	2023
Plantheight_SpeciesLeafyTricostata	1308	1995
Plantheight_SpeciesRestioidTricostata	1105	1228
Plantheight_SpeciesScabra	945	1498
Plantheight_SpeciesSetacea	848	1461
Plantheight_SpeciesUniflora	1020	1536
Plantheight_SpeciesWemmershoek	2294	2426
Plantheight_SpeciesWesternRupestris	988	1377
Spikeletno_SpeciesEasternRupestris	1198	1865
Spikeletno_SpeciesFernkloofA	678	1119
Spikeletno_SpeciesFernkloofB	1605	2141
Spikeletno_SpeciesLeafyTricostata	1310	1954
Spikeletno_SpeciesRestioidTricostata	1017	1664
Spikeletno_SpeciesScabra	891	1184
Spikeletno_SpeciesSetacea	1097	1735
Spikeletno_SpeciesUniflora	1238	1548
Spikeletno_SpeciesWemmershoek	1865	2577
Spikeletno_SpeciesWesternRupestris	1157	1769
Spikeletlength_SpeciesEasternRupestris	1292	1803
Spikeletlength_SpeciesFernkloofA	801	1092
Spikeletlength_SpeciesFernkloofB	1209	1703
Spikeletlength_SpeciesLeafyTricostata	1085	1730
Spikeletlength_SpeciesRestioidTricostata	1172	1604
Spikeletlength_SpeciesScabra	970	1626
Spikeletlength_SpeciesSetacea	1169	1655
Spikeletlength_SpeciesUniflora	1227	1721
Spikeletlength_SpeciesWemmershoek	2311	2697
Spikeletlength_SpeciesWesternRupestris	1000	1621
HeterozygositypKb_HabitatDry:miConspecific_isolation_log	1152	1649
HeterozygositypKb_HabitatWet:miConspecific_isolation_log	725	1044

```
HeterozygositypKb_HabitatDry:miPlant_height                  415      826
HeterozygositypKb_HabitatWet:miPlant_height                  340      850
HeterozygositypKb_HabitatDry:miSpikelet_no                   666     1600
HeterozygositypKb_HabitatWet:miSpikelet_no                   568     1120
HeterozygositypKb_HabitatDry:miSpikelet_length               266      506
HeterozygositypKb_HabitatWet:miSpikelet_length               204      444

Further Distributional Parameters:
                              Estimate Est.Error l-95% CI u-95% CI Rhat
sigma_Plantheight                 0.54      0.05     0.45     0.65 1.01
sigma_Spikeletno                  0.45      0.04     0.38     0.53 1.00
sigma_Spikeletlength              0.40      0.04     0.33     0.48 1.00
sigma_Conspecificisolationlog     1.01      0.05     0.92     1.11 1.00
                              Bulk_ESS Tail_ESS
sigma_Plantheight                  813     1901
sigma_Spikeletno                   781     2049
sigma_Spikeletlength               517     1113
sigma_Conspecificisolationlog     5045     2977

Residual Correlations:
                                                  Estimate Est.Error l-95% CI
rescor(HeterozygositypKb,Plantheight)                 0.21      0.32    -0.46
rescor(HeterozygositypKb,Spikeletno)                  0.02      0.26    -0.49
rescor(Plantheight,Spikeletno)                        0.18      0.12    -0.08
rescor(HeterozygositypKb,Spikeletlength)             -0.17      0.28    -0.68
rescor(Plantheight,Spikeletlength)                    0.16      0.12    -0.09
rescor(Spikeletno,Spikeletlength)                     0.06      0.11    -0.15
rescor(HeterozygositypKb,Conspecificisolationlog)     0.02      0.39    -0.72
rescor(Plantheight,Conspecificisolationlog)           0.04      0.40    -0.64
rescor(Spikeletno,Conspecificisolationlog)            0.14      0.19    -0.24
rescor(Spikeletlength,Conspecificisolationlog)        0.14      0.19    -0.23
                                                  u-95% CI Rhat Bulk_ESS
rescor(HeterozygositypKb,Plantheight)                 0.73 1.01      441
rescor(HeterozygositypKb,Spikeletno)                  0.50 1.01      688
rescor(Plantheight,Spikeletno)                        0.40 1.00      994
rescor(HeterozygositypKb,Spikeletlength)              0.39 1.03      227
rescor(Plantheight,Spikeletlength)                    0.38 1.00      915
rescor(Spikeletno,Spikeletlength)                     0.27 1.00     1431
rescor(HeterozygositypKb,Conspecificisolationlog)     0.71 1.00      882
rescor(Plantheight,Conspecificisolationlog)           0.70 1.02      195
rescor(Spikeletno,Conspecificisolationlog)            0.50 1.01      651
rescor(Spikeletlength,Conspecificisolationlog)        0.48 1.00     1235
                                                  Tail_ESS
```

```
rescor(HeterozygositypKb,Plantheight)                  862
rescor(HeterozygositypKb,Spikeletno)                  1406
rescor(Plantheight,Spikeletno)                        2108
rescor(HeterozygositypKb,Spikeletlength)               566
rescor(Plantheight,Spikeletlength)                    1947
rescor(Spikeletno,Spikeletlength)                     1970
rescor(HeterozygositypKb,Conspecificisolationlog)     1227
rescor(Plantheight,Conspecificisolationlog)            494
rescor(Spikeletno,Conspecificisolationlog)            2089
rescor(Spikeletlength,Conspecificisolationlog)        2015

Draws were sampled using sample(hmc). For each parameter, Bulk_ESS
and Tail_ESS are effective sample size measures, and Rhat is the potential
scale reduction factor on split chains (at convergence, Rhat = 1).
```

```r
get_prior(model_2)
```

```
                prior     class                                 coef group
               lkj(1)    rescor
 student_t(3, 0, 2.5) Intercept
 student_t(3, 0, 2.5)     sigma
               (flat)         b
               (flat)         b                           HabitatDry
               (flat)         b         HabitatDry:Climate_variability
               (flat)         b       HabitatDry:Height_above_drainage
               (flat)         b HabitatDry:miConspecific_isolation_log
               (flat)         b              HabitatDry:miPlant_height
               (flat)         b           HabitatDry:miSpikelet_length
               (flat)         b               HabitatDry:miSpikelet_no
               (flat)         b        HabitatDry:Origin_isolation_log
               (flat)         b                 HabitatDry:Rainfall_CV
               (flat)         b                HabitatDry:Rainfall_MAP
               (flat)         b                           HabitatWet
               (flat)         b         HabitatWet:Climate_variability
               (flat)         b       HabitatWet:Height_above_drainage
               (flat)         b HabitatWet:miConspecific_isolation_log
               (flat)         b              HabitatWet:miPlant_height
               (flat)         b           HabitatWet:miSpikelet_length
               (flat)         b               HabitatWet:miSpikelet_no
               (flat)         b        HabitatWet:Origin_isolation_log
               (flat)         b                 HabitatWet:Rainfall_CV
               (flat)         b                HabitatWet:Rainfall_MAP
```

student_t(3, 0, 2.5)	sd		
student_t(3, 0, 2.5)	sd		phylo
student_t(3, 0, 2.5)	sd	Intercept	phylo
student_t(3, 0, 2.5)	sd		Pop
student_t(3, 0, 2.5)	sd	Intercept	Pop
(flat)	b		
(flat)	b	HabitatDry	
(flat)	b	HabitatWet	
(flat)	b		
(flat)	b	SpeciesEasternRupestris	
(flat)	b	SpeciesFernkloofA	
(flat)	b	SpeciesFernkloofB	
(flat)	b	SpeciesLeafyTricostata	
(flat)	b	SpeciesRestioidTricostata	
(flat)	b	SpeciesScabra	
(flat)	b	SpeciesSetacea	
(flat)	b	SpeciesUniflora	
(flat)	b	SpeciesWemmershoek	
(flat)	b	SpeciesWesternRupestris	
student_t(3, 0, 2.5)	Intercept		
student_t(3, 0, 2.5)	sd		
student_t(3, 0, 2.5)	sd		Ind
student_t(3, 0, 2.5)	sd	Intercept	Ind
student_t(3, 0, 2.5)	sd		Pop
student_t(3, 0, 2.5)	sd	Intercept	Pop
student_t(3, 0, 2.5)	sigma		
(flat)	b		
(flat)	b	SpeciesEasternRupestris	
(flat)	b	SpeciesFernkloofA	
(flat)	b	SpeciesFernkloofB	
(flat)	b	SpeciesLeafyTricostata	
(flat)	b	SpeciesRestioidTricostata	
(flat)	b	SpeciesScabra	
(flat)	b	SpeciesSetacea	
(flat)	b	SpeciesUniflora	
(flat)	b	SpeciesWemmershoek	
(flat)	b	SpeciesWesternRupestris	
student_t(3, 0, 2.5)	Intercept		
student_t(3, 0, 2.5)	sd		
student_t(3, 0, 2.5)	sd		Ind
student_t(3, 0, 2.5)	sd	Intercept	Ind
student_t(3, 0, 2.5)	sd		Pop
student_t(3, 0, 2.5)	sd	Intercept	Pop

```
student_t(3, 0, 2.5)     sigma
              (flat)         b
              (flat)         b                SpeciesEasternRupestris
              (flat)         b                      SpeciesFernkloofA
              (flat)         b                      SpeciesFernkloofB
              (flat)         b                 SpeciesLeafyTricostata
              (flat)         b              SpeciesRestioidTricostata
              (flat)         b                          SpeciesScabra
              (flat)         b                         SpeciesSetacea
              (flat)         b                        SpeciesUniflora
              (flat)         b                     SpeciesWemmershoek
              (flat)         b                SpeciesWesternRupestris
student_t(3, 0, 2.5) Intercept
student_t(3, 0, 2.5)        sd
student_t(3, 0, 2.5)        sd                                          Ind
student_t(3, 0, 2.5)        sd                              Intercept   Ind
student_t(3, 0, 2.5)        sd                                          Pop
student_t(3, 0, 2.5)        sd                              Intercept   Pop
student_t(3, 0, 2.5)     sigma
                   resp  dpar nlpar lb ub tag       source
                                                   default
Conspecificisolationlog                            default
Conspecificisolationlog              0             default
      HeterozygositypKb                            default
      HeterozygositypKb                       (vectorized)
      HeterozygositypKb                       (vectorized)
      HeterozygositypKb                       (vectorized)
      HeterozygositypKb                       (vectorized)
      HeterozygositypKb                       (vectorized)
      HeterozygositypKb                       (vectorized)
      HeterozygositypKb                       (vectorized)
      HeterozygositypKb                       (vectorized)
      HeterozygositypKb                       (vectorized)
      HeterozygositypKb                       (vectorized)
      HeterozygositypKb                       (vectorized)
      HeterozygositypKb                       (vectorized)
      HeterozygositypKb                       (vectorized)
      HeterozygositypKb                       (vectorized)
      HeterozygositypKb                       (vectorized)
      HeterozygositypKb                       (vectorized)
      HeterozygositypKb                       (vectorized)
      HeterozygositypKb                       (vectorized)
      HeterozygositypKb                       (vectorized)
```

HeterozygositypKb		(vectorized)
HeterozygositypKb	0	default
HeterozygositypKb	0	(vectorized)
HeterozygositypKb	0	(vectorized)
HeterozygositypKb	0	(vectorized)
HeterozygositypKb	0	(vectorized)
HeterozygositypKb sigma		default
HeterozygositypKb sigma		(vectorized)
HeterozygositypKb sigma		(vectorized)
Plantheight		default
Plantheight		(vectorized)
Plantheight		(vectorized)
Plantheight		(vectorized)
Plantheight		(vectorized)
Plantheight		(vectorized)
Plantheight		(vectorized)
Plantheight		(vectorized)
Plantheight		(vectorized)
Plantheight		(vectorized)
Plantheight		(vectorized)
Plantheight		default
Plantheight	0	default
Plantheight	0	(vectorized)
Plantheight	0	(vectorized)
Plantheight	0	(vectorized)
Plantheight	0	(vectorized)
Plantheight	0	default
Spikeletlength		default
Spikeletlength		(vectorized)
Spikeletlength		(vectorized)
Spikeletlength		(vectorized)
Spikeletlength		(vectorized)
Spikeletlength		(vectorized)
Spikeletlength		(vectorized)
Spikeletlength		(vectorized)
Spikeletlength		(vectorized)
Spikeletlength		(vectorized)
Spikeletlength		(vectorized)
Spikeletlength		default
Spikeletlength	0	default
Spikeletlength	0	(vectorized)
Spikeletlength	0	(vectorized)
Spikeletlength	0	(vectorized)

```
     Spikeletlength            0       (vectorized)
     Spikeletlength            0            default
         Spikeletno                         default
         Spikeletno                    (vectorized)
         Spikeletno                    (vectorized)
         Spikeletno                    (vectorized)
         Spikeletno                    (vectorized)
         Spikeletno                    (vectorized)
         Spikeletno                    (vectorized)
         Spikeletno                    (vectorized)
         Spikeletno                    (vectorized)
         Spikeletno                    (vectorized)
         Spikeletno                    (vectorized)
         Spikeletno                         default
         Spikeletno            0            default
         Spikeletno            0       (vectorized)
         Spikeletno            0       (vectorized)
         Spikeletno            0       (vectorized)
         Spikeletno            0       (vectorized)
         Spikeletno            0            default
```

```
sink("results/model_2_brmssummary_rescor_C00.txt")
print(model_2,digits=3)
sink()

# system2("open","results/model_2_brmssummary_rescor_C00.txt")
```

```
pp_check(model_2,ndraws=100,resp="HeterozygositypKb")
pp_check(model_2,ndraws=100,resp="Plantheight")
pp_check(model_2,ndraws=100,resp="Spikeletno")
pp_check(model_2,ndraws=100,resp="Spikeletlength")
pp_check(model_2,ndraws=100,resp="Conspecificisolationlog")
```

Post-process model 2.

```
#|echo: false

## Estimate lambda!
model_2 %>% as_tibble() %>%
  dplyr::select(sigma_b = `sd_phylo__HeterozygositypKb_Intercept:HabitatWet`,
                sigma_p = `sd_Pop__HeterozygositypKb_Intercept:HabitatWet`,
```

```r
                sigma_e = `b_sigma_HeterozygositypKb_HabitatWet`) %>%
  mutate(h2 = sigma_b^2/(sigma_b^2 + sigma_p^2 + sigma_e^2),
         hpop = sigma_p^2/(sigma_b^2 + sigma_p^2 + sigma_e^2)) %>%
  # select(h2,hpop,sigma_b,sigma_p,sigma_e) %>%
  select(h2) %>%
  map(.,\(x)print(mean_qi(x,.width=c(.89,.95))))
```

```
          y      ymin      ymax .width .point .interval
1 0.8726516 0.6838813 0.9619903   0.89   mean        qi
2 0.8726516 0.5895123 0.9670167   0.95   mean        qi


$h2
          y      ymin      ymax .width .point .interval
1 0.8726516 0.6838813 0.9619903   0.89   mean        qi
2 0.8726516 0.5895123 0.9670167   0.95   mean        qi
```

```r
model_2 %>% as_tibble() %>%
  dplyr::select(sigma_b = `sd_phylo__HeterozygositypKb_Intercept:HabitatDry`,
                sigma_p = `sd_Pop__HeterozygositypKb_Intercept:HabitatDry`,
                sigma_e = `b_sigma_HeterozygositypKb_HabitatDry`) %>%
  mutate(h2 = sigma_b^2/(sigma_b^2 + sigma_p^2 + sigma_e^2),
         hpop = sigma_p^2/(sigma_b^2 + sigma_p^2 + sigma_e^2)) %>%
  # select(h2,hpop,sigma_b,sigma_p,sigma_e) %>%
  select(h2) %>%
  map(.,\(x)print(mean_qi(x,.width=c(.89,.95))))
```

```
          y        ymin      ymax .width .point .interval
1 0.5015052 0.027829577 0.8925043   0.89   mean        qi
2 0.5015052 0.004976098 0.9213418   0.95   mean        qi


$h2
          y        ymin      ymax .width .point .interval
1 0.5015052 0.027829577 0.8925043   0.89   mean        qi
2 0.5015052 0.004976098 0.9213418   0.95   mean        qi
```

```r
marginal_R2_model_2<-bayes_R2(model_2,
                              resp="HeterozygositypKb",
                              re_formula=NA)
marginal_R2_model_2
```

```
                     Estimate   Est.Error      Q2.5     Q97.5
R2HeterozygositypKb 0.2757837 0.08226299 0.1364574 0.4563659
```

```r
### Slopes ----
## note: Always specify the variables explicitly to avoid computing
## estimates for the grouping variables (i.e., random effects)!
vars_model<-c(
  "Rainfall_MAP",
  "Rainfall_CV",
  "Climate_variability",
  "Spikelet_length",
  "Spikelet_no",
  "Plant_height",
  # "Dispersal_distance",
  "Conspecific_isolation_log",
  "Origin_isolation_log",
  "Height_above_drainage")

avg_slopes(
  model_2,
  variables = "Spikelet_length",
  # by="Habitat",
  re_formula=NULL,
  resp="HeterozygositypKb"
  )
```

```
 Estimate  2.5 % 97.5 %
    0.424 -0.107  0.939

Term: Spikelet_length
Type: response
Comparison: dY/dX
```

```r
model_2_marginal_habitat_slopes<-avg_slopes(
  model_2,
  variables = vars_model,
  by="Habitat",
  re_formula=NA,
  # re_formula=NULL,
  resp="HeterozygositypKb")
```

```r
sink("results/model_2_COO_slopes.txt")
print(model_2_marginal_habitat_slopes)
sink()

model_2_marginal_habitat_slopes_draws <-
  model_2_marginal_habitat_slopes |>
  posterior_draws()

### Plot slopes ----
to_levs<-c(
    "Habitat: Wet - Dry",

    "Rainfall_MAP",

    "Height_above_drainage",

    "Rainfall_CV",

    "Climate_variability",

    "Spikelet_length",
    "Spikelet_no",
    "Plant_height",

    # "Dispersal_distance",
    "Conspecific_isolation_log",
    "Origin_isolation_log"

    )

palette_wetdry<-c(Dry="#e64b35",Wet="#4dbbd5")
base<-model_2_marginal_habitat_slopes_draws %>%
  # filter(!term %in% c("Pop","phylo",
  #                     "Heterozygosity.pKb",
  #                     "Habitat",
  #                     "Ind")) %>%
  mutate(term=fct_relevel(term,rev(to_levs))) %>%
  ggplot(aes(x=draw,y=term,fill=Habitat))+
  labs(x="Effect on IGH",y="Term")+
  theme_bw()+
  guides(fill=guide_legend(override.aes = list(size=NA)))+
  scale_fill_manual(values=palette_wetdry)
```

```
Warning: There was 1 warning in `mutate()`.
i In argument: `term = fct_relevel(term, rev(to_levs))`.
Caused by warning:
! 1 unknown level in `f`: Habitat: Wet - Dry
```

```
model_2_habitat_plot<-base+
  stat_eye(slab_alpha=.5,shape=21,
           normalize="xy",
           position = "dodgejust",
           height=.75,
           point_interval = mode_hdi,
           .width = c(0.66, 0.95)
  )+
  geom_vline(xintercept = 0,linetype=3)+
  scale_x_continuous(breaks = scales::extended_breaks(10),
                     limits=c(-3,3))

model_2_habitat_plot
```

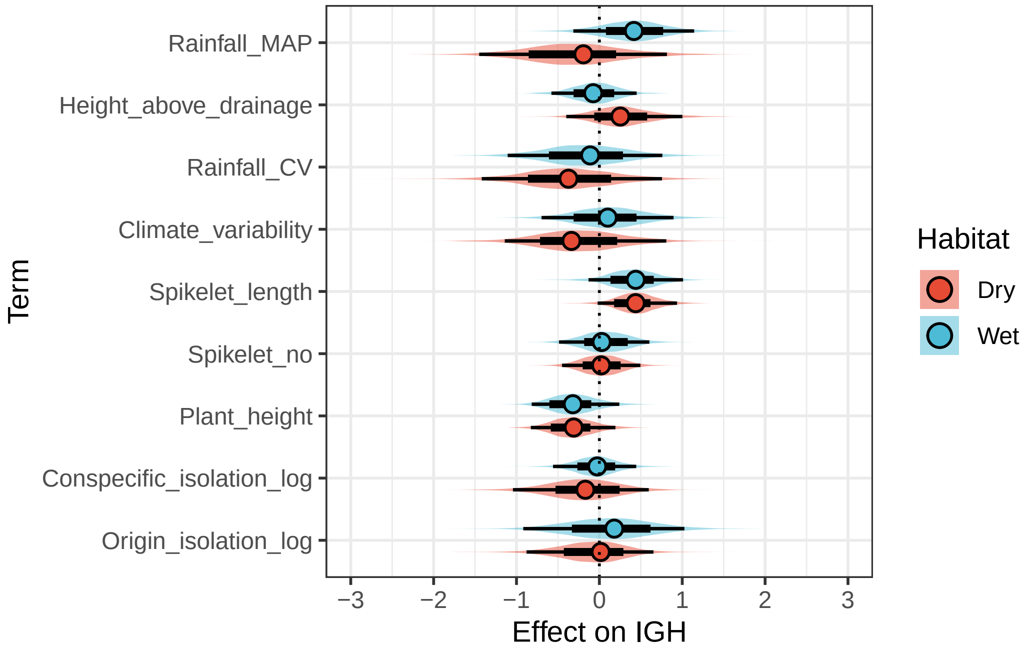


```
ggsave("plots/MarginalEffects_slabs_model_2_rescor_COO.pdf",
       plot=model_2_habitat_plot,
       width=24,height=20,units="cm")
```

```r
ggsave("plots/MarginalEffects_slabs_model_2_rescor_COO.png",
       plot=model_2_habitat_plot,
       width=24,height=20,units="cm")
# system2("open","plots/MarginalEffects_slabs_model_2_rescor_COO.pdf")
```

Plot Models 1 and 2 together

```r
from_names <- c(
  "Habitat",
  "Spikelet_length",
  "Spikelet_no",
  "Plant_height",
  "Climate_variability",
  "Rainfall_CV",
  "Rainfall_MAP",
  "Height_above_drainage",
  "Conspecific_isolation_log",
  "Origin_isolation_log"
)

to_names <- c(
  "Habitat: Wet - Dry",
  "SPL",
  "SPN",
  "PLH",
  "CII",
  "CVAP",
  "MAP",
  "HAND",
  "ISO_C",
  "ISO_O"
)

unique(model_1_slopes_draws$term)
```

```
 [1] "Climate_variability"       "Conspecific_isolation_log"
 [3] "Habitat"                   "Height_above_drainage"
 [5] "Origin_isolation_log"      "Plant_height"
 [7] "Rainfall_CV"               "Rainfall_MAP"
 [9] "Spikelet_length"           "Spikelet_no"
```

```
model_1_slopes_draws$Term <- model_1_slopes_draws$term
model_2_marginal_habitat_slopes_draws$Term <- model_2_marginal_habitat_slopes_draws$term
for(i in seq_along(from_names)){
  model_1_slopes_draws$Term[model_1_slopes_draws$term==from_names[i]] <- to_names[i]
  model_2_marginal_habitat_slopes_draws$Term[model_2_marginal_habitat_slopes_draws$term==fro
}

unique(model_1_slopes_draws$Term)
```

```
 [1] "CII"                    "ISO_C"                  "Habitat: Wet - Dry"
 [4] "HAND"                   "ISO_O"                  "PLH"
 [7] "CVAP"                   "MAP"                    "SPL"
[10] "SPN"
```

```
unique(model_2_marginal_habitat_slopes_draws$Term)
```

```
[1] "CII"   "ISO_C" "HAND"  "ISO_O" "PLH"   "CVAP"  "MAP"   "SPL"   "SPN"
```

```
palette_wetdry<-c("#4dbbd5","#e64b35")
palette_wetdry_3 <- c(palette_wetdry, "darkgreen")
palette_wetdry_3 <- scales::alpha(palette_wetdry_3, 0.7)
p_both <- bind_rows(
  model_1_slopes_draws %>%
    mutate(Model = "Model 1"),
  model_2_marginal_habitat_slopes_draws %>%
    mutate(Model = "Model 2")
) %>%
  mutate(Term = fct_relevel(Term, rev(to_names)), Model = fct_rev(Model)) %>%
  mutate(Habitat = case_when(is.na(Habitat) ~ "", .default = Habitat)) %>%
  mutate(Habitat = fct_relevel(Habitat, c("Wet", "Dry", ""))) %>%
  ggplot(aes(
    x = draw,
    y = Term,
    shape = Model,
    fill = Habitat
  )) +
  geom_vline(xintercept = 0, linetype = 3) +
  stat_eye(
    position = position_dodge(width = .7),
    normalize = "xy",
```

```r
    scale = .65,
    point_fill = "white"
  ) +
  theme_bw() +
  theme(axis.text.y = element_text(size = 12),
        legend.text = element_text(size = 10)) +
  guides(
    fill = guide_legend(override.aes = list(
      size = NA,
      shape = NA,
      fill = c(palette_wetdry, NA)
    )),
    shape = guide_legend(
      override.aes = list(size = 6, linetype = NA),
      reverse = TRUE
    )
  ) +
  scale_shape_manual(values = c(21:23)) +
  scale_fill_manual(values = palette_wetdry_3) +
  labs(x = "Effect on IGH", y = "Term") +
  coord_cartesian(xlim = c(-3, 3))
p_both
```

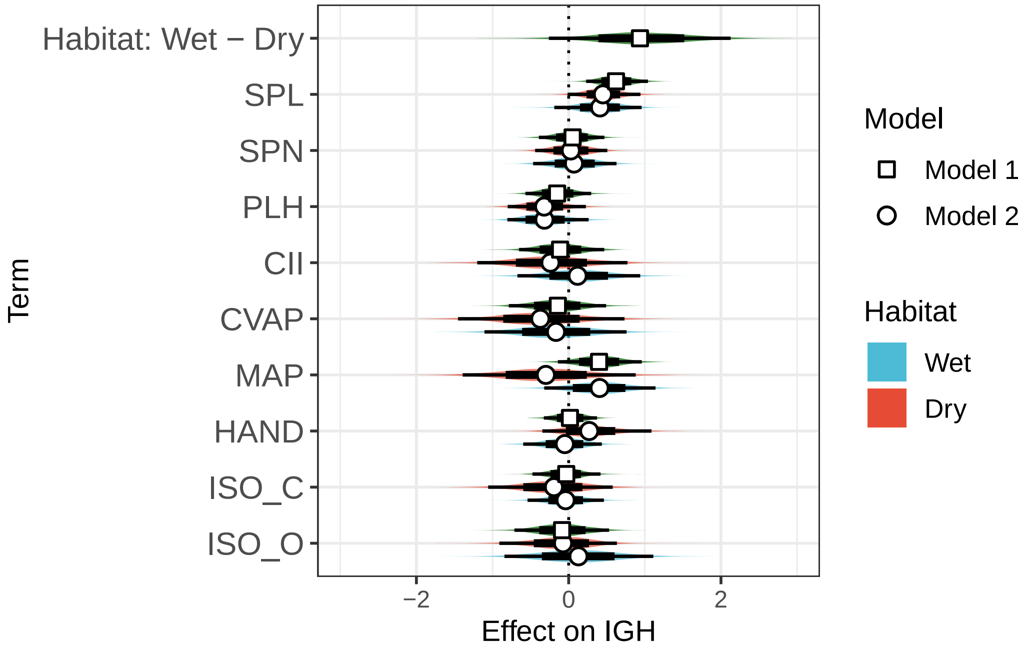

```r
ggsave("plots/MarginalEffects_slabs_models_1and2_C00.pdf",
       width=24,height=20,units="cm",plot=p_both)
ggsave("plots/MarginalEffects_slabs_models_1and2_C00.png",
       width=24,height=20,units="cm",plot=p_both)
# system2("open","plots/MarginalEffects_slabs_models_1and2_C00.pdf")
```

Model comparison and summaries

Null Model

```r
model_null_brmsformula<-bf(
  Heterozygosity.pKb ~
    1 +
    (1 | gr( phylo, cov=Cp )) +
    (1 | Pop),
  family = "gaussian" ## <--- Standard regression
)

### Run -------------------------------------------------------------

model_null<-brm(model_null_brmsformula,
             data = dat_modeling,
             data2 = list(Cp=Cp),
             backend = "cmdstanr",
             save_pars = save_pars(all=TRUE),
             iter = 3000,
             cores = 4,
             chains = 4,
             control = list(adapt_delta = 0.95, max_treedepth = 12),
             save_model = ".brms/model_null.stan",
             file = ".brms/model_null")
print(summary(model_null))
```

```
Warning: There were 2 divergent transitions after warmup. Increasing
adapt_delta above 0.95 may help. See
http://mc-stan.org/misc/warnings.html#divergent-transitions-after-warmup
```

```
 Family: gaussian
  Links: mu = identity
```

```
Formula: Heterozygosity.pKb | trunc(lb = 0) ~ 1 + (1 | gr(phylo, cov = Cp)) + (1 | Pop)
   Data: dat_modeling (Number of observations: 257)
  Draws: 4 chains, each with iter = 3000; warmup = 1500; thin = 1;
         total post-warmup draws = 6000

Multilevel Hyperparameters:
~phylo (Number of levels: 43)
              Estimate Est.Error l-95% CI u-95% CI Rhat Bulk_ESS Tail_ESS
sd(Intercept)     1.61      0.22     1.21     2.07 1.00     1391     1696

~Pop (Number of levels: 43)
              Estimate Est.Error l-95% CI u-95% CI Rhat Bulk_ESS Tail_ESS
sd(Intercept)     0.33      0.24     0.01     0.86 1.01      358      668

Regression Coefficients:
          Estimate Est.Error l-95% CI u-95% CI Rhat Bulk_ESS Tail_ESS
Intercept     4.45      0.55     3.40     5.54 1.00     1587     2562

Further Distributional Parameters:
      Estimate Est.Error l-95% CI u-95% CI Rhat Bulk_ESS Tail_ESS
sigma     0.36      0.02     0.33     0.40 1.00     5794     4142

Draws were sampled using sample(hmc). For each parameter, Bulk_ESS
and Tail_ESS are effective sample size measures, and Rhat is the potential
scale reduction factor on split chains (at convergence, Rhat = 1).
```

```r
bayes_R2(model_null,re_formula = ~ (1|gr(phylo,cov=Cp)))
```

```
    Estimate  Est.Error      Q2.5     Q97.5
R2 0.8959467 0.07216821 0.6956306 0.9537393
```

```r
bayes_R2(model_2, resp="HeterozygositypKb",re_formula= ~ (1|gr(phylo,cov=Cp)))
```

```
                  Estimate  Est.Error      Q2.5     Q97.5
R2HeterozygositypKb 0.8620849 0.06385886 0.6887293 0.9391391
```

R-squared

Not run for compilation.

```r
## Model 1
conditional_R2_model_1 <- bayes_R2(
  model_1,
  resp = "HeterozygositypKb",
  re_formula = ~ (1 | Pop) + (1 | gr(phylo, cov = Cp))
)
conditional_R2_model_1
```

```
                     Estimate  Est.Error      Q2.5     Q97.5
R2HeterozygositypKb 0.9262921 0.02295418 0.8694329 0.9533609
```

```r
marginal_R2_model_1 <- bayes_R2(
  model_1,
  resp = "HeterozygositypKb",
  re_formula = NA
)
marginal_R2_model_1
```

```
                    Estimate  Est.Error      Q2.5     Q97.5
R2HeterozygositypKb 0.373956 0.09424285 0.1863401 0.5462016
```

```r
## Model 2
marginal_R2_model_2 <- bayes_R2(
  model_2,
  resp = "HeterozygositypKb",
  re_formula = NA
)
marginal_R2_model_2
```

```
                     Estimate  Est.Error      Q2.5     Q97.5
R2HeterozygositypKb 0.2757837 0.08226299 0.1364574 0.4563659
```

```r
# Leave-one-out cross-validation

loo(
  model_1,
  model_2,
  model_null,
  resp="HeterozygositypKb"
)
```

```
Warning: Found 46 observations with a pareto_k > 0.7 in model 'model_1'. We
recommend to set 'moment_match = TRUE' in order to perform moment matching for
problematic observations.

Warning: Found 52 observations with a pareto_k > 0.7 in model 'model_2'. We
recommend to set 'moment_match = TRUE' in order to perform moment matching for
problematic observations.

Warning: Found 2 observations with a pareto_k > 0.7 in model 'model_null'. We
recommend to set 'moment_match = TRUE' in order to perform moment matching for
problematic observations.

Warning: Not all models have the same y variable. ('yhash' attributes do not
match)

Output of model 'model_1':

Computed from 4000 by 257 log-likelihood matrix.

         Estimate   SE
elpd_loo   -243.1 11.7
p_loo       115.8  7.6
looic       486.3 23.4
------
MCSE of elpd_loo is NA.
MCSE and ESS estimates assume MCMC draws (r_eff in [0.2, 1.1]).

Pareto k diagnostic values:
                         Count Pct.    Min. ESS
(-Inf, 0.7]   (good)     211   82.1%   25
   (0.7, 1]   (bad)       44   17.1%   <NA>
   (1, Inf)   (very bad)   2    0.8%   <NA>
See help('pareto-k-diagnostic') for details.

Output of model 'model_2':

Computed from 4000 by 257 log-likelihood matrix.

         Estimate   SE
elpd_loo   -251.3 12.3
p_loo       122.4  8.3
```

```
looic       502.6 24.7
------
MCSE of elpd_loo is NA.
MCSE and ESS estimates assume MCMC draws (r_eff in [0.1, 1.0]).

Pareto k diagnostic values:
                         Count Pct.    Min. ESS
(-Inf, 0.7]   (good)     205   79.8%   24
   (0.7, 1]   (bad)       50   19.5%   <NA>
   (1, Inf)   (very bad)   2    0.8%   <NA>
See help('pareto-k-diagnostic') for details.

Output of model 'model_null':

Computed from 6000 by 257 log-likelihood matrix.

         Estimate   SE
elpd_loo   -127.3 15.6
p_loo        42.4  5.0
looic       254.6 31.3
------
MCSE of elpd_loo is NA.
MCSE and ESS estimates assume MCMC draws (r_eff in [0.5, 1.1]).

Pareto k diagnostic values:
                         Count Pct.    Min. ESS
(-Inf, 0.7]   (good)     255   99.2%   262
   (0.7, 1]   (bad)        2    0.8%   <NA>
   (1, Inf)   (very bad)   0    0.0%   <NA>
See help('pareto-k-diagnostic') for details.

Model comparisons:
           elpd_diff se_diff
model_null    0.0       0.0
model_1    -115.8       9.9
model_2    -124.0       9.5
```

Imputation validation

Plot imputation of spikelet length

```
library(ggh4x)
## model 1 imputation (should have very similar results for model 2)
model_1_Spikeletlength_Ind_pred <-
  fitted(
    model_1,
    allow_new_levels = F,
    resp = "Spikeletlength",
    newdata = dat_modeling
  ) %>%
  as.data.frame() %>%
  mutate(Ind = dat_modeling$Ind)

sd_Spikeletlength <- sd(dat_indwise$Spikelet_length, na.rm = T)
mean_Spikeletlength <- mean(dat_indwise$Spikelet_length, na.rm = T)

model_1_imputation_plot_Spikeletlength_Species <-
  model_1_Spikeletlength_Ind_pred %>%
  as_tibble() %>%
  right_join(
    dat_modeling %>%
      mutate(miss = is.na(Spikelet_length)) %>%
      select(Pop, Ind, miss)
  ) %>%
  right_join(
    dat_indwise %>% select(Ind = IndOG, Species = New.Treatment)
    ) %>%
  mutate(
    Estimate = Estimate * sd_Spikeletlength + mean_Spikeletlength,
    Q2.5 = Q2.5 * sd_Spikeletlength + mean_Spikeletlength,
    Q97.5 = Q97.5 * sd_Spikeletlength + mean_Spikeletlength
  ) %>%
  # set observed values to NA so we only plot the estimates for the missing values
  mutate(
    Estimate = case_when(miss ~ Estimate,.default = NA),
    Q2.5 = case_when(miss ~ Q2.5,.default = NA),
    Q97.5 = case_when(miss ~ Q97.5,.default = NA),
    ) %>%
```

```
  ggplot() +
  geom_pointinterval(
    aes(x = Pop, group = Ind, y = Estimate, ymin = Q2.5, ymax = Q97.5),
    show.legend = F,
    position = position_dodge2(width = 1, preserve = "single")
  ) +
  geom_point(
    aes(x = Pop, group = Ind, y = Spikelet_length),
    data = dat_indwise %>%
      rename(Species = New.Treatment, Ind = IndOG),
    position = position_dodge2(width = 1, preserve = "single"),
    # inherit.aes = F,
    colour = "red",
    fill = NA,
    show.legend = F
  ) +
  facet_wrap(vars(Species), scales = "free_x", drop = TRUE) +
  theme_bw() +
  theme(axis.text.x = element_text(angle = 90)) +
  labs(y = "Spikelet length (mm)")
```

```
Joining with `by = join_by(Ind)`
Joining with `by = join_by(Ind)`
```

```
print(model_1_imputation_plot_Spikeletlength_Species)
```

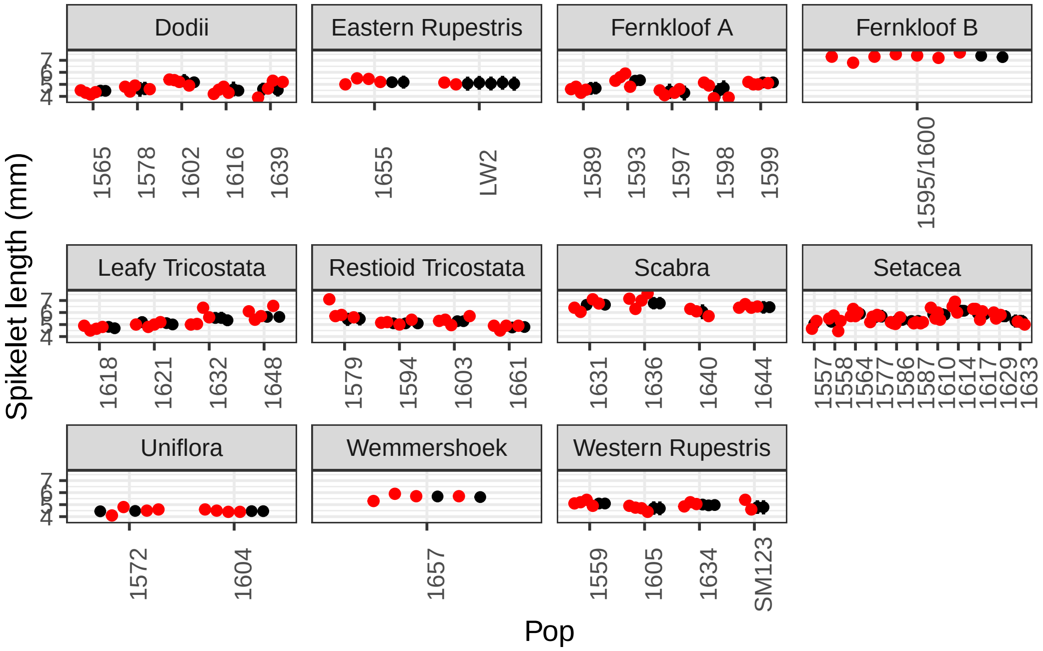


```
ggsave(
  "plots/imputation_spikelet_length_model_1_Species.pdf",
  width = 30,
  height = 20,
  units = "cm",
  plot = model_1_imputation_plot_Spikeletlength_Species
)
# system2("open","plots/imputation_spikelet_length_model_1_Species.pdf")
```

Imputation of spikelet no.

```
Joining with `by = join_by(Ind)`
Joining with `by = join_by(Ind)`
```

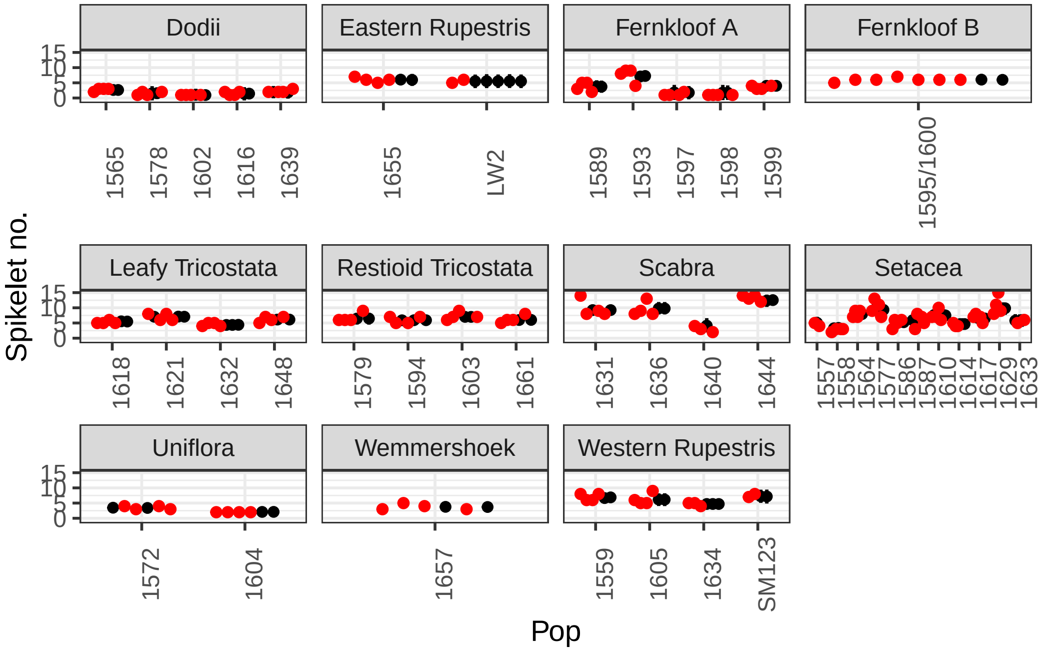

Plot imputation of plant height

```
Joining with `by = join_by(Ind)`
Joining with `by = join_by(Ind)`
```

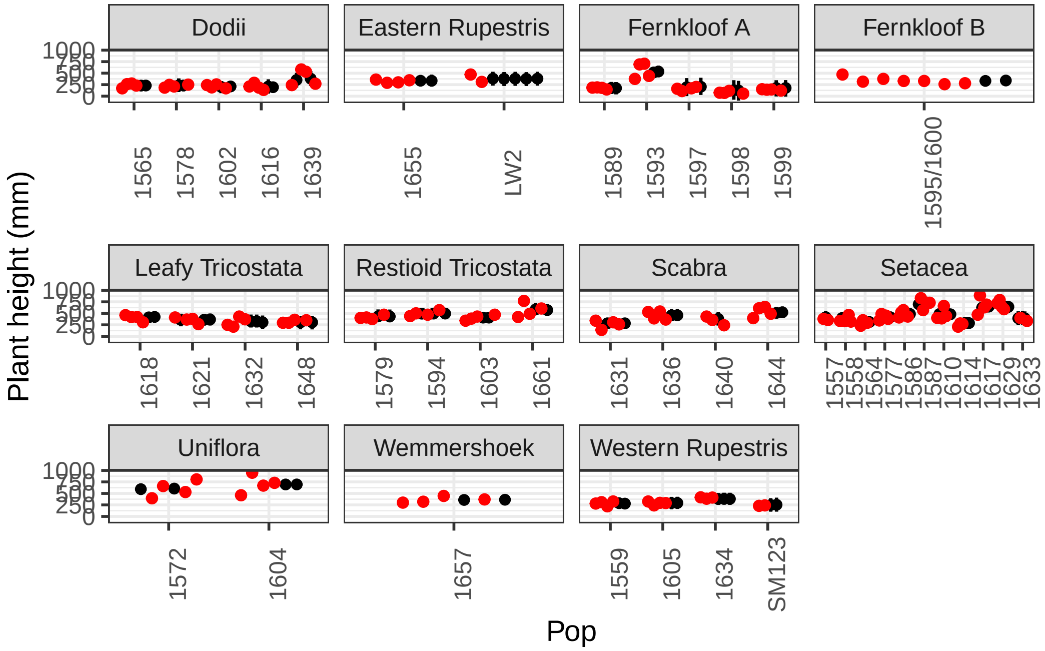

Modeling: Species LME

Model 1 = All vars

```
#|echo: false
model_1_brmsformula <- bf(
  Heterozygosity.pKb ~
    Habitat +
    Origin_isolation_log +
    Climate_variability +
    Rainfall_CV +
    Rainfall_MAP +
    # Dispersal_distance +
    mi(Conspecific_isolation_log) +
    Height_above_drainage +
    mi(Plant_height) +
    mi(Spikelet_no) +
    mi(Spikelet_length) +
    (1 | Species) +
    (1 | Pop)
```

```
) +
  bf(
    Plant_height | mi() ~ 1 + Species + (1 | Pop) + (1 | gr(Ind, cov = Ci))
  ) +
  bf(
    Spikelet_no | mi() ~ 1 + Species + (1 | Pop) + (1 | gr(Ind, cov = Ci))
    ) +
  bf(
    Spikelet_length | mi() ~ 1 + Species + (1 | Pop) + (1 | gr(Ind, cov = Ci))
  ) +
  bf(Conspecific_isolation_log | mi() ~ 1) +
  set_rescor(TRUE)

model_1 <- brm(
  model_1_brmsformula,
  prior=c(
    # SD priors
    prior(std_normal(),class=sd,group=Species,resp=HeterozygositypKb,lb=0),
    prior(std_normal(),class=sd,group=Pop,resp=HeterozygositypKb,lb=0),
    prior(std_normal(),class=sd,group=Pop,resp=Plantheight,lb=0),
    prior(std_normal(),class=sd,group=Pop,resp=Spikeletno,lb=0),
    prior(std_normal(),class=sd,group=Pop,resp=Spikeletlength,lb=0),
    prior(std_normal(),class=sd,group=Ind,resp=Plantheight,lb=0),
    prior(std_normal(),class=sd,group=Ind,resp=Spikeletno,lb=0),
    prior(std_normal(),class=sd,group=Ind,resp=Spikeletlength,lb=0),
    # beta priors
    prior(std_normal(),class=b,resp=HeterozygositypKb),
    # residual corr priors
    prior(lkj_corr_cholesky(2),class=Lrescor)
  ),
  data = dat_modeling,
  family = gaussian(),
  data2 = list(Cp = Cp, Ci = Ci),
  backend = "cmdstanr",
  save_pars = save_pars(all = TRUE),
  iter = 2000,
  cores = 4,
  chains = 4,
  refresh = 250,
  control = list(adapt_delta = 0.995, max_treedepth = 12),
  save_model = ".brms/model_1_SpeciesLME_rescor_C00.stan",
  file = ".brms/model_1_SpeciesLME_rescor_C00",
```

```r
  seed = 42
)
model_1
```

```
 Family: MV(gaussian, gaussian, gaussian, gaussian, gaussian)
  Links: mu = identity
         mu = identity
         mu = identity
         mu = identity
         mu = identity
Formula: Heterozygosity.pKb ~ Habitat + Origin_isolation_log + Climate_variability + Rainfal
         Plant_height | mi() ~ 1 + Species + (1 | Pop) + (1 | gr(Ind, cov = Ci))
         Spikelet_no | mi() ~ 1 + Species + (1 | Pop) + (1 | gr(Ind, cov = Ci))
         Spikelet_length | mi() ~ 1 + Species + (1 | Pop) + (1 | gr(Ind, cov = Ci))
         Conspecific_isolation_log | mi() ~ 1
   Data: dat_modeling (Number of observations: 257)
  Draws: 4 chains, each with iter = 2000; warmup = 1000; thin = 1;
         total post-warmup draws = 4000

Multilevel Hyperparameters:
~Pop (Number of levels: 43)
                                Estimate Est.Error l-95% CI u-95% CI Rhat
sd(HeterozygositypKb_Intercept)     0.83      0.13     0.61     1.12 1.00
sd(Plantheight_Intercept)           0.60      0.16     0.22     0.89 1.01
sd(Spikeletno_Intercept)            0.52      0.19     0.08     0.84 1.02
sd(Spikeletlength_Intercept)        0.24      0.13     0.02     0.49 1.01
                                Bulk_ESS Tail_ESS
sd(HeterozygositypKb_Intercept)     1546     2105
sd(Plantheight_Intercept)            234      252
sd(Spikeletno_Intercept)             238      392
sd(Spikeletlength_Intercept)         495     1468

~Species (Number of levels: 11)
                                Estimate Est.Error l-95% CI u-95% CI Rhat
sd(HeterozygositypKb_Intercept)     1.22      0.34     0.63     1.96 1.00
                                Bulk_ESS Tail_ESS
sd(HeterozygositypKb_Intercept)     1824     1815

~Ind (Number of levels: 257)
                             Estimate Est.Error l-95% CI u-95% CI Rhat Bulk_ESS
sd(Plantheight_Intercept)        0.53      0.30     0.02     1.10 1.02      122
sd(Spikeletno_Intercept)         0.48      0.25     0.03     0.92 1.02      200
```

```
sd(Spikeletlength_Intercept)     0.65      0.16     0.29     0.92 1.01      406
                             Tail_ESS
sd(Plantheight_Intercept)         201
sd(Spikeletno_Intercept)          489
sd(Spikeletlength_Intercept)      486

Regression Coefficients:
                                           Estimate Est.Error l-95% CI
HeterozygositypKb_Intercept                    4.26      0.63     3.06
Plantheight_Intercept                         -0.72      0.55    -1.83
Spikeletno_Intercept                          -1.24      0.48    -2.18
Spikeletlength_Intercept                      -0.84      0.51    -1.84
Conspecificisolationlog_Intercept              0.00      0.07    -0.14
HeterozygositypKb_HabitatWet                   0.85      0.70    -0.58
HeterozygositypKb_Origin_isolation_log         0.08      0.30    -0.52
HeterozygositypKb_Climate_variability         -0.11      0.28    -0.65
HeterozygositypKb_Rainfall_CV                 -0.34      0.31    -0.95
HeterozygositypKb_Rainfall_MAP                 0.31      0.27    -0.23
HeterozygositypKb_Height_above_drainage        0.03      0.16    -0.27
Plantheight_SpeciesEasternRupestris            0.58      0.90    -1.22
Plantheight_SpeciesFernkloofA                 -0.11      0.78    -1.69
Plantheight_SpeciesFernkloofB                  0.43      1.04    -1.69
Plantheight_SpeciesLeafyTricostata             0.52      0.65    -0.82
Plantheight_SpeciesRestioidTricostata          1.22      0.82    -0.41
Plantheight_SpeciesScabra                      0.84      0.79    -0.78
Plantheight_SpeciesSetacea                     1.19      0.63    -0.08
Plantheight_SpeciesUniflora                    2.25      0.91     0.41
Plantheight_SpeciesWemmershoek                 0.62      0.96    -1.30
Plantheight_SpeciesWesternRupestris            0.29      0.78    -1.31
Spikeletno_SpeciesEasternRupestris             1.33      0.80    -0.28
Spikeletno_SpeciesFernkloofA                   0.62      0.63    -0.69
Spikeletno_SpeciesFernkloofB                   1.37      0.92    -0.45
Spikeletno_SpeciesLeafyTricostata              1.29      0.58     0.10
Spikeletno_SpeciesRestioidTricostata           1.52      0.73     0.02
Spikeletno_SpeciesScabra                       2.24      0.69     0.80
Spikeletno_SpeciesSetacea                      1.47      0.56     0.29
Spikeletno_SpeciesUniflora                     0.35      0.82    -1.33
Spikeletno_SpeciesWemmershoek                  0.64      0.83    -0.99
Spikeletno_SpeciesWesternRupestris             1.45      0.69     0.04
Spikeletlength_SpeciesEasternRupestris         0.59      0.79    -0.98
Spikeletlength_SpeciesFernkloofA               0.08      0.65    -1.20
Spikeletlength_SpeciesFernkloofB               3.11      0.86     1.46
Spikeletlength_SpeciesLeafyTricostata          0.84      0.57    -0.27
```

```
Spikeletlength_SpeciesRestioidTricostata          0.66      0.77    -0.82
Spikeletlength_SpeciesScabra                      2.13      0.74     0.62
Spikeletlength_SpeciesSetacea                     1.03      0.55    -0.08
Spikeletlength_SpeciesUniflora                   -0.31      0.82    -1.96
Spikeletlength_SpeciesWemmershoek                 1.17      0.73    -0.28
Spikeletlength_SpeciesWesternRupestris            0.29      0.73    -1.18
HeterozygositypKb_miConspecific_isolation_log     0.03      0.24    -0.43
HeterozygositypKb_miPlant_height                 -0.20      0.21    -0.60
HeterozygositypKb_miSpikelet_no                  -0.18      0.22    -0.63
HeterozygositypKb_miSpikelet_length               0.55      0.22     0.12
                                             u-95% CI Rhat Bulk_ESS Tail_ESS
HeterozygositypKb_Intercept                       5.60 1.00     2497     2699
Plantheight_Intercept                             0.38 1.00      895      698
Spikeletno_Intercept                             -0.27 1.01      961     1147
Spikeletlength_Intercept                          0.20 1.00     1126     1888
Conspecificisolationlog_Intercept                 0.14 1.00     5648     2813
HeterozygositypKb_HabitatWet                      2.19 1.00     2497     2867
HeterozygositypKb_Origin_isolation_log            0.65 1.00     1295     2203
HeterozygositypKb_Climate_variability             0.43 1.00     1556     2318
HeterozygositypKb_Rainfall_CV                     0.27 1.00     1381     2044
HeterozygositypKb_Rainfall_MAP                    0.87 1.00     1335     2072
HeterozygositypKb_Height_above_drainage           0.34 1.00     1467     2427
Plantheight_SpeciesEasternRupestris               2.36 1.00     2031     1499
Plantheight_SpeciesFernkloofA                     1.39 1.01      582      577
Plantheight_SpeciesFernkloofB                     2.50 1.00     2158     2223
Plantheight_SpeciesLeafyTricostata                1.81 1.00     1765     1720
Plantheight_SpeciesRestioidTricostata             2.90 1.00     1722     1238
Plantheight_SpeciesScabra                         2.39 1.00     1488     1317
Plantheight_SpeciesSetacea                        2.40 1.00      948      706
Plantheight_SpeciesUniflora                       4.04 1.00     1482     1142
Plantheight_SpeciesWemmershoek                    2.51 1.00     2479     2561
Plantheight_SpeciesWesternRupestris               1.87 1.00     1631     1184
Spikeletno_SpeciesEasternRupestris                2.89 1.00     1435     1698
Spikeletno_SpeciesFernkloofA                      1.86 1.00     1098     1516
Spikeletno_SpeciesFernkloofB                      3.15 1.00     1918     2267
Spikeletno_SpeciesLeafyTricostata                 2.45 1.00     1647     2340
Spikeletno_SpeciesRestioidTricostata              2.99 1.00     1379     1405
Spikeletno_SpeciesScabra                          3.52 1.00     1303     1639
Spikeletno_SpeciesSetacea                         2.55 1.00     1254     1212
Spikeletno_SpeciesUniflora                        1.90 1.00     1448     1736
Spikeletno_SpeciesWemmershoek                     2.25 1.00     3130     3110
Spikeletno_SpeciesWesternRupestris                2.80 1.00     1564     1623
Spikeletlength_SpeciesEasternRupestris            2.18 1.00     1696     2236
```

```
Spikeletlength_SpeciesFernkloofA                   1.36 1.00     1345     2178
Spikeletlength_SpeciesFernkloofB                   4.79 1.00     1862     2555
Spikeletlength_SpeciesLeafyTricostata              1.97 1.00     1387     2228
Spikeletlength_SpeciesRestioidTricostata           2.17 1.00     1519     2042
Spikeletlength_SpeciesScabra                       3.55 1.00     1790     2162
Spikeletlength_SpeciesSetacea                      2.13 1.00     1843     2700
Spikeletlength_SpeciesUniflora                     1.31 1.00     1610     2362
Spikeletlength_SpeciesWemmershoek                  2.58 1.00     3159     2817
Spikeletlength_SpeciesWesternRupestris             1.78 1.00     1501     1958
HeterozygositypKb_miConspecific_isolation_log      0.50 1.00     1044     1248
HeterozygositypKb_miPlant_height                   0.23 1.01      288      972
HeterozygositypKb_miSpikelet_no                    0.25 1.00      589     1329
HeterozygositypKb_miSpikelet_length                1.00 1.01      521     1095

Further Distributional Parameters:
                              Estimate Est.Error l-95% CI u-95% CI Rhat
sigma_HeterozygositypKb           0.47      0.09     0.35     0.71 1.00
sigma_Plantheight                 0.54      0.05     0.45     0.66 1.00
sigma_Spikeletno                  0.45      0.04     0.38     0.53 1.01
sigma_Spikeletlength              0.39      0.04     0.33     0.47 1.00
sigma_Conspecificisolationlog     1.01      0.05     0.92     1.11 1.00
                              Bulk_ESS Tail_ESS
sigma_HeterozygositypKb            784     1336
sigma_Plantheight                 1066     2023
sigma_Spikeletno                   687     1740
sigma_Spikeletlength               803     1775
sigma_Conspecificisolationlog     6299     2938

Residual Correlations:
                                                   Estimate Est.Error l-95% CI
rescor(HeterozygositypKb,Plantheight)                  0.13      0.27    -0.40
rescor(HeterozygositypKb,Spikeletno)                   0.21      0.23    -0.26
rescor(Plantheight,Spikeletno)                         0.19      0.12    -0.06
rescor(HeterozygositypKb,Spikeletlength)              -0.32      0.23    -0.70
rescor(Plantheight,Spikeletlength)                     0.16      0.11    -0.07
rescor(Spikeletno,Spikeletlength)                      0.06      0.11    -0.14
rescor(HeterozygositypKb,Conspecificisolationlog)     -0.01      0.37    -0.71
rescor(Plantheight,Conspecificisolationlog)            0.11      0.40    -0.62
rescor(Spikeletno,Conspecificisolationlog)             0.15      0.19    -0.24
rescor(Spikeletlength,Conspecificisolationlog)         0.10      0.18    -0.27
                                                   u-95% CI Rhat Bulk_ESS
rescor(HeterozygositypKb,Plantheight)                  0.63 1.01      360
rescor(HeterozygositypKb,Spikeletno)                   0.61 1.00      858
```

```
rescor(Plantheight,Spikeletno)                              0.41 1.00      836
rescor(HeterozygositypKb,Spikeletlength)                    0.19 1.01      558
rescor(Plantheight,Spikeletlength)                          0.38 1.00     1365
rescor(Spikeletno,Spikeletlength)                           0.27 1.00     1958
rescor(HeterozygositypKb,Conspecificisolationlog)           0.70 1.00     1222
rescor(Plantheight,Conspecificisolationlog)                 0.73 1.02      193
rescor(Spikeletno,Conspecificisolationlog)                  0.50 1.01      692
rescor(Spikeletlength,Conspecificisolationlog)              0.44 1.00     1860
                                                        Tail_ESS
rescor(HeterozygositypKb,Plantheight)                        838
rescor(HeterozygositypKb,Spikeletno)                        1843
rescor(Plantheight,Spikeletno)                              1245
rescor(HeterozygositypKb,Spikeletlength)                    1165
rescor(Plantheight,Spikeletlength)                          2059
rescor(Spikeletno,Spikeletlength)                           2520
rescor(HeterozygositypKb,Conspecificisolationlog)           1303
rescor(Plantheight,Conspecificisolationlog)                  254
rescor(Spikeletno,Conspecificisolationlog)                  2038
rescor(Spikeletlength,Conspecificisolationlog)              2698

Draws were sampled using sample(hmc). For each parameter, Bulk_ESS
and Tail_ESS are effective sample size measures, and Rhat is the potential
scale reduction factor on split chains (at convergence, Rhat = 1).
```

```
sink("results/model_1_SpeciesLME_rescor_COO_brmssummary.txt")
print(model_1,digits=3)
sink()

mcmc_plot(model_1)
```

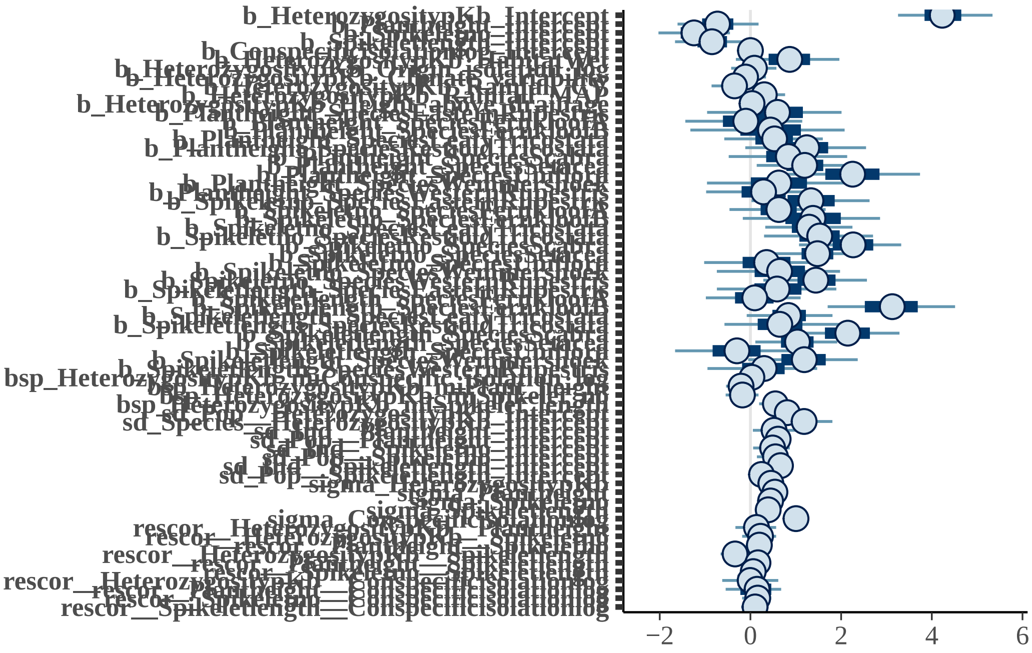

Post-process model 1

```
my_vars <- c(
  "Habitat",
  "Rainfall_MAP",
  "Rainfall_CV",
  "Climate_variability",
  "Spikelet_length",
  "Spikelet_no",
  "Plant_height",
  "Height_above_drainage",
  "Conspecific_isolation_log",
  "Origin_isolation_log"
)


# Note we use avg_slopes (not just slopes()) because we aren't using "by=[variable]" (like be
model_1_slopes <- avg_slopes(
  model_1,
  variables = my_vars,
  re_formula = NA,
  resp = "HeterozygositypKb"
```

```
)
model_1_slopes_draws <- model_1_slopes |>
  posterior_draws()
gc()
```

```
           used  (Mb) gc trigger   (Mb)  max used   (Mb)
Ncells  4409204 235.5    7484034  399.7   7484034  399.7
Vcells 30944829 236.1  138735225 1058.5 198135535 1511.7
```

```
marginal_R2_model_1 <- bayes_R2(
  model_1,
  resp = "HeterozygositypKb",
  re_formula = NA
)
marginal_R2_model_1
```

```
                    Estimate  Est.Error      Q2.5    Q97.5
R2HeterozygositypKb 0.328888 0.09895303 0.1410777 0.525256
```

Model 2: Including interactions with Habitat

We also allow separate intercepts, random effect SDs, and sigmas for each habitat.

```
#|echo: false
model_2_brmsformula<-bf(
  Heterozygosity.pKb ~ 0 +
    Habitat +
    (Climate_variability +
       Rainfall_CV +
       Rainfall_MAP +
       Origin_isolation_log +
       # Dispersal_distance +
       mi(Conspecific_isolation_log) +
       Height_above_drainage +
       mi(Plant_height) +
       mi(Spikelet_no) +
       mi(Spikelet_length)):Habitat +
    (1 | gr(Species, by = Habitat)) +
    (1 | gr(Pop, by = Habitat)),
  sigma ~ 0 + Habitat
```

```
) +
  bf(
    Plant_height | mi() ~ 1 + Species + (1|Pop) + (1 | gr(Ind, cov = Ci))
  ) +
  bf(
    Spikelet_no | mi() ~ 1 + Species + (1|Pop) + (1 | gr(Ind, cov = Ci))
  ) +
  bf(
    Spikelet_length | mi() ~ 1 + Species + (1|Pop) + (1 | gr(Ind, cov = Ci))
  ) +
  # impute Conspecific isolation using dispersal distance. This allows us to
  # incorporate the relationship between the two without strictly assuming them
  # to be equal, because the imputed values will also be informed by the
  # distribution of Conspecific isolation values among the other populations.
  bf(Conspecific_isolation_log | mi() ~ 1) +
  set_rescor(TRUE)

# sets the priors for the coefficients to N(0,2)
# p_manual<-c(prior(normal(0, 2), class = b))

### Run ----------------------------------------------------------------

model_2<-brm(
  model_2_brmsformula,
  prior = c(
    # SD priors
    prior(std_normal(),class=sd,group=Species,resp=HeterozygositypKb,lb=0),
    prior(std_normal(),class=sd,group=Pop,resp=HeterozygositypKb,lb=0),
    prior(std_normal(),class=sd,group=Pop,resp=Plantheight,lb=0),
    prior(std_normal(),class=sd,group=Pop,resp=Spikeletno,lb=0),
    prior(std_normal(),class=sd,group=Pop,resp=Spikeletlength,lb=0),
    prior(std_normal(),class=sd,group=Ind,resp=Plantheight,lb=0),
    prior(std_normal(),class=sd,group=Ind,resp=Spikeletno,lb=0),
    prior(std_normal(),class=sd,group=Ind,resp=Spikeletlength,lb=0),
    # beta priors
    prior(std_normal(),class=b,resp=HeterozygositypKb),
    # residual corr priors
    prior(lkj_corr_cholesky(2),class=Lrescor),
    # non-default sigma prior because we place identity link on sigma
    prior(student_t(3,0,2),resp=HeterozygositypKb,dpar=sigma,lb=0)
  ),
  data = dat_modeling ,
```

```
  family = brmsfamily("gaussian",link_sigma="identity"),
  data2 = list(Cp=Cp,Ci=Ci),
  backend = "cmdstanr",
  save_pars = save_pars(all=TRUE),
  iter = 2000,
  refresh = 250,
  cores = 4,
  chains = 4,
  control = list(adapt_delta = 0.995, max_treedepth = 14),
  save_model = ".brms/model_2_SpeciesLME_rescor_COO.stan",
  file = ".brms/model_2_SpeciesLME_rescor_COO",
  seed = 42
  )
print(summary(model_2))
```

```
 Family: MV(gaussian, gaussian, gaussian, gaussian, gaussian)
  Links: mu = identity; sigma = identity
         mu = identity
         mu = identity
         mu = identity
         mu = identity
Formula: Heterozygosity.pKb ~ 0 + Habitat + (Climate_variability + Rainfall_CV + Rainfall_MA
         sigma ~ 0 + Habitat
         Plant_height | mi() ~ 1 + Species + (1 | Pop) + (1 | gr(Ind, cov = Ci))
         Spikelet_no | mi() ~ 1 + Species + (1 | Pop) + (1 | gr(Ind, cov = Ci))
         Spikelet_length | mi() ~ 1 + Species + (1 | Pop) + (1 | gr(Ind, cov = Ci))
         Conspecific_isolation_log | mi() ~ 1
   Data: dat_modeling (Number of observations: 257)
  Draws: 4 chains, each with iter = 2000; warmup = 1000; thin = 1;
         total post-warmup draws = 4000

Multilevel Hyperparameters:
~Pop (Number of levels: 43)
                                            Estimate Est.Error l-95% CI u-95% CI
sd(HeterozygositypKb_Intercept:HabitatDry)      0.64      0.25     0.28     1.25
sd(HeterozygositypKb_Intercept:HabitatWet)      0.96      0.19     0.66     1.38
sd(Plantheight_Intercept)                       0.62      0.15     0.31     0.90
sd(Spikeletno_Intercept)                        0.52      0.18     0.11     0.83
sd(Spikeletlength_Intercept)                    0.28      0.13     0.03     0.52
                                            Rhat Bulk_ESS Tail_ESS
sd(HeterozygositypKb_Intercept:HabitatDry) 1.00     1203     2047
sd(HeterozygositypKb_Intercept:HabitatWet) 1.00     1393     1795
```

```
sd(Plantheight_Intercept)                 1.00      583      682
sd(Spikeletno_Intercept)                  1.02      378      577
sd(Spikeletlength_Intercept)              1.03      297     1108

~Species (Number of levels: 11)
                                          Estimate Est.Error l-95% CI u-95% CI
sd(HeterozygositypKb_Intercept:HabitatDry)     1.08      0.69     0.05     2.53
sd(HeterozygositypKb_Intercept:HabitatWet)     2.01      0.58     0.95     3.24
                                          Rhat Bulk_ESS Tail_ESS
sd(HeterozygositypKb_Intercept:HabitatDry) 1.00     1675     1571
sd(HeterozygositypKb_Intercept:HabitatWet) 1.00     1344     1072

~Ind (Number of levels: 257)
                             Estimate Est.Error l-95% CI u-95% CI Rhat Bulk_ESS
sd(Plantheight_Intercept)        0.51      0.30     0.02     1.06 1.01      208
sd(Spikeletno_Intercept)         0.48      0.24     0.03     0.92 1.02      253
sd(Spikeletlength_Intercept)     0.60      0.18     0.14     0.90 1.04      216
                             Tail_ESS
sd(Plantheight_Intercept)         625
sd(Spikeletno_Intercept)          704
sd(Spikeletlength_Intercept)      123

Regression Coefficients:
                                                        Estimate Est.Error
Plantheight_Intercept                                      -0.71      0.56
Spikeletno_Intercept                                       -1.26      0.50
Spikeletlength_Intercept                                   -0.89      0.51
Conspecificisolationlog_Intercept                           0.00      0.07
HeterozygositypKb_HabitatDry                                2.40      0.92
HeterozygositypKb_HabitatWet                                3.40      0.85
HeterozygositypKb_HabitatDry:Climate_variability           -0.22      0.43
HeterozygositypKb_HabitatWet:Climate_variability            0.00      0.37
HeterozygositypKb_HabitatDry:Rainfall_CV                   -0.32      0.54
HeterozygositypKb_HabitatWet:Rainfall_CV                   -0.44      0.45
HeterozygositypKb_HabitatDry:Rainfall_MAP                  -0.29      0.62
HeterozygositypKb_HabitatWet:Rainfall_MAP                   0.32      0.34
HeterozygositypKb_HabitatDry:Origin_isolation_log          -0.08      0.37
HeterozygositypKb_HabitatWet:Origin_isolation_log           0.30      0.46
HeterozygositypKb_HabitatDry:Height_above_drainage          0.13      0.33
HeterozygositypKb_HabitatWet:Height_above_drainage         -0.00      0.20
sigma_HeterozygositypKb_HabitatDry                          0.46      0.10
sigma_HeterozygositypKb_HabitatWet                          0.49      0.11
Plantheight_SpeciesEasternRupestris                         0.57      0.90
```

Plantheight_SpeciesFernkloofA	-0.15	0.83
Plantheight_SpeciesFernkloofB	0.42	1.05
Plantheight_SpeciesLeafyTricostata	0.51	0.66
Plantheight_SpeciesRestioidTricostata	1.24	0.81
Plantheight_SpeciesScabra	0.84	0.80
Plantheight_SpeciesSetacea	1.18	0.63
Plantheight_SpeciesUniflora	2.23	0.93
Plantheight_SpeciesWemmershoek	0.58	0.94
Plantheight_SpeciesWesternRupestris	0.32	0.77
Spikeletno_SpeciesEasternRupestris	1.35	0.80
Spikeletno_SpeciesFernkloofA	0.65	0.67
Spikeletno_SpeciesFernkloofB	1.40	0.93
Spikeletno_SpeciesLeafyTricostata	1.31	0.59
Spikeletno_SpeciesRestioidTricostata	1.57	0.73
Spikeletno_SpeciesScabra	2.28	0.71
Spikeletno_SpeciesSetacea	1.51	0.57
Spikeletno_SpeciesUniflora	0.38	0.83
Spikeletno_SpeciesWemmershoek	0.67	0.82
Spikeletno_SpeciesWesternRupestris	1.47	0.70
Spikeletlength_SpeciesEasternRupestris	0.65	0.78
Spikeletlength_SpeciesFernkloofA	0.19	0.65
Spikeletlength_SpeciesFernkloofB	3.18	0.86
Spikeletlength_SpeciesLeafyTricostata	0.86	0.55
Spikeletlength_SpeciesRestioidTricostata	0.74	0.77
Spikeletlength_SpeciesScabra	2.21	0.70
Spikeletlength_SpeciesSetacea	1.08	0.53
Spikeletlength_SpeciesUniflora	-0.19	0.81
Spikeletlength_SpeciesWemmershoek	1.20	0.72
Spikeletlength_SpeciesWesternRupestris	0.34	0.71
HeterozygositypKb_HabitatDry:miConspecific_isolation_log	-0.07	0.38
HeterozygositypKb_HabitatWet:miConspecific_isolation_log	0.02	0.26
HeterozygositypKb_HabitatDry:miPlant_height	-0.27	0.24
HeterozygositypKb_HabitatWet:miPlant_height	-0.22	0.25
HeterozygositypKb_HabitatDry:miSpikelet_no	-0.19	0.23
HeterozygositypKb_HabitatWet:miSpikelet_no	-0.23	0.28
HeterozygositypKb_HabitatDry:miSpikelet_length	0.45	0.24
HeterozygositypKb_HabitatWet:miSpikelet_length	0.38	0.28

	l-95% CI	u-95% CI	Rhat
Plantheight_Intercept	-1.82	0.40	1.00
Spikeletno_Intercept	-2.28	-0.22	1.01
Spikeletlength_Intercept	-1.95	0.08	1.01
Conspecificisolationlog_Intercept	-0.14	0.14	1.00
HeterozygositypKb_HabitatDry	0.39	3.81	1.00

HeterozygositypKb_HabitatWet	1.62	4.92	1.00
HeterozygositypKb_HabitatDry:Climate_variability	-1.11	0.66	1.00
HeterozygositypKb_HabitatWet:Climate_variability	-0.72	0.76	1.00
HeterozygositypKb_HabitatDry:Rainfall_CV	-1.40	0.76	1.00
HeterozygositypKb_HabitatWet:Rainfall_CV	-1.31	0.45	1.00
HeterozygositypKb_HabitatDry:Rainfall_MAP	-1.49	0.97	1.00
HeterozygositypKb_HabitatWet:Rainfall_MAP	-0.35	0.99	1.00
HeterozygositypKb_HabitatDry:Origin_isolation_log	-0.87	0.61	1.00
HeterozygositypKb_HabitatWet:Origin_isolation_log	-0.63	1.16	1.00
HeterozygositypKb_HabitatDry:Height_above_drainage	-0.48	0.85	1.00
HeterozygositypKb_HabitatWet:Height_above_drainage	-0.40	0.38	1.00
sigma_HeterozygositypKb_HabitatDry	0.33	0.71	1.00
sigma_HeterozygositypKb_HabitatWet	0.36	0.75	1.00
Plantheight_SpeciesEasternRupestris	-1.21	2.32	1.00
Plantheight_SpeciesFernkloofA	-1.80	1.52	1.01
Plantheight_SpeciesFernkloofB	-1.59	2.50	1.00
Plantheight_SpeciesLeafyTricostata	-0.81	1.81	1.00
Plantheight_SpeciesRestioidTricostata	-0.42	2.89	1.00
Plantheight_SpeciesScabra	-0.82	2.40	1.00
Plantheight_SpeciesSetacea	-0.05	2.43	1.01
Plantheight_SpeciesUniflora	0.39	4.02	1.00
Plantheight_SpeciesWemmershoek	-1.27	2.43	1.00
Plantheight_SpeciesWesternRupestris	-1.24	1.83	1.00
Spikeletno_SpeciesEasternRupestris	-0.19	2.98	1.00
Spikeletno_SpeciesFernkloofA	-0.69	1.98	1.01
Spikeletno_SpeciesFernkloofB	-0.46	3.28	1.00
Spikeletno_SpeciesLeafyTricostata	0.13	2.48	1.00
Spikeletno_SpeciesRestioidTricostata	0.15	3.06	1.00
Spikeletno_SpeciesScabra	0.78	3.68	1.01
Spikeletno_SpeciesSetacea	0.32	2.61	1.01
Spikeletno_SpeciesUniflora	-1.27	2.05	1.00
Spikeletno_SpeciesWemmershoek	-0.97	2.28	1.00
Spikeletno_SpeciesWesternRupestris	0.04	2.85	1.00
Spikeletlength_SpeciesEasternRupestris	-0.89	2.26	1.00
Spikeletlength_SpeciesFernkloofA	-1.13	1.43	1.01
Spikeletlength_SpeciesFernkloofB	1.43	4.89	1.00
Spikeletlength_SpeciesLeafyTricostata	-0.22	1.94	1.00
Spikeletlength_SpeciesRestioidTricostata	-0.77	2.31	1.00
Spikeletlength_SpeciesScabra	0.83	3.62	1.01
Spikeletlength_SpeciesSetacea	-0.03	2.09	1.00
Spikeletlength_SpeciesUniflora	-1.80	1.45	1.00
Spikeletlength_SpeciesWemmershoek	-0.21	2.64	1.00
Spikeletlength_SpeciesWesternRupestris	-1.07	1.82	1.00

```
HeterozygositypKb_HabitatDry:miConspecific_isolation_log    -0.83     0.68 1.00
HeterozygositypKb_HabitatWet:miConspecific_isolation_log    -0.49     0.55 1.00
HeterozygositypKb_HabitatDry:miPlant_height                 -0.73     0.22 1.00
HeterozygositypKb_HabitatWet:miPlant_height                 -0.70     0.28 1.00
HeterozygositypKb_HabitatDry:miSpikelet_no                  -0.69     0.23 1.00
HeterozygositypKb_HabitatWet:miSpikelet_no                  -0.80     0.28 1.01
HeterozygositypKb_HabitatDry:miSpikelet_length              -0.01     0.93 1.01
HeterozygositypKb_HabitatWet:miSpikelet_length              -0.19     0.91 1.02
                                                         Bulk_ESS Tail_ESS
Plantheight_Intercept                                         958     1457
Spikeletno_Intercept                                          972     1226
Spikeletlength_Intercept                                      971     1234
Conspecificisolationlog_Intercept                            5391     3353
HeterozygositypKb_HabitatDry                                 1721     2440
HeterozygositypKb_HabitatWet                                 1382     1561
HeterozygositypKb_HabitatDry:Climate_variability             2199     2262
HeterozygositypKb_HabitatWet:Climate_variability             1912     2348
HeterozygositypKb_HabitatDry:Rainfall_CV                     1905     1815
HeterozygositypKb_HabitatWet:Rainfall_CV                     1760     2024
HeterozygositypKb_HabitatDry:Rainfall_MAP                    2495     2393
HeterozygositypKb_HabitatWet:Rainfall_MAP                    1633     2010
HeterozygositypKb_HabitatDry:Origin_isolation_log            1611     2017
HeterozygositypKb_HabitatWet:Origin_isolation_log            1686     2117
HeterozygositypKb_HabitatDry:Height_above_drainage           1324     1723
HeterozygositypKb_HabitatWet:Height_above_drainage           1802     1848
sigma_HeterozygositypKb_HabitatDry                            957     1294
sigma_HeterozygositypKb_HabitatWet                            753     1155
Plantheight_SpeciesEasternRupestris                          1733     2030
Plantheight_SpeciesFernkloofA                                 487     1060
Plantheight_SpeciesFernkloofB                                1621     1967
Plantheight_SpeciesLeafyTricostata                           1688     1636
Plantheight_SpeciesRestioidTricostata                        1681     1946
Plantheight_SpeciesScabra                                    1267     1636
Plantheight_SpeciesSetacea                                    642     1178
Plantheight_SpeciesUniflora                                  1475     1698
Plantheight_SpeciesWemmershoek                               2304     2305
Plantheight_SpeciesWesternRupestris                          1583     1646
Spikeletno_SpeciesEasternRupestris                           1496     1770
Spikeletno_SpeciesFernkloofA                                 1046     1489
Spikeletno_SpeciesFernkloofB                                 1735     1803
Spikeletno_SpeciesLeafyTricostata                            1521     1636
Spikeletno_SpeciesRestioidTricostata                         1295     1492
Spikeletno_SpeciesScabra                                      999     1169
```

```
Spikeletno_SpeciesSetacea                                  1348     1730
Spikeletno_SpeciesUniflora                                 1555     1832
Spikeletno_SpeciesWemmershoek                              2845     2924
Spikeletno_SpeciesWesternRupestris                         1513     1618
Spikeletlength_SpeciesEasternRupestris                     1616     1864
Spikeletlength_SpeciesFernkloofA                           1278     1629
Spikeletlength_SpeciesFernkloofB                           1754     1641
Spikeletlength_SpeciesLeafyTricostata                      1144     1453
Spikeletlength_SpeciesRestioidTricostata                   1340     1385
Spikeletlength_SpeciesScabra                               1380     1725
Spikeletlength_SpeciesSetacea                              1750     2039
Spikeletlength_SpeciesUniflora                             1506     1854
Spikeletlength_SpeciesWemmershoek                          2411     2140
Spikeletlength_SpeciesWesternRupestris                     1339     1599
HeterozygositypKb_HabitatDry:miConspecific_isolation_log   1343     2246
HeterozygositypKb_HabitatWet:miConspecific_isolation_log    729     1086
HeterozygositypKb_HabitatDry:miPlant_height                 553     1441
HeterozygositypKb_HabitatWet:miPlant_height                 452     1200
HeterozygositypKb_HabitatDry:miSpikelet_no                  692     1035
HeterozygositypKb_HabitatWet:miSpikelet_no                  563      781
HeterozygositypKb_HabitatDry:miSpikelet_length              576     1160
HeterozygositypKb_HabitatWet:miSpikelet_length              484      935

Further Distributional Parameters:
                              Estimate Est.Error l-95% CI u-95% CI Rhat
sigma_Plantheight                 0.54      0.05     0.44     0.66 1.00
sigma_Spikeletno                  0.45      0.04     0.38     0.54 1.01
sigma_Spikeletlength              0.40      0.04     0.33     0.48 1.01
sigma_Conspecificisolationlog     1.01      0.05     0.92     1.11 1.00
                              Bulk_ESS Tail_ESS
sigma_Plantheight                 1002     1689
sigma_Spikeletno                   807     2066
sigma_Spikeletlength               547     1176
sigma_Conspecificisolationlog     4661     3032

Residual Correlations:
                                                    Estimate Est.Error l-95% CI
rescor(HeterozygositypKb,Plantheight)                   0.18      0.30    -0.41
rescor(HeterozygositypKb,Spikeletno)                    0.26      0.24    -0.25
rescor(Plantheight,Spikeletno)                          0.19      0.12    -0.06
rescor(HeterozygositypKb,Spikeletlength)               -0.15      0.27    -0.61
rescor(Plantheight,Spikeletlength)                      0.16      0.11    -0.07
rescor(Spikeletno,Spikeletlength)                       0.06      0.11    -0.15
```

```
rescor(HeterozygositypKb,Conspecificisolationlog)     0.01      0.39    -0.72
rescor(Plantheight,Conspecificisolationlog)           0.08      0.40    -0.63
rescor(Spikeletno,Conspecificisolationlog)            0.15      0.20    -0.24
rescor(Spikeletlength,Conspecificisolationlog)        0.14      0.18    -0.23
                                                  u-95% CI Rhat Bulk_ESS
rescor(HeterozygositypKb,Plantheight)                 0.70 1.00      527
rescor(HeterozygositypKb,Spikeletno)                  0.68 1.00      680
rescor(Plantheight,Spikeletno)                        0.41 1.00     1125
rescor(HeterozygositypKb,Spikeletlength)              0.41 1.01      558
rescor(Plantheight,Spikeletlength)                    0.38 1.00      664
rescor(Spikeletno,Spikeletlength)                     0.28 1.00     1827
rescor(HeterozygositypKb,Conspecificisolationlog)     0.73 1.00      816
rescor(Plantheight,Conspecificisolationlog)           0.71 1.02      120
rescor(Spikeletno,Conspecificisolationlog)            0.50 1.01      353
rescor(Spikeletlength,Conspecificisolationlog)        0.48 1.00     1381
                                                  Tail_ESS
rescor(HeterozygositypKb,Plantheight)                 1321
rescor(HeterozygositypKb,Spikeletno)                  1209
rescor(Plantheight,Spikeletno)                        1928
rescor(HeterozygositypKb,Spikeletlength)               889
rescor(Plantheight,Spikeletlength)                    2198
rescor(Spikeletno,Spikeletlength)                     2578
rescor(HeterozygositypKb,Conspecificisolationlog)     1180
rescor(Plantheight,Conspecificisolationlog)            444
rescor(Spikeletno,Conspecificisolationlog)            1745
rescor(Spikeletlength,Conspecificisolationlog)        2179

Draws were sampled using sample(hmc). For each parameter, Bulk_ESS
and Tail_ESS are effective sample size measures, and Rhat is the potential
scale reduction factor on split chains (at convergence, Rhat = 1).
```

```
get_prior(model_2)
```

```
                prior     class                               coef   group
               lkj(1)    rescor
 student_t(3, 0, 2.5) Intercept
 student_t(3, 0, 2.5)     sigma
               (flat)         b
               (flat)         b                         HabitatDry
               (flat)         b        HabitatDry:Climate_variability
               (flat)         b      HabitatDry:Height_above_drainage
               (flat)         b HabitatDry:miConspecific_isolation_log
```

(flat)	b	HabitatDry:miPlant_height	
(flat)	b	HabitatDry:miSpikelet_length	
(flat)	b	HabitatDry:miSpikelet_no	
(flat)	b	HabitatDry:Origin_isolation_log	
(flat)	b	HabitatDry:Rainfall_CV	
(flat)	b	HabitatDry:Rainfall_MAP	
(flat)	b	HabitatWet	
(flat)	b	HabitatWet:Climate_variability	
(flat)	b	HabitatWet:Height_above_drainage	
(flat)	b	HabitatWet:miConspecific_isolation_log	
(flat)	b	HabitatWet:miPlant_height	
(flat)	b	HabitatWet:miSpikelet_length	
(flat)	b	HabitatWet:miSpikelet_no	
(flat)	b	HabitatWet:Origin_isolation_log	
(flat)	b	HabitatWet:Rainfall_CV	
(flat)	b	HabitatWet:Rainfall_MAP	
student_t(3, 0, 2.5)	sd		
student_t(3, 0, 2.5)	sd		Pop
student_t(3, 0, 2.5)	sd	Intercept	Pop
student_t(3, 0, 2.5)	sd		Species
student_t(3, 0, 2.5)	sd	Intercept	Species
(flat)	b		
(flat)	b	HabitatDry	
(flat)	b	HabitatWet	
(flat)	b		
(flat)	b	SpeciesEasternRupestris	
(flat)	b	SpeciesFernkloofA	
(flat)	b	SpeciesFernkloofB	
(flat)	b	SpeciesLeafyTricostata	
(flat)	b	SpeciesRestioidTricostata	
(flat)	b	SpeciesScabra	
(flat)	b	SpeciesSetacea	
(flat)	b	SpeciesUniflora	
(flat)	b	SpeciesWemmershoek	
(flat)	b	SpeciesWesternRupestris	
student_t(3, 0, 2.5)	Intercept		
student_t(3, 0, 2.5)	sd		
student_t(3, 0, 2.5)	sd		Ind
student_t(3, 0, 2.5)	sd	Intercept	Ind
student_t(3, 0, 2.5)	sd		Pop
student_t(3, 0, 2.5)	sd	Intercept	Pop
student_t(3, 0, 2.5)	sigma		
(flat)	b		

```
               (flat)         b              SpeciesEasternRupestris
               (flat)         b                    SpeciesFernkloofA
               (flat)         b                    SpeciesFernkloofB
               (flat)         b               SpeciesLeafyTricostata
               (flat)         b            SpeciesRestioidTricostata
               (flat)         b                        SpeciesScabra
               (flat)         b                       SpeciesSetacea
               (flat)         b                      SpeciesUniflora
               (flat)         b                   SpeciesWemmershoek
               (flat)         b              SpeciesWesternRupestris
 student_t(3, 0, 2.5) Intercept
 student_t(3, 0, 2.5)        sd
 student_t(3, 0, 2.5)        sd                                          Ind
 student_t(3, 0, 2.5)        sd                            Intercept     Ind
 student_t(3, 0, 2.5)        sd                                          Pop
 student_t(3, 0, 2.5)        sd                            Intercept     Pop
 student_t(3, 0, 2.5)     sigma
               (flat)         b
               (flat)         b              SpeciesEasternRupestris
               (flat)         b                    SpeciesFernkloofA
               (flat)         b                    SpeciesFernkloofB
               (flat)         b               SpeciesLeafyTricostata
               (flat)         b            SpeciesRestioidTricostata
               (flat)         b                        SpeciesScabra
               (flat)         b                       SpeciesSetacea
               (flat)         b                      SpeciesUniflora
               (flat)         b                   SpeciesWemmershoek
               (flat)         b              SpeciesWesternRupestris
 student_t(3, 0, 2.5) Intercept
 student_t(3, 0, 2.5)        sd
 student_t(3, 0, 2.5)        sd                                          Ind
 student_t(3, 0, 2.5)        sd                            Intercept     Ind
 student_t(3, 0, 2.5)        sd                                          Pop
 student_t(3, 0, 2.5)        sd                            Intercept     Pop
 student_t(3, 0, 2.5)     sigma
                    resp  dpar nlpar lb ub tag       source
                                                    default
 Conspecificisolationlog                            default
 Conspecificisolationlog              0             default
       HeterozygositypKb                            default
       HeterozygositypKb                       (vectorized)
       HeterozygositypKb                       (vectorized)
       HeterozygositypKb                       (vectorized)
```

HeterozygositypKb		(vectorized)
HeterozygositypKb		(vectorized)
HeterozygositypKb		(vectorized)
HeterozygositypKb		(vectorized)
HeterozygositypKb		(vectorized)
HeterozygositypKb		(vectorized)
HeterozygositypKb		(vectorized)
HeterozygositypKb		(vectorized)
HeterozygositypKb		(vectorized)
HeterozygositypKb		(vectorized)
HeterozygositypKb		(vectorized)
HeterozygositypKb		(vectorized)
HeterozygositypKb		(vectorized)
HeterozygositypKb		(vectorized)
HeterozygositypKb		(vectorized)
HeterozygositypKb		(vectorized)
HeterozygositypKb		(vectorized)
HeterozygositypKb	0	default
HeterozygositypKb	0	(vectorized)
HeterozygositypKb	0	(vectorized)
HeterozygositypKb	0	(vectorized)
HeterozygositypKb	0	(vectorized)
HeterozygositypKb sigma		default
HeterozygositypKb sigma		(vectorized)
HeterozygositypKb sigma		(vectorized)
Plantheight		default
Plantheight		(vectorized)
Plantheight		(vectorized)
Plantheight		(vectorized)
Plantheight		(vectorized)
Plantheight		(vectorized)
Plantheight		(vectorized)
Plantheight		(vectorized)
Plantheight		(vectorized)
Plantheight		(vectorized)
Plantheight		(vectorized)
Plantheight		default
Plantheight	0	default
Plantheight	0	(vectorized)
Plantheight	0	(vectorized)
Plantheight	0	(vectorized)
Plantheight	0	(vectorized)
Plantheight	0	default

```
 Spikeletlength                      default
 Spikeletlength                 (vectorized)
 Spikeletlength                 (vectorized)
 Spikeletlength                 (vectorized)
 Spikeletlength                 (vectorized)
 Spikeletlength                 (vectorized)
 Spikeletlength                 (vectorized)
 Spikeletlength                 (vectorized)
 Spikeletlength                 (vectorized)
 Spikeletlength                 (vectorized)
 Spikeletlength                 (vectorized)
 Spikeletlength                      default
 Spikeletlength           0          default
 Spikeletlength           0     (vectorized)
 Spikeletlength           0     (vectorized)
 Spikeletlength           0     (vectorized)
 Spikeletlength           0     (vectorized)
 Spikeletlength           0          default
     Spikeletno                      default
     Spikeletno                 (vectorized)
     Spikeletno                 (vectorized)
     Spikeletno                 (vectorized)
     Spikeletno                 (vectorized)
     Spikeletno                 (vectorized)
     Spikeletno                 (vectorized)
     Spikeletno                 (vectorized)
     Spikeletno                 (vectorized)
     Spikeletno                 (vectorized)
     Spikeletno                 (vectorized)
     Spikeletno                      default
     Spikeletno           0          default
     Spikeletno           0     (vectorized)
     Spikeletno           0     (vectorized)
     Spikeletno           0     (vectorized)
     Spikeletno           0     (vectorized)
     Spikeletno           0          default
```

```
sink("results/model_2_SpeciesLME_brmssummary_rescor_C00.txt")
print(model_2,digits=3)
sink()

# system2("open","results/model_2_SpeciesLME_brmssummary_rescor_C00.txt")
```

Post-process model 2.

```
#|echo: false


marginal_R2_model_2<-bayes_R2(model_2,
                              resp="HeterozygositypKb",
                              re_formula=NA)
marginal_R2_model_2
```

```
                     Estimate Est.Error     Q2.5     Q97.5
R2HeterozygositypKb 0.3136421 0.1137591 0.128388 0.5441362
```

```
### Slopes ----
## note: Always specify the variables explicitly to avoid computing
## estimates for the grouping variables (i.e., random effects)!
vars_model<-c(
  "Rainfall_MAP",
  "Rainfall_CV",
  "Climate_variability",
  "Spikelet_length",
  "Spikelet_no",
  "Plant_height",
  # "Dispersal_distance",
  "Conspecific_isolation_log",
  "Height_above_drainage",
  "Origin_isolation_log"
  )

model_2_marginal_habitat_slopes<-avg_slopes(
  model_2,
  variables = vars_model,
  by = "Habitat",
  re_formula = NA,
  # re_formula = NULL,
  resp = "HeterozygositypKb"
  )

sink("results/model_2_COO_SpeciesLME_slopes.txt")
print(model_2_marginal_habitat_slopes)
sink()
```

```
model_2_marginal_habitat_slopes_draws <-
  model_2_marginal_habitat_slopes |>
  posterior_draws()
```

Plot Models 1 and 2 together

```
from_names <- c(
  "Habitat",
  "Spikelet_length",
  "Spikelet_no",
  "Plant_height",
  "Climate_variability",
  "Rainfall_CV",
  "Rainfall_MAP",
  "Height_above_drainage",
  "Conspecific_isolation_log",
  "Origin_isolation_log"
)

to_names <- c(
  "Habitat: Wet - Dry",
  "SPL",
  "SPN",
  "PLH",
  "CII",
  "CVAP",
  "MAP",
  "HAND",
  "ISO_C",
  "ISO_O"
)

unique(model_1_slopes_draws$term)
```

```
 [1] "Climate_variability"       "Conspecific_isolation_log"
 [3] "Habitat"                   "Height_above_drainage"
 [5] "Origin_isolation_log"      "Plant_height"
 [7] "Rainfall_CV"               "Rainfall_MAP"
 [9] "Spikelet_length"           "Spikelet_no"
```

```
model_1_slopes_draws$Term <- model_1_slopes_draws$term
model_2_marginal_habitat_slopes_draws$Term <- model_2_marginal_habitat_slopes_draws$term
for(i in seq_along(from_names)){
  model_1_slopes_draws$Term[model_1_slopes_draws$term==from_names[i]] <- to_names[i]
  model_2_marginal_habitat_slopes_draws$Term[model_2_marginal_habitat_slopes_draws$term==from
}

unique(model_1_slopes_draws$Term)
```

```
 [1] "CII"                    "ISO_C"                  "Habitat: Wet - Dry"
 [4] "HAND"                   "ISO_O"                  "PLH"
 [7] "CVAP"                   "MAP"                    "SPL"
[10] "SPN"
```

```
unique(model_2_marginal_habitat_slopes_draws$Term)
```

```
[1] "CII"   "ISO_C" "HAND"  "ISO_O" "PLH"   "CVAP"  "MAP"   "SPL"   "SPN"
```

```
palette_wetdry<-c("#4dbbd5","#e64b35")
palette_wetdry_3 <- c(palette_wetdry, "darkgreen")
palette_wetdry_3 <- scales::alpha(palette_wetdry_3, 0.7)
p_both <- bind_rows(
  model_1_slopes_draws %>%
    mutate(Model = "Model 1"),
  model_2_marginal_habitat_slopes_draws %>%
    mutate(Model = "Model 2")
) %>%
  mutate(Term = fct_relevel(Term, rev(to_names)), Model = fct_rev(Model)) %>%
  mutate(Habitat = case_when(is.na(Habitat) ~ "", .default = Habitat)) %>%
  mutate(Habitat = fct_relevel(Habitat, c("Wet", "Dry", ""))) %>%
  ggplot(aes(
    x = draw,
    y = Term,
    shape = Model,
    fill = Habitat
  )) +
  geom_vline(xintercept = 0, linetype = 3) +
  stat_eye(
    position = position_dodge(width = .7),
    normalize = "xy",
```

```
    scale = .65,
    point_fill = "white"
  ) +
  theme_bw() +
  theme(axis.text.y = element_text(size = 12),
        legend.text = element_text(size = 10)) +
  guides(
    fill = guide_legend(override.aes = list(
      size = NA,
      shape = NA,
      fill = c(palette_wetdry, NA)
    )),
    shape = guide_legend(
      override.aes = list(size = 6, linetype = NA),
      reverse = TRUE
    )
  ) +
  scale_shape_manual(values = c(21:23)) +
  scale_fill_manual(values = palette_wetdry_3) +
  labs(x = "Effect on IGH", y = "Term") +
  coord_cartesian(xlim = c(-3, 3))
print(p_both)
```

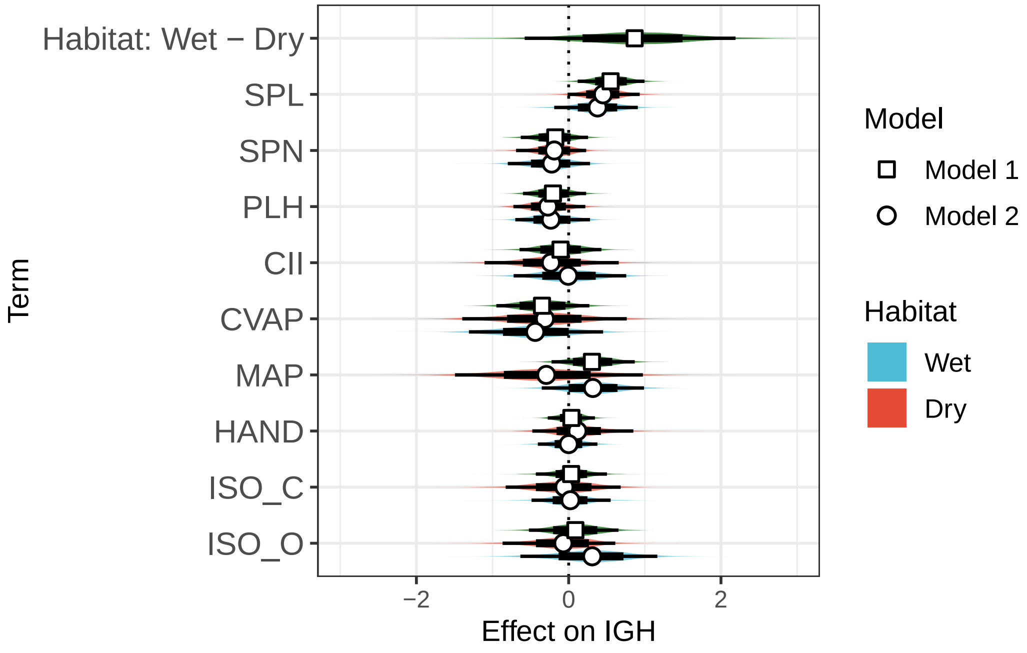

```r
ggsave("plots/MarginalEffects_slabs_models_1and2_SpeciesLME_COO.pdf",
       width=24,height=20,units="cm",plot=p_both)
ggsave("plots/MarginalEffects_slabs_models_1and2_SpeciesLME_COO.png",
       width=24,height=20,units="cm",plot=p_both)
# system2("open","plots/MarginalEffects_slabs_models_1and2_SpeciesLME_COO.pdf")
```
